# ProteinShader: illustrative rendering of macromolecules

**DOI:** 10.1186/1472-6807-9-19

**Published:** 2009-03-30

**Authors:** Joseph R Weber

**Affiliations:** 1 Extension School, Harvard University, Cambridge, Massachusetts 02138, USA

## Abstract

**Background:**

Cartoon-style illustrative renderings of proteins can help clarify structural features that are obscured by space filling or balls and sticks style models, and recent advances in programmable graphics cards offer many new opportunities for improving illustrative renderings.

**Results:**

The ProteinShader program, a new tool for macromolecular visualization, uses information from Protein Data Bank files to produce illustrative renderings of proteins that approximate what an artist might create by hand using pen and ink. A combination of Hermite and spherical linear interpolation is used to draw smooth, gradually rotating three-dimensional tubes and ribbons with a repeating pattern of texture coordinates, which allows the application of texture mapping, real-time halftoning, and smooth edge lines. This free platform-independent open-source program is written primarily in Java, but also makes extensive use of the OpenGL Shading Language to modify the graphics pipeline.

**Conclusion:**

By programming to the graphics processor unit, ProteinShader is able to produce high quality images and illustrative rendering effects in real-time. The main feature that distinguishes ProteinShader from other free molecular visualization tools is its use of texture mapping techniques that allow two-dimensional images to be mapped onto the curved three-dimensional surfaces of ribbons and tubes with minimum distortion of the images.

## Background

The study of protein structure is an intensely active area of research. The number of proteins for which a three-dimensional structure has been solved has increased exponentially in recent years, and there are currently over 56,000 entries in the Protein Data Bank (PDB [1,2]), a publicly accessible single worldwide archive of structural data for biological macromolecules. The three-dimensional structure of a protein determines what other molecules it is capable of binding and interacting with, so a deep understanding of protein structure is critical for predicting protein function and for designing drugs that interact with proteins.

The basic building blocks of protein, amino acids, are small enough that they can be easily understood using simple balls and sticks models that show every atom and bond. Proteins, however, are typically composed of hundreds or even thousands of amino acids, making detailed three-dimensional models very difficult to understand. Fortunately, artistic ribbon representations of the protein backbone can be used to clarify regions of secondary structure, for example by using spiral ribbons for α-helices and arrows for β-strands [3], and simplified cartoon-style models using ribbons and tubes are commonly used in molecular visualization programs.

The World Index of BioMolecular Visualization Resources web page [4] has an extensive listing of free molecular visualization programs that can run on ordinary personal computers. One of the most influential of these is RasMol [5], which is written in the C programming language, and is available on Windows, Macintosh, Linux, and Unix platforms [6,7]. RasMol's success was apparently due to an excellent compromise between rendering speed and image quality so that even large proteins can be rotated in real time [8]. More recently, Java based molecular visualization tools have become popular, in large part because of Java's platform independence, and a typical PDB web page for a protein now contains links to allow interactive three-dimensional images to be displayed using Java programs such as KiNG [9], WebMol [10], or Jmol [11].

Recent advances in programmable graphics cards offer a number of new opportunities for illustrating proteins. Many inexpensive, commonly available graphics cards now fully support the use of the OpenGL Shading Language (GLSL [12]), which is used to write small programs, known as shaders, for modifying the graphics pipeline to produce sophisticated visual effects [13]. A few free molecular visualization tools have begun to take advantage of these new opportunities. The Visual Molecular Dynamics program uses GLSL to improve image quality and rendering speed [14,15], while QuteMol goes further by using GLSL to add illustrative rendering effects (also known as non-photorealistic rendering) such as borders around atoms and halo effects that make space filling, balls and sticks, and liquorice models much easier to interpret [16,17].

The ProteinShader program described in this paper further exploits GLSL by using custom texture mapping and lighting calculations implemented on the graphics card to produce ribbon and tube cartoon-style illustrative renderings of proteins that approximate what an artist might create by hand using pen and ink. Custom shading calculations are also used to map text labels and decorative textures onto the curved surfaces of tubes and ribbons shown in color.

## Implementation

ProteinShader is written primarily in Java, which was chosen because of its platform independence, as well as the ability of a Java Swing-based GUI to adopt the look and feel of the current operating system [18]. The Javadoc tool [19] was used to extract comments from the source code and generate the API (Application Programming Interface) files that are included in the help directory of the ProteinShader distribution. The current version of ProteinShader, beta 0.9.4, is available as Additional files 1 and 2, or can be downloaded from SourceForge [20], where future versions will be posted.

To obtain hardware-accelerated rendering of high quality three-dimensional perspective images of a protein, the low-level Open Graphics Library (OpenGL [21,22]) that runs on most modern graphics cards is used. The ribbons and tubes used by ProteinShader are drawn as collections of flat polygons tiled together to form continuous surfaces, and texture mapping coordinates are assigned to individual vertices as they are generated. Because OpenGL is primarily intended to work with the C/C++ language, Java Bindings for OpenGL (JOGL [23,24]) is used to allow the Java code to access OpenGL.

To map textures onto the surfaces of ribbons and tubes, vertex and fragment shaders written in the OpenGL Shading Language [12,13] are used. The vertex shader allows a programmer to manipulate directional vectors associated with a vertex, while the fragment shader is for applying custom equations for setting the color of each surface fragment (potential future pixel) [13,25].

To speed up the number of frames per second that can be rendering during an animation (a constant rotation), geometry is cached on the graphics card by using OpenGL display lists [26]. When tested with an inexpensive good quality mid-range graphics card, the ATI Radeon X1600, caching geometry in advance resulted in a nine-fold increase in performance for ribbons and a thirteen-fold increase in performance for tubes (data not shown).

## Results and discussion

### Overview of the ProteinShader GUI

**Figure 1:**
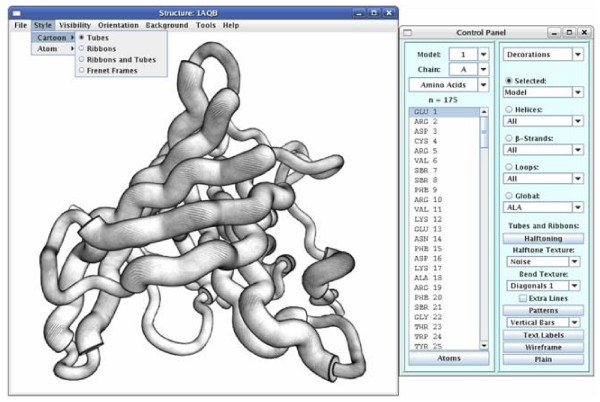
**The ProteinShader GUI**. A screen capture of the ProteinShader GUI with a pen-and-ink style rendering of the retinol-binding protein [PDB:1AQB], a β-barrel structure [27], as a tubes style display. In the control panel on the right, the entire model was selected in the Decorations subpanel, and real-time halftoning was applied by selecting Noise from the Halftone Texture menu and Diagonals 1 from the Bend Texture menu.

A screenshot of the ProteinShader GUI is shown in Figure 1, where the retinol-binding protein [27] is displayed as a pen-and-ink style rendering. The main window consists of a drawing canvas with a menu bar across the top, and the purpose of each menu is summarized in Table 1. The File menu's chooser box will open to the ProteinShader's data directory by default, so that is the best place to store protein structure files downloaded from the PDB web site [1].

**Table 1:** Menu bar options.

**Menu**	**Purpose**
File	In addition to Open and Quit, there is an Export Image submenu that allows the image on the canvas to be saved as a PNG or JPEG file.

Style	The Cartoon submenu allows the protein to be displayed as Tubes, Ribbons, Tubes and Ribbons, or Frenet Frames. The Atom submenu offers Space Filling (spheres), Balls and Sticks (spheres and cylinders), or Sticks (cylinders).

Visibility	Coarse-level of control over whether Amino Acids, Heterogens, or Waters are visible. Control panels in Table 2 provide a more fine-grained control.

Orientation	Choosing Original resets the protein to its original size and front orientation, while Front, Back, left, Right, Top, and Bottom affect the protein's orientation, but not the camera distance.

Background	Sets the background color on the canvas to Black, Gray, Light Gray, White, or opens a Chooser dialog box that can be used to select any color.

Tools	Opens and closes the control panel on the right side of the canvas.

Help	Opens the desktop's default web browser and loads a help html file.

The retractable control panel on the right side of the canvas is composed of two parts: a left-side subpanel that allows the user to select any model, chain, residue, or atom of the protein structure, and a right-side subpanel that can be switched to any of several different modifier or action subpanels. The menu at the top right of the control panel is used for changing the right-side subpanel, and the purpose of each subpanel is summarized in Table 2. Most of the modifier subpanels also allow selection of individual α-helices, β-strands, or loop regions.

**Table 2:** Subpanels of the retractable right-side control panel.

**Subpanel**	**Purpose**
Selection	The left-side subpanel allows selection of any model, chain, residue, water, heterogen, or atom of the protein. This subpanel is always present, whereas the right-side subpanel can be changed to any of the subpanels listed below.

Decorations	Applies texture maps and other special effects to ribbon and tube segments (the term segment refers to the length of a tube or ribbon that corresponds to an individual amino acid). This subpanel is visible in Figure 1.

Cartoon Color	Sets the color of tube and ribbon segments. Default colors based on region or amino acid type can be used, or a color chooser dialog box can be opened.

Cartoon Visibility	Sets any segment or group of segments in a tube or ribbon to be opaque, translucent, or invisible.

Cartoon Side Chains	Used to display amino acid side chains in combination with tubes or ribbons. The side chains can be shown as space filling, balls and sticks, or sticks.

Motion	Allows constant motion about the x-axis and/or y-axis of the protein. The frames per second will be displayed in the upper left of the canvas.

Antialiasing	Provides options for smoothing out the aliasing (jagged diagonal line) effect that often occurs at the edges of geometrically defined objects.

Atom Color	Sets the color of atoms in a space filling, balls and sticks, or sticks style display. Default colors based on atom or amino acid type can be applied, or a color chooser dialog box can be opened.

Atom Visibility	Sets any atom or group of atoms to be opaque, translucent, or invisible. A slider allows any degree of translucency from 0 to 100%.

Atom Scale	Adjusts the radii of spheres in a space filling or balls and sticks style display.

Tiling	Can be used to turn off automatic tiling (level of detail control) for spheres and cylinders. This panel has no effect on ribbon and tube style displays.

**Figure 2:**
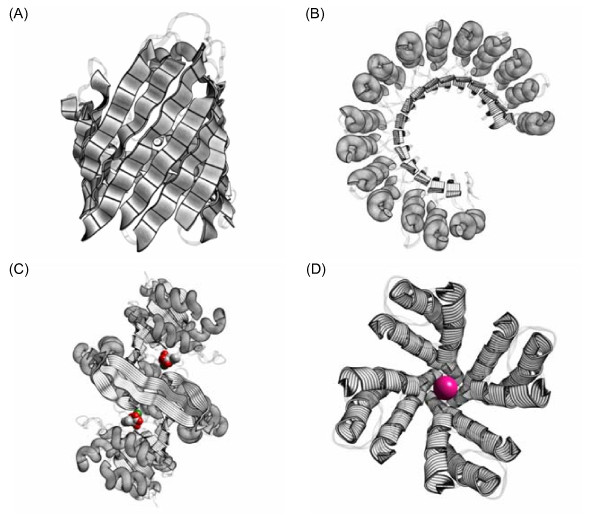
**Illustrative renderings of proteins**. Real-time halftoning and edge-line generation algorithms have been applied to ribbon and tube style displays generated from PDB structure files. (A) The porin protein [PDB:3POR], a transmembrane β-barrel structure [28,29]. (B) The ribonuclease inhibitor protein [PDB:2BNH], a α/β horseshoe-shaped structure [30]. (C) The 3-isopropylmalate dehydrogenase enzyme [PDB:1A05], a three-layer (α-β-α) sandwich-structure [31], with two substrate molecules (C, gray; O, red; Mg^2+^, green). (D) The potassium channel [PDB:1BL8], a transmembrane α-domain structure [32], with a K+ ion (deep pink) in the channel. In all four images, loop regions are de-emphasized by showing them as thin ribbons with 75% translucency. In (A) to (C), α-helices are shown as tubes, while β-strands are shown as wide ribbons. In (D), the α-helices are shown as wide ribbons.

A few examples of the kind of artwork ProteinShader can generate are shown in Figure 2 using the porin protein [28,29], the ribonuclease inhibitor protein [30], the 3-isopropylmalate dehydrogenase enzyme [31], and the potassium channel [32]. When a protein structure is loaded, the canvas automatically displays it as a pen-and-ink style illustrative rendering of ribbons and tubes, and the right side of the control panel is set to the Decorations subpanel shown in Figure 1. A variety of patterns can be applied to the ribbons and tubes by using the Halftone Texture and Bend Texture menus of the Decorations subpanel. The patterns are read from image files, which will be discussed further below in the section on texture mapping. A Cartoon Visibility subpanel can be used to deemphasize parts of the structure by setting them to be translucent, and the Visibility menu above the canvas can be used to display heterogens, such as the 3-isopropylmalate substrate molecules in Figure 2C or the K+ ion in Figure 2D.

In addition to the ribbons and tubes cartoon-type representations, the Style menu above the canvas can also be used to select atom-type representations: space filling (spheres), balls and sticks (spheres and cylinders), and sticks (cylinders). Dragging the mouse across the canvas can be used to rotate or zoom in on images, or an image can be rotated at constant speed by using a motion control panel. Details on mouse movements or the various control panels and menus can be found by using the Help menu above the canvas.

### General strategy for tubes and ribbons

Three-dimensional ribbons and tubes can be drawn by sweeping a waist polygon along a curved line at regular intervals and, at each point along the curve, aligning the polygon to a local coordinate frame (an xyz-axis system) that keeps the plane of the polygon perpendicular to the tangent of the curve [33]. When two copies of the polygon are placed at adjacent points along the curve, connecting their vertices can be used to define the small, flat polygons that ultimately approximate the curved surface of the ribbon or tube. In ProteinShader, the curved line and local coordinate frames needed for creating ribbons and tubes are generated using the xyz-coordinates of the α-carbons in each polypeptide chain.

### Local coordinate frames for α-carbons

**Figure 3:**
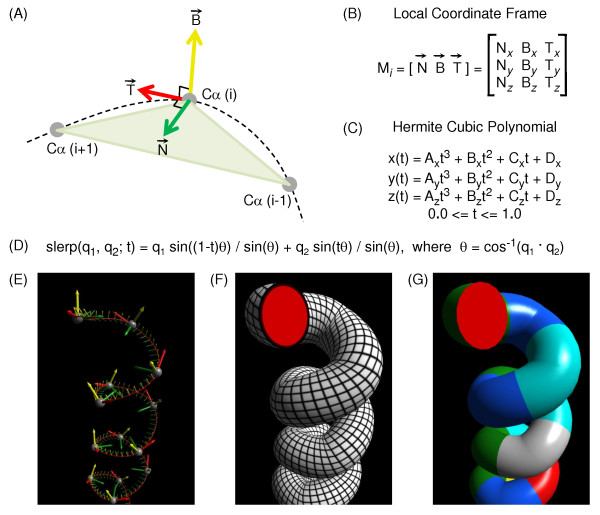
**Calculation of local coordinate frames and a spline for drawing tubes**. (A) The local coordinate frame for α-carbon number *i *in a polypeptide chain, C_α _(i), is calculated relative to the triangle (light green) that it forms with C_α _(i-1) and C_α _(i+1). The vectors N (Normal; green), B (Binormal; yellow), and T (Tangent; red) are the xyz-axes, respectively, of the local frame (see Local coordinate frames in the text). (B) The column vectors N, B, and T form a matrix that defines a rotation about C_α _(i). (C) The tangent vectors and xyz-coordinates of two α-carbons are sufficient to define a parameterized cubic polynomial equation (see Hermite interpolation in the text). The set of cubic polynomials connecting the α-carbons of a polypeptide chain form a spline, which is shown as a curved dotted line in (A). (D) The equation for interpolation between rotations using quaternions (see SLERP in text). (E) An α-helix after selecting Frenet Frames from the Style menu. The α-carbons (small gray spheres) have local frames represented by green, yellow, and red vectors as in (A). The smaller local frames between α-carbons are interpolated by using the Hermite-SLERP algorithm described in the text. (F) The α-helix from (E) after selecting Tubes from the Style menu and Wireframe from the Decorations panel. To draw the tube, a regular polygon defined by 20 vertices in a circle (red) is swept along the spline and rotated into alignment with the xy-plane of each local frame. Connecting vertices between successive positions of the polygon produces the surface of the tube. (G) The α-helix from (F) after Plain is selected from the Decorations menu and the Cartoon Color subpanel is used to color tube segments by amino acid type.

To define each α-carbon's local xyz-coordinate frame, the technique illustrated in Figure 3A is used. For α-carbon *i*, a tangent vector T (z-axis) is calculated as the vector pointing from α-carbon (*i *- 1) to α-carbon (*i *+ 1). A second vector in the same plane is calculated by subtracting α-carbon (*i *- 1) from α-carbon (*i*), and a binormal vector B (y-axis) is then calculated as the cross product of this second vector and T. Finally, the cross product of B and T is used to obtain a normal vector N (x-axis). If a previous or next α-carbon is missing, the calculations use the current α-carbons's amino group nitrogen or carbonyl group oxygen, respectively.

N, B, and T form a right-handed perpendicular xyz-axis system, with N and T in the plane represented by the light green triangle in Figure 3A. Written as column vectors, N, B, and T form the rotation matrix shown in Figure 3B. This matrix can be used to take a waist polygon drawn in the xy-plane of a global xyz-coordinate system and rotate it into the xy-plane of the local coordinate frame for an α-carbon.

### Hermite interpolation

To develop a curved line that passes through the α-carbons in a chain, Hermite interpolation [34] is used. The curved line is actually a spline, a series of piecewise cubic polynomial equations, where each polynomial equation begins at one α-carbon and ends at the next. The xyz-coordinates and tangent vectors of the two α-carbons are used to solve the constants A, B, C, and D in the set of parameterized equations shown in Figure 3C. The parameter *t *is set to 0.0 at α-carbon (*i*) and to 1.0 at α-carbon (*i *+ 1), so intermediate values of *t *can be used to solve for the xyz-coordinates of any point on the curved line. The tangent vectors used in the calculations are adjusted to a length of 4.0 because that gives a reasonable curvature for α-helices and β-strands.

### SLERP

An algorithm is also needed for interpolating between the local coordinate frames of α-carbons. The tangent (z-axis) of each interpolated frame could be calculated from the first derivatives of the equations shown in Figure 3C, and a simplistic linear interpolation could be used to calculate a normal (x-axis) and binormal (y-axis) for each point. However, a much smoother interpolation can be achieved by using the spherical linear interpolation (SLERP) parameterized equation shown in Figure 3D[35-37].

SLERP, which is based on the use of quaternions, is commonly used in computer graphics for gliding a camera through a scene because it avoids the quirks and jerky motion of earlier methods [37]. A quaternion is a four-tuple devised by W. R. Hamilton to extend complex numbers into multiple dimensions, but it can also be used to represent a three-dimensional rotation in space [35,37]. A rotation matrix can be converted into a quaternion [38], and interpolating between quaternions produces a smoother rotation than attempting to interpolate between matrices.

### Hermite-SLERP algorithm

To maintain the three-dimensional structure of a tube or ribbon, the waist polygon drawn in the xy-plane of each local coordinate frame should be kept perpendicular to the spline, so the tangent (z-axis) of each local frame should closely matches the tangent of the spline. A minor problem with using SLERP is that the tangent of each interpolated quaternion (the z-axis of the local frame that the quaternion is equivalent to) will not necessarily match the tangent calculated by Hermite interpolation.

To fix any discrepancy between the SLERP- and Hermite-calculated tangents, the tangents are compared, and if there is more than one degree of difference, a rotation is used to make the SLERP tangent match the Hermite tangent. The axis and angle of rotation are calculated using the cross product and dot product, respectively, of the two tangents, and for convenience the axis and angle are converted into a quaternion.

Multiplying the interpolated quaternion by the tangent-fix-up quaternion adjusts the interpolated quaternion so that if it was converted back into a rotation matrix, its tangent would now match the tangent of the spline. The net effect of these manipulations is that the tangent (z-axis) of each local frame along the spline is determined by Hermite interpolation, while the SLERP algorithm provides for a smooth, gradual rotation of the xy-axes.

### Frenet Frames

To visualize the spline and local coordinate frames produced by the Hermite-SLERP algorithm, the Style menu above the canvas has a Frenet Frames option. An α-helix from the c-Jun protein [39] is shown as Frenet Frames in Figure 3E, where the local frames use the same color scheme as in Figure 3A, and the interpolated frames are shown on a smaller scale. In Figure 3F, the same α-helix is shown after selecting Tubes from the Style menu and Wireframe from the Decorations panel. The red end cap is the waist polygon that is swept along the spline while drawing the tube, and the lines of the wireframe connect the vertices that define the surface of the tube. In Figure 3G, the α-helix is shown after selecting Plain from the Decorations panel and using the Cartoon Color panel to apply colors based on amino acid type. Phong lighting calculations [40,41] are used to smooth out the appearance of the tube's surface and to add specular highlighting (the shiny plastic-like appearance) to enhance the three-dimensional quality of the image.

### Untwisting β-strands

**Figure 4:**
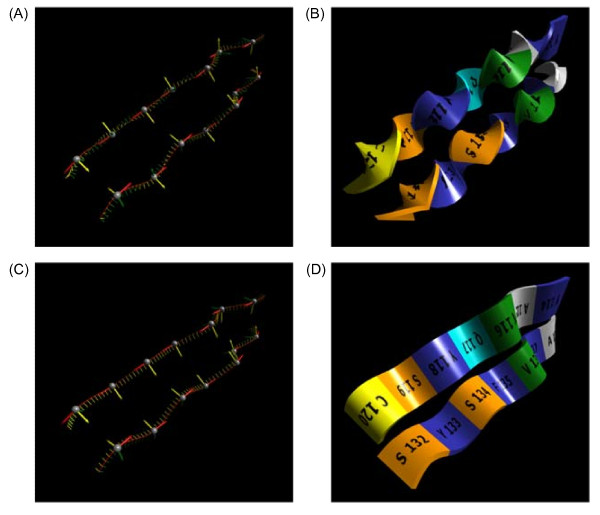
**Untwisting β-strand ribbons**. (A) The local coordinate frames for two β-strands are calculated using the same algorithm as for the α-helix in Figure 3. In this view, the direction of the y-axis (yellow) of the local frames can be seen to alternate by approximately 180 degrees for each successive α-carbon in a chain. (B) The ribbons drawn using the local coordinates frames shown in (A) have a highly twisted appearance, making the text labels texture mapped onto their surface very difficult to read. (C) The local frames for the β-strands in (A) are shown after being adjusted by an algorithm that compares the local frames for successive α-carbons and, if necessary, rotates frames 180 degrees about their z-axis. (D) The ribbons drawn using the local coordinate frames shown in (C) have a smoothed out appearance. The direction of the text labels indicates the amino to carboxyl direction of the polypeptide chain.

The Hermite-SLERP algorithm works fine for α-helices and loops, but encounters a problem with β-strands, where the amino acid side chain directionality alternates by approximately 180 degrees for successive residues. The local coordinate frames will, in most cases, flip direction for every other α-carbon. Consequently, a β-strand ribbon will appear highly twisted as illustrated in Figure 4, where two β-strands are shown as Frenet frames (Figure 4A) or ribbons (Figure 4B). The twisted ribbons are visually difficult to follow, and any images mapped onto their surface become highly distorted.

To fix this problem, the local coordinate frames for successive α-carbons are compared to check for a radical change in direction. The frames are aligned along their tangents (z-axes) by using the procedure described earlier for making SLERP-calculated tangents match Hermite-calculated tangents, and if the angle between the two binormal vectors (y-axes) is greater than 90 degrees, the second frame is rotated 180 degrees about its tangent (z-axis). After performing this fix-up step along a β-strand's length, the frames will be aligned as shown in Figure 4C. The appearance of the ribbon is greatly improved as shown in Figure 4D, and the antiparallel nature of the two β-strands becomes more obvious because the crests and valleys of the ribbons coincide.

It might seem simpler to assume that the coordinate frame for every second α-carbon should be rotated. However, that strategy will not always work because there are occasional irregularities in the structure of lengthy β-strands, as well as some problems with how β-strands are defined.

### Combining side chains with ribbons

**Figure 5:**
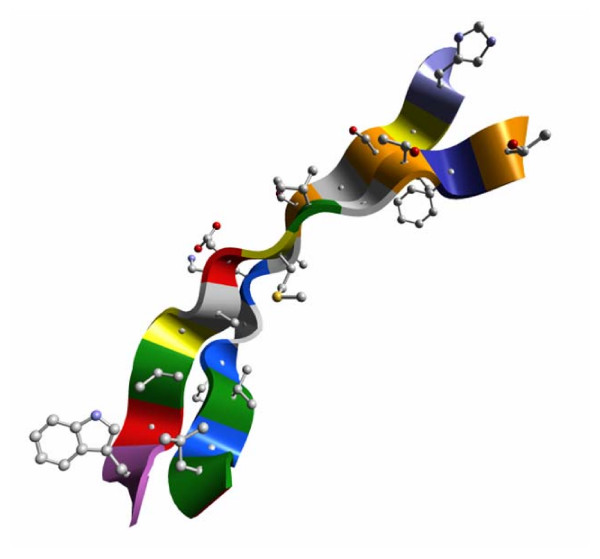
**Combining balls and sticks style side chains with ribbons**. β-Strands 3 (H52-L63) and 4 (W67-T78) of the retinol-binding protein [PDB:1AQB] are shown as ribbons (main chain) with side chains added as a balls and sticks style display. The small gray spheres that appear to be imbedded in the ribbon are the α-carbons of the main chain.

Because the spline runs through the α-carbons, when balls and sticks representations of amino acid side chains are combined with ribbons, the side chains appear to be firmly attached to the ribbon. This effect is illustrated in Figure 5, which shows two β-strand ribbons after the Balls and Sticks button was clicked in the Cartoon Side Chain subpanel of the Control Panel. By looking closely along the length of the ribbon, the alternating orientation of side chains discussed in the previous section can be clearly observed.

### Segments and texture mapping

If a region of secondary structure were stored as a single collection of vertices, dynamically applying colors or textures to mark individual amino acids would become quite complex. Therefore, the basic geometric unit of organization for rendering tubes and ribbons in the ProteinShader program is a segment, which is defined as a length of a tube or ribbon that corresponds to a single amino acid. A segment's center is the xyz-coordinates of its α-carbon, while its beginning and end are the midpoints along the spline to the previous and next α-carbon, respectively.

**Figure 6:**
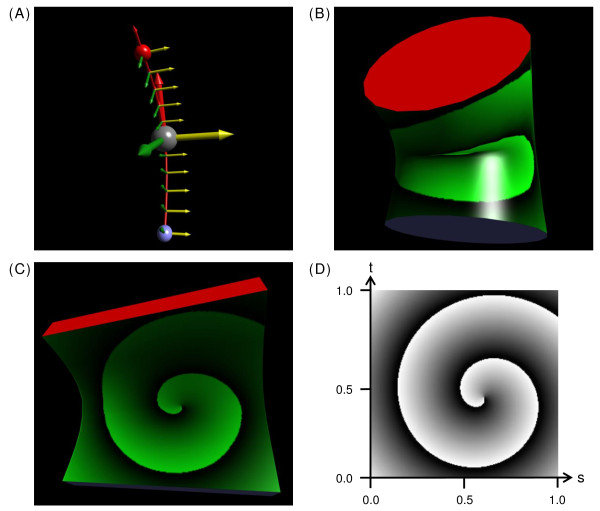
**Texture mapping onto the curved surfaces of tube and ribbon segments**. (A) The local coordinate frames needed for drawing a single amino acid as a tube or ribbon segment are shown using the same conventions as in Figure 3, except that a small blue sphere marks the amino-terminal end of the segment, while a small red sphere marks the carboxyl-terminal end. The same amino acid is drawn as a tube segment in (B) and as a ribbon segment in (C). In both (B) and (C), a blue end cap indicates the amino-terminus of the segment, while a red end cap indicates the carboxyl-terminus. (D) This two-dimensional swirl image was mapped onto the curved three-dimensional surfaces of the tube (B) and ribbon (C) segments by using (s, t) texture coordinates that are assigned to each vertex of a segment when its geometry is first calculated.

A segment can be thought of as a collection of local coordinate frames, as shown in Figure 6A, where an amino acid is shown as Frenet frames with blue, gray, and red spheres marking the beginning, α-carbon, and end of the segment, respectively. The same amino acid is also shown as a tube segment (Figure 6B) and as a ribbon segment (Figure 6C). As the waist polygon that specifies the vertices of a tube or ribbon segment is swept along the spline and aligned to each local frame, every vertex is assigned a surface normal and a pair of texture coordinates. The surface normal is a vector needed for lighting calculations, while the texture coordinates allow two-dimensional images such as the swirl pattern in Figure 6D to be systematically mapped onto the surface of a tube or ribbon segment. By convention, the texture coordinates are referred to as *s *and *t*, and each coordinate is on a scale from 0.0 to 1.0 [42,43].

Vertices at the beginning of a segment are assigned a *t*-coordinate of 0.0, while vertices at the end are assigned a *t*-coordinate of 1.0. The *s*-coordinate, on the other hand, increases in the counter clockwise direction as the vertices of the waist polygon are drawn in the xy-plane, and the exact start and end values are somewhat variable. For example, the broad surfaces of ribbons have *s*-coordinates from 0.0 to 1.0, but on the narrow sides of ribbons the s-coordinates run from 0.0 to only 0.125. For tubes, the s-coordinate runs from 0.0 to 2.0 so that the same texture map will be wrapped around the tube twice.

The Patterns, Text Labels, and Wireframe buttons of the Decorations subpanel accomplish their effects by using texture mapping. The images in the Patterns menu are read from PNG (Portable Network Graphics [44]) or JPEG (Joint Photographic Experts Group [45]) files in the textures/patterns subdirectory, and the menu can be modified by editing a configuration file. The lines for the wireframe images are calculated on-the-fly, while the images needed for amino acid labels are generated whenever a protein structure is first loaded by extracting letters and digits from an image file. Texture mapping is also important for creating pen-and-ink style drawings.

### Pen-and-ink style drawings

To produce pen-and-ink style drawings, a variety of edge lines need to be added. For ribbons, many of the edge lines can be added by darkening fragments of a surface if they have an *s *or *t *coordinate close to the minimum or maximum. For tubes, however, this approach is only useful at the beginning or end of a segment, as what appears to be an edge along the length of a tube is determined by the view angle.

**Figure 7:**
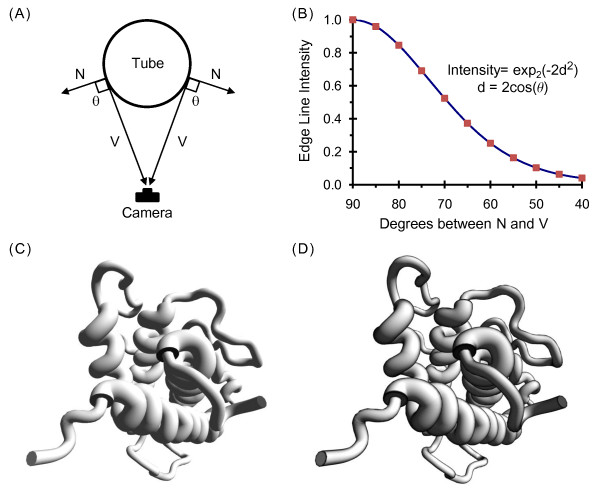
**Generation of edge lines for pen-and-ink style drawings**. (A) Edge lines for a tube are calculated by measuring the angle between the view vector V and the surface normal vector N, and then darkening a surface if the angle is close to 90 degrees. (B) The equation for edge-line intensity is based on the smooth function used for single-pass wireframe rendering [46]. (C) A tube style display of the human growth hormone protein [PDB:1HGU], a four α-helix bundle structure [47], is shown in gray scale and using Phong lighting with a single directional light and no specular highlighting. (D) Edge lines are added to the image shown in (C).

A solution for generating edge lines based on the view angle is presented in Figure 7. Lighting calculations typically use a surface normal that indicates the direction a fragment faces, a view vector from the fragment to the camera, and a lighting vector from the fragment to the light source (a fragment is similar to a pixel, but occurs earlier in the graphics pipeline). As shown in Figure 7A, if the angle between the surface normal (N) and the view vector (V) is close to 90 degrees, then the surface is an edge and should be darkened.

Instead of a sharp cutoff, the smoothing function graphed in Figure 7B is used to determine edge line intensity: *I *= exp_2_(-2*d*^2^), where *d *= 2cosine(θ) and θ is the angle between N and V. This equation is from a paper on single-pass wireframe rendering [46], where *d *was a distance. The equation is also used to smooth edge lines generated from texture coordinates.

The result of these edge-line calculations can be seen by comparing two images of the human growth hormone protein [47]. In Figure 7C, a color model has been converted to grayscale by using the equation: *gray = 0.30 red + 0.59 green + 0.12 blue*. In Figure 7D, edge lines generated from the view angle have been applied along with texture-coordinate based edges added to segment end caps.

**Figure 8:**
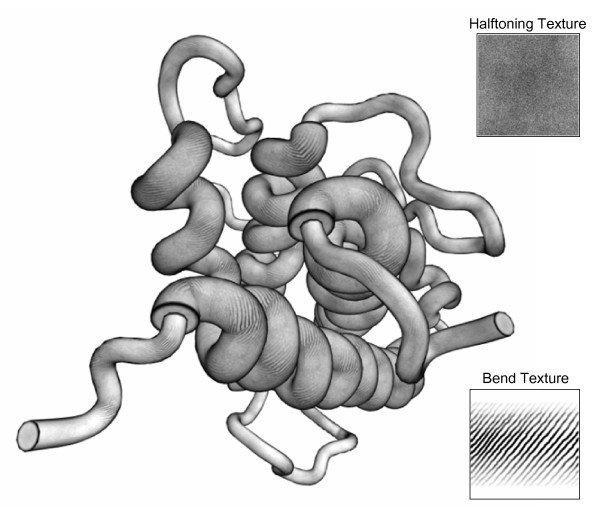
**Use of real-time halftoning and bend textures for pen-and-ink style illustrative renderings**. The human growth hormone protein image shown in Figure 7D is shown with a halftoning texture (upper right inset) and a bend texture (lower right inset) mapped onto the surface of each tube segment. The halftoning texture is mixed with lighting calculations, whereas the bend texture is multiplied by a bend factor from 0.0 to 1.0 that is determined by comparing the tangents at the very beginning and end of a segment.

The image in Figure 7D is shown in Figure 8 after using the real-time halftoning technique [48] to mix a noise texture (upper right inset) with grayscale lighting calculations by using the smooth threshold function, *color *= *1.0 *- aliasFactor*(1.0 - {halftoneColor + grayscaleColor} [49]. An aliasFactor of 4.0 is suitable for some applications of halftoning [49], but a number closer to 1.0 is used in ProteinShader to obtain a more subtle effect. This function allows for grayscale values, whereas some versions of halftoning only allow black and white.

To emphasize bends in the middle of segments, a second texture has been applied to the image in Figure 8 (see lower right inset), and the intensity of the texture is proportional to how strongly a segment's spline is bent. A bend factor on a linear scale from 0.0 to 1.0 is calculated by comparing the tangents at the beginning and end of a segment. If the tangents have an angle close to 180 degrees when placed tail to tail, the spline is nearly straight and the bend factor is close to 0.0. If the angle is almost 50 degrees, the segment is strongly bent, and the bend factor is close to 1.0.

Halftoning and bend textures can be selected from menus in the Decorations panel shown in Figure 1. The textures are stored as PNG [44] or JPEG [45] files, and the Help menu has directions for adding new textures. Textures can be assigned to individual segments of a ribbon or tube, so the pen-and-ink style drawing can be mixed with other options (Patterns, Text Labels, Wireframe, and Plain).

**Figure 9:**
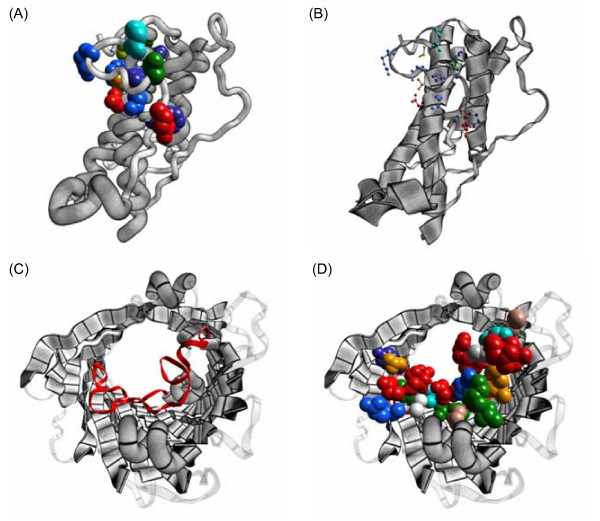
**Combining amino acid side chain displays with pen-and-ink style illustrative renderings**. (A) The human growth hormone image from Figure 8 is displayed in a different orientation, and amino acid side chains involved in binding to the growth hormone receptor [50] are shown as a space filling style display colored by amino acid type (the amino acids are F10, F54, E56, I58, R64, Q68, D171, K172, E174, T175, F176, R178, C182, and V185). The α-carbons are also shown. (B) The same as (A), except that the protein is shown as ribbons and the amino acid side chains are shown as a balls and sticks style display. (C) The transmembrane β-barrel protein porin that was shown as a side view in Figure 2A is shown here with an end view of the β-barrel. A loop region (loop 7) that fits inside the β-barrel is highlighted in red. (D) The same as (C), except that the amino acid side chains of loop 7 are shown as a space filling style display and are colored by amino acid type. Loop 7 restricts the size of the channel to a narrow region called the eyelet, which is about 8 angstroms in diameter and 9 angstroms in length [28,29].

Selection of individual segments also allows important regions of a protein to be highlighted by adding amino acid side chains to pen-and-ink style drawings. As examples, in Figure 9 side chains involved in binding of human growth hormone to its receptor [50] have been added as a space filling (Figure 9A) or balls and sticks (Figure 9B) style display, and the way that a loop region (red in Figure 9C) of the transmembrane β-barrel porin protein fills up much of the channel [28,29] is illustrated with space filling side chains (Figure 9D).

### Performance costs for shaders

**Figure 10:**
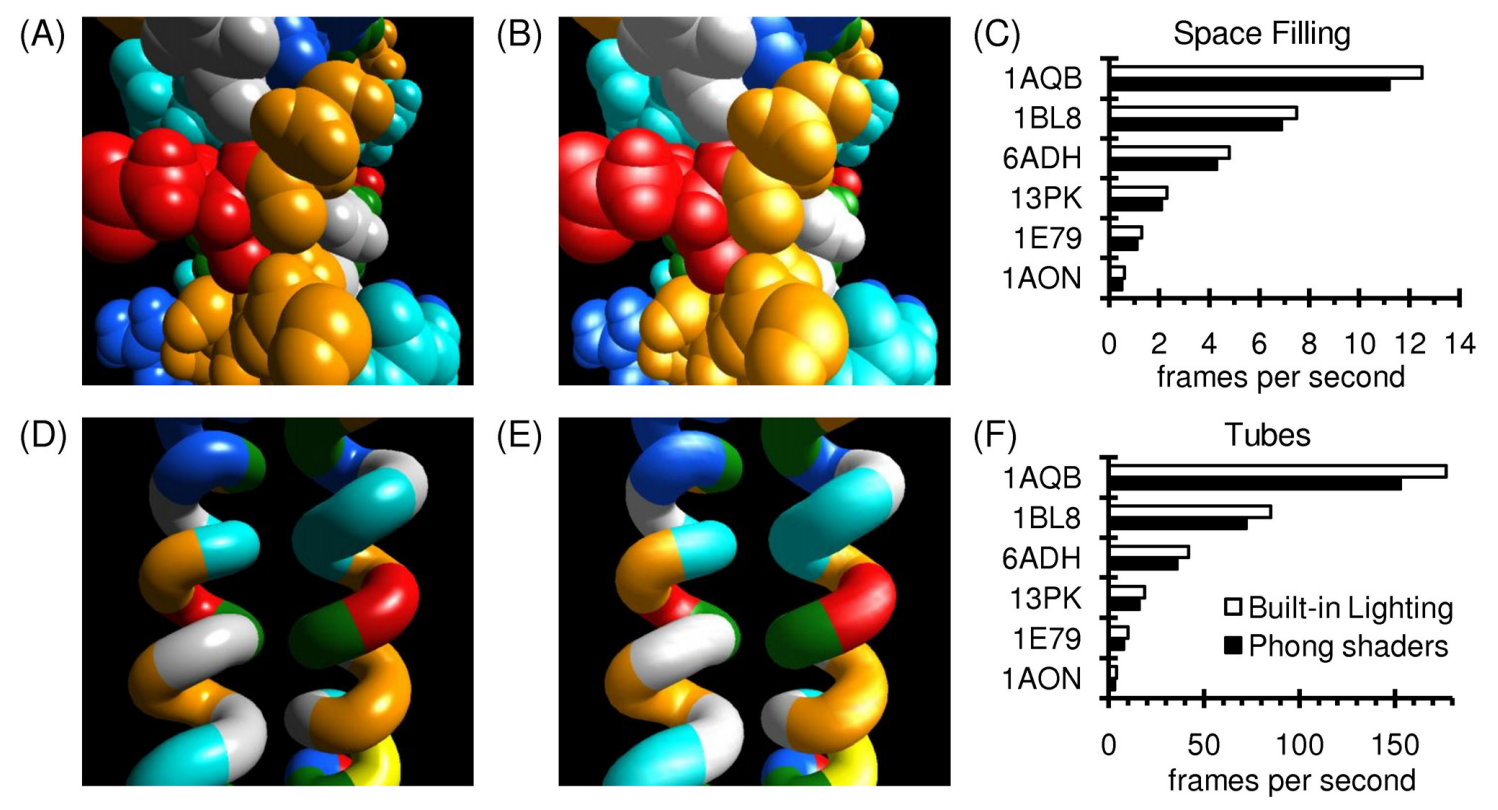
**Comparison of Phong vertex and fragment shaders to OpenGL's built-in lighting**. (A) A close up view of a small portion of the c-Jun homodimer [PDB:1JUN], a leucine zipper protein [39], is shown as a space filling (spheres) style display. Lighting calculations were performed using the phong.vert and phong.frag shaders in the ProteinShader shaders directory. (B) The same view as in (A), except that OpenGL's built-in lighting was activated by temporarily removing the phong.frag file from the shaders directory and restarting the program (a dialog box warns the user that the Phong shaders could not be compiled, and OpenGL's built-in lighting is used as a backup). (C) Several proteins were used to compare the frames per second that could be rendered during an animation of a space filling style display while using OpenGL's built-in lighting (white bars) or Phong shaders (black bars). (D) Phong lighting is used on a tubes style display of the same protein as in (A). (E) The same view as (D), except that OpenGL's built-in lighting is used. (F) The same comparisons as in (C), except that a tubes style display is used. See Table 3 for the sizes of the test proteins and notes on the computer used for performance testing.

**Figure 11:**
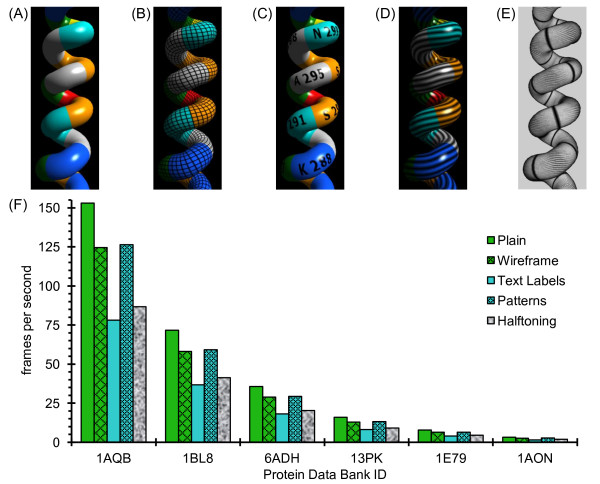
**Performance costs of texture mapping, edge line generation, and real-time halftoning**. An α-helix is shown after using the Decorations subpanel of the ProteinShader GUI to select Plain (A), Wireframe (B), Text Labels (C), Patterns (D), or Halftoning (E). (F) Several proteins were used to compare frames per second during an animation for the type of images shown in (A) through (E). The items in the graph legend are presented in the same order as (A) through (E). See Table 3 for the sizes of the test proteins and notes on the computer used for performance testing.

The experiments in Figures 10 and 11 measure the performance costs for using vertex and fragment shaders to perform custom lighting, texture mapping, and edge-line generation calculations. For a space filling style display, Phong lighting using custom shaders (Figure 10A) produces a smoother, higher quality image than using the built-in OpenGL lighting (Figure 10B). The performance cost appears to be fairly minor (Figure 10C), with about a 12% reduction in frames per second during an animation when the Phong shaders are used rather than the built-in lighting. Similar results were seen for a tubes style display (Figures 10D to 10F), with about a 17% reduction in frames per second.

**Table 3:** Proteins used in performance testing.

**PDB ID**	**Residues**	**Atoms**	**Protein**
1AQB	175	1574	retinol-binding protein
1BL8	388	2824	potassium channel
6ADH	748	5669	alcohol dehydrogenase
13PK	1660	12508	phosphoglycerate kinase
1E79	3315	25248	F1 ATPase inhibited by DCCD
1AON	8015	59674	GroEL-GroES-(ADP)7 chaperonin complex

**Figure 12:**
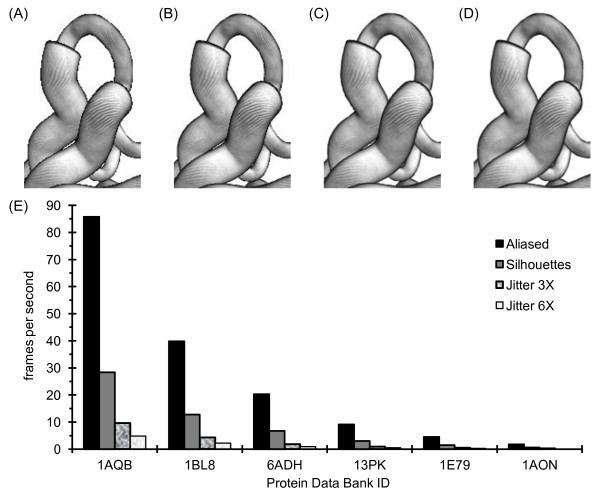
**Antialiasing object edges**. (A) The dark edges of a small portion of the retinol-binding protein from Figure 1 display a marked alias (jagged edge) effect if nothing special is done to smooth out the edges. (B) Same as A, except that the dark edges have been partially smoothed by using the Antialiasing panel of the ProteinShader GUI to select an option to antialias the black edges of halftoning images by using translucent black silhouettes to add gray pixels to the edges (see Antialiasing section of text). (C) Same as (B), except that the Antialiasing panel has been used to select an option to perform additional antialiasing by jittering the entire scene 3 times and blending the images. Each jittered image is offset by a fraction of a pixel from the original image. (D) Same as (C), except that the scene is jittered 6 times. (E) The performance costs for antialiasing are measured by comparing frames per second during a constant rotation. See Table 3 for the sizes of the test proteins and notes on the computer used for performance testing. The items in the graph legend are presented in the same order as (A) through (D). The images were generated on a monitor with a 72 pixels per inch resolution, where the alias effect in (A) is quite obvious. Because of the dramatic slowdown in rendering time, antialiasing is primarily intended for saving static images, not for animations.

The performance tests graphed in Figures 10 to 12 were made on a Macintosh 2.16 GHz Intel Core 2 Duo with 2 GB of RAM and an ATI Radeon X1600 graphics card with 256 MB VRAM. To prevent an out of memory error for the largest protein tested, the chaperonin complex, the maximum size of the Java heap was increased to 512 MB by adding "-Xmx512m" as an argument to the java command in the run.sh script provided with the ProteinShader distribution.

For Figure 11, the Decorations subpanel of the ProteinShader GUI was used to select shaders for special effects while rendering a tubes style display. For each test protein in Figure 11F, if the Phong lighting (Plain) frames per second is considered to be 100%, switching to the wireframe shaders or adding patterns by texture mapping typically results in a fairly minor reduction of less than 20%, while more complex calculations such as adding text labels or halftoning result in reductions of almost 50%. Given the quality of the images obtained, the performance costs for using custom vertex and fragment shaders seems reasonable. A caveat to these results, however, is that a fairly recent good quality graphics card is required.

The proteins tested in Figure 11F range from 175 amino acids to about 8,000 amino acids (see Table 3), and each approximate doubling in protein size results in a roughly 2-fold reduction in frames per second. Overall, the 46-fold increase in number of residues from the smallest to the largest test protein (1AQB to 1AON) results in very close to a 46-fold decrease in frames per second (from 153 to 3.2 frames per second for Phong lighting (Plain) and from 86.7 to 1.9 frames per second for Halftoning). These results indicate that, at least within this size range, rendering times scale in a nearly linear manner for tubes style displays with custom shaders.

### Antialiasing

When pen-and-ink style ribbons and tubes are drawn on a white background, the darkened edges often appear to be quite jagged, as shown in Figure 12A. This phenomena, which is referred to as aliasing [51,52], occurs because a pixel is determined to be all the way in or out of an object based on whether the center of the pixel falls within a boundary line.

The ProteinShader control panel has an Antialiasing subpanel, where the option to antialias black edges of halftoning images can be selected. By adding gray pixels outside object boundaries, this option partially smooths out the jagged edges, as shown in Figure 12B. To accomplish this effect, a translucent black silhouette of each tube or ribbon segment is rendered four times with a slight offset (a half pixel up, down, left, or right) before rendering the segment with halftoning.

The Antialiasing subpanel also provides an option for smoothing any image by rendering the entire scene several times with jitter and blending the images. The scene can be jittered from 2 to 16 times using jitter values taken from the OpenGL Programming Guide [53], and this antialiasing can be combined with the silhouette-based antialiasing to produce nicely smoothed dark edges, as shown in Figures 12C and 12D.

Antialiasing dramatically slows rendering during an animation, as shown in Figure 12E. The silhouette-based antialiasing slows rendering by about 3-fold, while jittering the entire scene n times will slow rendering down about n-fold. Because of the performance costs, antialiasing is intended mainly for saving static images as PNG or JPEG files. Antialiasing is used on all of the images in previous figures, except for Figures 10 and 11.

## Conclusion

The ProteinShader program is a platform-independent Java-OpenGL molecular visualization tool that exploits recent advances in programmable graphics cards. The primary accomplishment of this free, open-source code program is its ability to render a protein as a cartoon-style drawing that approximates what an artist might create by hand using pen and ink (see Figures 1, 2, 8, and 9). The artistic effects employed by ProteinShader rely heavily on texture mapping, where two-dimensional images are systematically mapped onto the curved surfaces of three-dimensional ribbons and tubes. To minimize distortions or irregularities in the images used as textures, a hybrid Hermite-SLERP algorithm was developed for generating smooth, gradually rotating tubes and ribbons.

The custom texture mapping and lighting calculations needed for rendering pen-and-ink style images are implemented using vertex and fragment shaders written in the OpenGL Shading Language [12,13], which is supported on most new graphics cards for ordinary desktop and laptop computers. Shaders are also used for mapping text labels and decorative textures onto the surfaces of ribbons and tubes shown in color.

To create images suitable for publication, the program has antialiasing options that can nicely smooth out the jagged (pixelated) edges that are often seen in computer-generated images. However, antialiasing dramatically slows rendering time, so it may not be suitable for animations, unless a fairly high-end graphics card is used. The performance costs for using custom shaders rather than OpenGL's built-in lighting equations appears to be fairly minor on recent graphics cards.

As an aid to future development, a Frenet frames style display allows the user to visualize the mathematics that underlies the tubes and ribbons. Key areas for future development are representations of DNA, which is not currently supported, and selection by clicking on parts of an image. In the present version of the ProteinShader program, all manipulations are done through user-friendly menus and control panels.

## Availability and requirements

• Project name: ProteinShader

• Project home page: 

• Operating system: Platform independent (tested on Linux, Macintosh OS X, and Windows XP)

• Programming languages: Java and OpenGL Shading Language

• Other requirements: Java 1.5 and a graphics card supporting OpenGL 2.0 or higher.

• License: GNU General Public License

• Restrictions to use by non-academics: None

## Authors' contributions

JRW wrote the program and authored the manuscript. All authors read and approved the final manuscript.

## Supplementary Material

Additional file 1 **ProteinShader program without source code.** This compressed file contains the complete ProteinShader program including associated libraries, but no source code. A README.txt file gives an overview of the ProteinShader distribution, and the index.html file in the help subdirectory has directions on getting started with the program as well as a set of tutorials.

Additional file 2 **ProteinShader program with source code.** This compressed file contains everything in the binary distribution plus the Java source code and a build.xml file for compiling with Ant. Click here for file
