# Supplementary material for: ProteinShader: illustrative rendering of macromolecules
Source: BMC Struct Biol. 2009 Mar 30;9:19. doi: 10.1186/1472-6807-9-19 (PMC2672931; doi:10.1186/1472-6807-9-19)
Supplement: Additional file 1 — ProteinShader program without source code. This compressed file contains the complete ProteinShader program including associated libraries, but no source code. A README.txt file gives an overview of the ProteinShader distribution, and the index.html file in the help subdirectory has directions on getting started with the program as well as a set of tutorials. [file 1472-6807-9-19-S1.zip › ProteinShader-beta-0_9_4-binary/licenses/glfont/glfont.htm]

Brad Fish's glFont Version 2.0


# Brad Fish's glFont Version 2.0

### glFont and glFont API

Copyright (c) 1998-2002 Brad Fish  
E-mail: bhf5@email.byu.edu  
Web:  http://students.cs.byu.edu/~bfish/  

### Table of Conents

1. Disclaimer
2. Introduction
3. New in glFont 2.0
4. System Requirements
5. Files You Should Have Received
6. Distribution
7. Terms of Use
8. Known Issues
9. Bugs, Comments, Suggestions,
   etc.
10. glFont 2.0 API
11. Appendix

### Disclaimer

Because this is the initial release of glFont 2.0, I am expecting the possibility
of errors in this document, the glFont builder, and the API.  I've done my
best to eliminate such errors, but you should be aware that this is not perfect
by any means.

### Introduction

Thanks for downloading the glFont 2.0!

glFont is a Win32 program that creates a texture containing a range of
characters in a specified font, and automatically generates texture coordinates
for use in OpenGL applications that need to display text.  The font type is
not limited to monospaced fonts; each character is displayed with correct
spacing and size.

Text is rendered by texture mapping specific characters onto OpenGL
quads.  It is quite possible and easy to modify the color, size, and
position of the text quads using standard OpenGL functions.  It is also
quite possible to load as many different fonts as you wish, and use them
whenever you please.

I first released glFont back in 1998, and was one of the first Windows
TrueType bitmap font generators.  It has been nearly four years since the
initial release of glFont, and I still receive e-mails about it on almost a
weekly basis.  It has helped hundreds of people find a fast, efficient, and
easy solution to outputting text in OpenGL programs.  It's always fun
stumbling across OpenGL projects on the internet and seeing glFont in action.

### New in glFont 2.0

I am pleased to release this new version of glFont.  It has been
something I've wanted to do for some time now.  Version 2.0 includes many
enhancements from version 1.0, including:

- Font antialiasing in the glFont builder
- Enhanced C++
  API
- C++ wide character support

**NOTE:** The files create by the glFont 1.0 builder and the glFont 1.0
API are not compatible with glFont 2.0.  The structure of the file remains
largely the same, but the dx and dy calculations are different and will render
your 1.0 API fonts and code unusable.  However, I don't see any reason why
anyone would want to continue using the old 1.0 API.  Please modify your
code to work with the 2.0 API (don't worry, it's easy).

### System Requirements

The glFont builder tool requires a Windows OS with Unicode capabilities and
GDI+.  The latest GDI+ runtime distributable can be found at this link: http://www.microsoft.com/msdownload/platformsdk/sdkupdate/psdkredist.htm. 
Just place the gdiplus.dll in the same directory as the glFont executable.

The glFont 2.0 API requires a competent C++ compiler, and uses the Standard
Template Library.  It has been tested with Microsoft Visual C++ version 6.0
and 7.0 (native Win32, none of that .NET stuff), and with GNU g++ 2.96 packaged
with the RedHat Linux 7.2 distribution.

### Files You Should Have Received

The following files should be contained in the ZIP you downloaded.  If
not, please contact me, and let me know where you downloaded it from.

glfont.exe -- The glFont builder  
glfont.exe.manifest -- Makes the glFont builder use cool new common controls
under Windows XP  
glfont2.h -- The glFont Version 2.0 API header  
glfont2.cpp -- The glFont Version 2.0 API implementation  
glfont.html -- Introduction, documentation, licensing and terms of use

### Distribution

You may distribute glFont as long as it is the original, unmodified ZIP,
available from my web site, containing the files above, unaltered.

If you wish to distribute a modified version of the glFont API, please get in
touch with me first so that I can post it at the official glFont website, the
reason being that others may find your modifications useful, and having them all
at the official site will help reduce confusion.

The newest version of glFont is always available at the following URL: 
http://students.cs.byu.edu/~bfish/glfont.php.

### Terms of Use

You may use the source to the glFont API in any program you wish, commercial
and non-commercial, as long as credit is given to myself (in the accompanying
program documentation, or in the program itself), including my e-mail address
and a link to my web page.  You may modify the API source to fit your
specific needs if you wish, but the same terms apply--you must provide original
credit to myself.

glFont has been a great help to many, many people.  I frequently receive
compliments and success stories from individuals all over the world who have
used glFont in their programs and found it to be an excellent solution. 
glFont 2.0 provides many enhancements, but just like the original glFont, I am
allowing it to be used without cost because I think it can benefit many in the
OpenGL community.  Through abiding by these terms and providing credit
where due, you help others learn about glFont and give them the same opportunity
to use it.

The source to the glFont builder is available for purchase; contact me if you
are interested.

### Known Issues

The glFont builder tool can only run on on a GDI+ enabled Windows operating
system.  This should include everything from Windows ME up, although you
may have to download the GDI+ runtime distributable.  See System Requirements.

The glFont builder tool only works with TrueType fonts.  If you select a
raster or bitmapped font, it won't display.  Fix: don't select a raster or
bitmapped font.  Why would you want to, anyway?

### Bugs, Comments, Suggestions, etc.

Please let me know immediately about
any bugs.  If you have comments or suggestions, please drop me a line as
well.  I am definitely anticipating that there will be some bugs, although
I've done my best to test it thoroughly.  Most of the problems will most
likely be compiler issues.

### glFont 2.0 API

The glFont 2.0 API consists of two files: glfont2.h and glfont2.cpp, and is
written in standard C++ .  These files must obviously
be included in your project or makefile so that they can be compiled and linked
into your program.

The glFont API uses std::pair and std::basic\_string, so make sure you have
<utility> and <string> included in your source file before you
include glfont2.h.  glFont also depends on OpenGL, of course, so also make
sure <GL/gl.h> is included.

#### glFont Initialization

glfont2.h defines the glfont namespace which includes a single class, GLFont,
which replaces the GLFONT structure in the old 1.0 C API.  glfont::GLFont
contains everything needed to create and display a glFont in a C++
program.  The GLFont class constructor takes no parameters, so the first
step is to create a GLFont object somewhere.  You will then call Create and
give it the file name of the .glf file to load as well as the texture name to
use.

```
#include <string>
#include <utility>
```

```
#ifdef _WIN32
#include <windows.h>
#endif
#include <GL/gl.h>
#include <GL/glu.h>
```

```
#include "glfont2.h"
using namespace glfont;
```

```
//glFont
GLFont myfont;
```

```
//Out OpenGL initialization function
bool TestBedInit (void)
{
	//Initialize OpenGL
	//Texture mapping and blending must be enabled for glFont to work
	glClearColor(0.0, 0.0, 0.0, 1.0);	
	glEnable(GL_TEXTURE_2D);
	glEnable(GL_BLEND);
	glBlendFunc(GL_SRC_ALPHA, GL_ONE_MINUS_SRC_ALPHA);
```

```
	//Create our glFont from verdana.glf, using texture 1
	if (!myfont.Create("verdana.glf", 1))
		return false;
```

```
	//Return successfully
	return true;
}
```

#### Drawing Text

Text output is accomplished via a call to one of the many versions of the
GLFont object's DrawString method.  DrawString is actually implemented
using templates, so it accepts both character arrays and STL strings.  In
fact, any of the the DrawString variations will accept char[], wchar\_t[],
std::string, and std::wstring.  This leaves it open to you to decided in
what format you would like to store your text, as well as maintaining
performance, since the compiler will create specialized versions of this method
depending on what you use--no conversions or casts necessary.  Yes, this
means that glFont can display wide character strings, as long as your glFont
includes the characters you wish to display in it's character interval.  If
DrawString encounters a character outside of the interval, it just skips
it.

Once you have initialized your GLFont object with an actual font created by
the glFont builder, you're almost ready to display some text.  Before you
call the DrawString variations, you should call the Begin method of your GLFont
object.  Currently, this simply binds the glFont texture.  You can do
this yourself if you wish.  If you don't call Begin (or take care yourself)
you might end up using a different texture.  Not a good idea.

```
void TestBedRender (void)
{
	float top_color[3] = {1.0F, 1.0F, 1.0F};
	float bottom_color[3] = {0.0F, 0.0F, 1.0F};
```

```
	//Clear back buffer
	glClear(GL_COLOR_BUFFER_BIT);
```

```
	//Draw some stuff
	glMatrixMode(GL_MODELVIEW);
	glLoadIdentity();
```

```
	//Draw a string
	glColor3f(0.0F, 0.0F, 1.0F);
	myfont.Begin();
	myfont.DrawString("Hello World!", 2.0F, 0.0F, 480.0F);
	myfont.DrawString(L"Hello World!", 2.0F, 0.0F,
		400.0F, top_color, bottom_color);
	myfont.DrawString(std::string("Hello World!"), 0.0F, 320.0F);
	myfont.DrawString(std::wstring(L"Hello World!"), 0.0F, 280.F,
		top_color, bottom_color);
	glTranslatef(0.0F, 200.F, 0.0F);
	glRotatef(15.0F, 0.0F, 0.0F, 1.0F);
	myfont.DrawString("Hello World!", 2.0F, 0.0F, 0.F,
		top_color, bottom_color);
}
```

You'll notice that I've thrown in there some char[], wchar\_t[], std::string,
and std::wstring strings in there.  They all work great.  Like the
original API, standard OpenGL calls can be used to set the color, size, and
position of text output with glFont.  However, the new API makes it easier
for you to use color and position the text without having to use OpenGL
calls.  DrawString by default displays the text at it's native size,
meaning that if a character is 10 pixels wide in the glFont texture, it will be
10 units wide when displayed (of course the units are relative to the the size
of your OpenGL viewport and projection setup).  There is a variation of
DrawString which takes a scaling factor.  A scaling factor of 2.0F will
double the size of the text.  There are also variations for setting a top
and bottom color for the text, creating a nice gradient look.  There are
lots of things you can do with it, and of course you can always extend it
yourself.  The new API has no equivalent to the old glFontEnd
function.  When you're done you're done.

Drawing text with glFont is as simple as that.

#### Reference

The following is a reference of all the methods of the GLFont class.

**GLFont Constructor**

```
	//Constructor
	GLFont ();
```

The constructor of the GLFont class simply initializes the object to a safe
state.  If you attempt to draw text using a GLFont object that hasn't been
created with the Create method, nothing will happen.

**GLFont Destructor**

```
	//Destructor
	~GLFont ();
```

The GLFont destructor destroys the glFont and frees any allocated memory.

**Create Method**

```
	//Creates the glFont
	bool Create (const char *file_name, int tex);
	bool Create (const std::string &file_name, int tex);
```

The Create method creates the glFont by loading the font texture and
character information from the specified file, and associating it with the
specified OpenGL texture.  It returns true if the creation was successful,
and false if not.

**Destroy Method**

```
	//Destroys the glFont
	void Destroy (void);
```

The Destroy method destroys the font by freeing any used memory and restoring
the GLFont object back to an uninitialized state.  You may call Destroy on
a GLFont object and then call Create again on that same object.

**Texture Size Retrieval Methods**

```
	//Texture size retrieval methods
	void GetTexSize (std::pair<int, int> *size);
	int GetTexWidth (void);
	int GetTexHeight (void);
```

These methods return the size of the glFont texture, in pixels.  The
first method takes the address of a std::pair<int, int> object and places
the width in the size->first member, and the height in the size->second
member.

The next two functions simply return the width and height, respectively.

**Character Interval
Retrieval Methods**

```
	//Character interval retrieval methods
	void GetCharInterval (std::pair<int, int> *interval);
	int GetStartChar (void);
	int GetEndChar (void);
```

These methods return the character interval (the starting and ending
character codes of the font).  They behave like the texture functions
above.  The starting character is placed in interval->first, the ending
character in interval->second.

**Character Size Retrieval
Methods**

```
	//Character size retrieval methods
	void GetCharSize (int c, std::pair<int, int> *size);
	int GetCharWidth (int c);
	int GetCharHeight (int c);
```

These methods return the size in pixels of individual characters in the
font.  They take an integer identifying the character and otherwise behave
exactly like the methods above.

**String Size Calculation**

```
	template<class T> void GetStringSize (const T *text,
		std::pair<int, int> *size);
	template<class T> void GetStringSize (const std::basic_string<T> &text,
		std::pair<int, int> *size);
```

These methods calculate the size in pixels of the given string.  The
std::pair size is used as above.

**Begin Method**

```
	//Begins text output with this font
	void Begin (void);
```

Begins drawing text with this font by binding to the glFont texture.

**DrawString Methods**

```
	template<class T> void DrawString (const T *text, float x,
		float y);
	template<class T> void DrawString (const std::basic_string<T> &text,
		float x, float y);
```

Draws the specified string at position (x, y) using the natural character
size.

```
	template<class T> void DrawString (const T *text, float scalar,
		float x, float y);
	template<class T> void DrawString (const std::basic_string<T> &text,
		float scalar, float x, float y);
```

Draws the specified string at position (x, y) using the scaling factor
scalar.

```
	template<class T> void DrawString (const T *text, float x,
		float y, const float *top_color, const float *bottom_color);
	template<class T> void DrawString (const std::basic_string<T> &text,
		float x, float y, const float *top_color, const float *bottom_color);
```

Draws the specified string at position (x, y) using the specified colors for
the top and bottom of the font, creating a nice blending gradient look. 
The color should point to an array of three floats, like in the glColor3fv call.

```
	template<class T> void DrawString (const T *text, float scalar,
		float x, float y, const float *top_color, const float *bottom_color);
	template<class T> void DrawString (const std::basic_string<T> &text,
		float scalar, float x, float y, const float *top_color,
		const float *bottom_color);
```

Combines the scaling and color functionality.  Should be pretty straight
forward.

#### Conclusion

As you can see, there's lots you can do with it.  I hope it makes it a
lot easier for people to use text in their OpenGL programs.  You should be
able to write additional functions to do things like center text, or even layer
another texture on top.  There's lots more I could have included, but I
want to release this as soon as possible.

### Appendix

Here is the entire file of the code I used to develop the API.  It
contains all of the non-OS specific OpenGL initialization and rendering
code.  Should be useful.

```
//STL headers
#include <string>
#include <utility>
```

```
//OpenGL support
#ifdef _WIN32
#include <windows.h>
#endif
#include <gl/gl.h>
#include <gl/glu.h>
```

```
//glFont headers
#include "glfont2.h"
using namespace glfont;
```

```
//Header for this file
#include "testbed.h"
```

```
//glFont
GLFont myfont;
```

```
//*********************************************************
//Functions
//*********************************************************
bool TestBedInit (void)
{
	//Initialize OpenGL
	glClearColor(0.0, 0.0, 0.0, 1.0);	
	glEnable(GL_TEXTURE_2D);
	glEnable(GL_BLEND);
	glBlendFunc(GL_SRC_ALPHA, GL_ONE_MINUS_SRC_ALPHA);
```

```
	//Try and load our font
	if (!myfont.Create("verdana.glf", 1))
		return false;
```

```
	//Return successfully
	return true;
}
//*********************************************************
void TestBedShutdown (void)
{
	//Destroy our font
	myfont.Destroy();
}
//*********************************************************
void TestBedViewport (int width, int height)
{
	//Initialize the viewport
	glViewport(0, 0, width, height);
```

```
	//Initialize OpenGL projection matrix
	glMatrixMode(GL_PROJECTION);
	glLoadIdentity();
	gluOrtho2D(0.0, 640.0, 0.0, 480.0);
}
//*********************************************************
void TestBedRender (void)
{
	float top_color[3] = {1.0F, 1.0F, 1.0F};
	float bottom_color[3] = {0.0F, 0.0F, 1.0F};
```

```
	//Clear back buffer
	glClear(GL_COLOR_BUFFER_BIT);
```

```
	//Initialize modelview matrix
	glMatrixMode(GL_MODELVIEW);
	glLoadIdentity();
```

```
	//Draw some strings
	glColor3f(0.0F, 0.0F, 1.0F);
	myfont.Begin();
	myfont.DrawString("Hello World!", 2.0F, 0.0F, 480.0F);
	myfont.DrawString(L"Hello World!", 2.0F, 0.0F,
		400.0F, top_color, bottom_color);
	myfont.DrawString(std::string("Hello World!"), 0.0F, 320.0F);
	myfont.DrawString(std::wstring(L"Hello World!"), 0.0F, 280.F,
		top_color, bottom_color);
	glTranslatef(0.0F, 200.F, 0.0F);
	glRotatef(15.0F, 0.0F, 0.0F, 1.0F);
	myfont.DrawString("Hello World!", 2.0F, 0.0F, 0.F,
		top_color, bottom_color);
}
//*********************************************************
```

```
//End of file
```

#### The End

mailto:bhf5@email.byu.edu
