# Supplementary material for: ProteinShader: illustrative rendering of macromolecules
Source: BMC Struct Biol. 2009 Mar 30;9:19. doi: 10.1186/1472-6807-9-19 (PMC2672931; doi:10.1186/1472-6807-9-19)
Supplement: Additional file 1 — ProteinShader program without source code. This compressed file contains the complete ProteinShader program including associated libraries, but no source code. A README.txt file gives an overview of the ProteinShader distribution, and the index.html file in the help subdirectory has directions on getting started with the program as well as a set of tutorials. [file 1472-6807-9-19-S1.zip › ProteinShader-beta-0_9_4-binary/help/gui-controls/cartoon.html]

ProteinShader: Cartoon Controls


# ProteinShader: Cartoon Controls


♦ Home


♦ Download


♦ Getting Started

Choose a Link:

Installation

Running

Troubleshooting

PDB Files


♦ Tutorials

Choose a Link:

List of Proteins

Style Options

Saving Images


♦ General Controls

Choose a Link:

Menu Bar

Keyboard Shortcuts

Mouse Motion

Motion Panel

Antialiasing


♦ Cartoon Controls

Choose a Link:

Control Panels

Selection Panel

Radio Buttons

Decorations Panel

Adding Textures

Color Panel

Visibility Panel

Side Chains Panel


♦ Atom Controls

Choose a Link:

Control Panels

Selection Panel

Radio Buttons

Color Panel

Visibility Panel

Scale Panel

Tiling Panel


♦ Developers

Choose a Link:

Compiling

Running

Code Docs

Developer Links


## Topics

Cartoon Control Panels  

Selection Panel  

Radio Buttons  

Decorations Panel  

Adding Textures  

Cartoon Color Panel  

Cartoon Visibility Panel  

Cartoon Side Chains Panel


## Cartoon Control Panels

After a cartoon-style display is selected from the
Style menu
above the canvas, the appearance of the tubes and ribbons can be modified
by using the retractable control panel on the right side of the canvas. The
control panel can be opened and closed by using the
Tools menu
in the menu bar above the canvas. The control panel is composed of two parts:
a left-side selection panel that remains fixed and a right-side modifier panel
that can be changed by using the menu at the top right of the control panel
(see the Control Panel figure further below). After explaining the left-side
selection panel, the rest of this page will cover the right-side modifier
subpanels that affect a cartoon-style display: Decorations, Cartoon Color,
Cartoon Visibility, and Cartoon Side Chains.


## Selection Panel


The selection subpanel can be seen on the left side of the control panel shown
right below. The same selection panel is used for both Atom-type (spheres and
cylinders) and Cartoon-style (tubes and ribbons) displays. There are four
kinds of selections:

- #### Model

  If a protein structure is determined by magnetic resonance imaging
  (NMR), there will typically be several possible models in the Protein
  Data Bank file. The ProteinShader program will read in all of the
  models, and the model to display can be selected from the Model menu
  at the top left of the control panel. Models are normally designated
  by integers, starting at 1. If a protein structure is determined by
  x-ray crystallography, there will usually be only a single model.

- #### Chain

  A model will always have at least one polypeptide chain, and might have
  several. Chains are usually designated by uppercase letters, starting
  at A.

- #### Amino Acids, Heterogens, or Waters

  The menu right below the Chain menu can be used to select Amino Acids,
  Heterogens, or Water. The list below the menu will display the
  residues (the term residue is being used rather broadly to include
  heterogens and water because they are specified in a similar manner
  to amino acids in a PDB file). To select a continuous range of
  residues, hold down the shift key while using the mouse to left click
  list items. To select a discontinous range of residues, use the
  control key (command key on Macintosh OS X) instead of the shift key.

- #### Atom

  Pressing the button labeled Atoms will open up a small dialog box with a
  list of the atoms for the first item currently selected in the list of
  residues (an amino acid, heterogen, or water). Atoms can be selected
  from the list, but that feature is only useful when a modifier panel
  for an atom-style display is being used. For a cartoon-style display
  (tubes or ribbons), colors and other modifications are applied by
  selecting amino acids or groups of amino acids (a region, chain, or
  model), as discussed further below.


## Radio Buttons

The top half of each cartoon subpanel contains radio buttons that will affect
where modifications are applied. Each radio button has an associated menu, and
the set of radio buttons with associated menus is the same for each of the
cartoon subpanels (Decorations, Cartoon Color, Cartoon Visibility, and Cartoon
Side Chains).

- #### Selected

  Choosing Model from the associated menu will apply any modifications to
  all of the currently displayed model, while choosing Chain will apply
  the modification to only the chain currently selected in the Chain
  menu on the left side of the control panel. If Residues is selected,
  then any modifications will be applied to the length of a tube or
  ribbon that corresponds to whatever amino acid or amino acids are
  currently selected in the list on the left side of the control panel.

- #### Helices

  If a PDB file has any helix records (α-helix or related), then
  they will be listed in this menu so that they can be selected. The
  unique name of each helix is taken from a HELIX record in the PDB file.

- #### β-Strands

  If a PDB file has any β-Strand records, then they will be listed in
  this menu so that they can be selected. Each β-strand is given a
  unique name by using its chain ID and the residue IDs of its first and
  last amino acids. This naming scheme is used to prevent duplicate
  β-strands with different names: authors of PDB records sometimes
  list two different SHEET records for the same stretch of amino acids
  because the β-strand is considered as belonging to two different
  β-sheets.

- #### Loops

  The term loop is being used very broadly here, so any stretch of a
  polypeptide chain that is not a helix or β-strand is considered a
  general loop region. The loops are numbered sequentially starting at 1
  (so loop 1 is the loop nearest the amino-terminus of the first chain,
  and the highest loop number will be for the loop nearest the
  carboxyl-terminus of the last chain).

- #### Global

  If one of the twenty amino acids is selected in this menu, then any
  modifications will be applied globally to all occurrences of that amino
  acid.


## Decorations Panel


The ProteinShader program uses small programs, known as vertex and fragment
shaders, to apply two-dimensional texture maps and custom lighting calculations
to the curved surfaces of three-dimensional tubes and ribbons. By using the
radio buttons and selection subpanel discussed in the previous two sections,
these special effects can be applied to an entire model or region, or to a
segment of a tube or ribbon that correspond to an individual amino acid.

- #### Halftoning

  When a structure is first loaded by selecting Open from the File menu
  above the canvas, it will automatically be shown as a pen-and-ink style
  tubes cartoon. This pen-and-ink style is accomplished partly by using
  a texture mapping technique called real-time halftoning. The image is
  converted from color to gray scale, and a two-dimensional texture image
  is repeatedly mapped onto the curved surface of the three-dimensional
  tubes or ribbons. Dark lines are also added to the edges of the tubes
  or ribbons. If the Extra Lines check box is selected, any tubes or
  ribbons shown with halftoning will have lines drawn to indicate the
  length of a tube or ribbon that corresponds to an amino acid.

  The best menu choice for halftoning is usually the Noise texture shown
  in the Halftoning Texture menu. A Bend Texture can also be selected
  to add what looks like hand-drawn lines to the middle of each segment
  of a tube or ribbon (where a segment is defined as the length of a tube
  or ribbon that corresponds to an amino acid). The halftoning texture
  is mixed in with lighting calculations, while the bend texture
  intensity is scaled by how bent the segment is (*i.e.*, the bend
  texture will be dark if the segment has a strong bend to it, but faint
  if the segment is almost straight).

- #### Patterns

  Using the Patterns button or menu will cause the current selection to
  be shown in color with the image from the menu mapped onto each segment
  of the tube or ribbon. For tubes, the image is wrapped around twice,
  and for ribbons the same image is mapped onto both broad surfaces of
  each ribbon segment.

- #### Text Labels

  The tubes or ribbons will be shown in color and a text label with the
  single-letter amino acid code and residue number will be mapped onto
  the curved surface of each segment. The left-to-right order of the
  labels corresponds to the amino-to-carboxyl direction of the
  polypeptide chain. Mapping labels onto curved surfaces is of limited
  value in a static image of a protein, but is much more useful in an
  image that is being moved around.

- #### Wireframe

  The tubes or ribbons will be shown in color and with a black wireframe
  that connects the vertices that were used to define the curved surfaces
  of the tube or ribbon.

- #### Plain

  The tubes or ribbons are shown in color, and with no texture mapping
  effects. Phong lighting calculations, which include specular
  highlighting (a shiny plastic or metallic look), are used. When color
  is used, the caps shown at the end of tubes or ribbons are color
  coded using blue for the amino-terminus and red for the
  carboxyl-terminus.


## Adding Textures

The textures found in the Halftone Texture, Bend Texture, and Patterns menus of
the Decorations panel
are specified by easy to modify configuration files
that are read when the ProteinShader program starts up. Each menu has its own
configuration file, which is found in a subdirectory of the
`textures` directory:

Halftone Textures: `halftoning/halftoning_textures.conf`  
Bend Textures: `bend/bend_textures.conf`  
Pattens Textures: `patterns/patterns_textures.conf`

The purpose of each configuration file is to associate texture files (PNG
or JPEG images) with menu names. The assocations are specified one per line
by giving the desired menu name followed by the equal sign and then a file
name. The configuration files are simple ASCII text files, so to modify a
configuration file, use a text editor, not a word processor, which will use
many hidden symbols.

Here is an example of the `patterns_textures.conf` file:  
  
`Vertical Bars = vbars.png  
Horizontal Bars = hbars.png  
Hash = hash.png  
Noise = noise.png  
Swirl = swirl.png  
Square = square.png  
Test = test.png`

The image format type must be indicated by a lowercase three letter extension
at the end of the texture file name. Only ".png" (PNG) and
".jpg" (JPEG) texture files have been tested, but the texture loader
being used by the program should be able to recognize ".bmp" (BMP),
".gif" (GIF), and ".tga" (TGA) formats. If you cannot see
the dot followed by three letters on your image filenames, your operating
system may be set to hide extensions. In that case, the extension can probably
be seen by opening a properties or information box on a file.

The texture files in current use happen to be 256 by 256 pixels. Other sizes
can be accomodated, but a roughly square image works best because of the
dimensions of the tube or ribbon segments that the image will be mapped onto.
OpenGL used to have a restriction that each dimension of an image file had to be
equal to two rasied to some integer power, but that restriction has been
relaxed as of OpenGL 2.0.

The order in which menu names are listed in the configuration file determines
the order that the names will have in the menu. The program expects to find a
PNG or JPEG image file in the same directory as the configuration file it is
named in. If an image file cannot be found, the program will continue to load
as many textures as it can find, and afterwards will open a dialog box with
information on the missing file or files.


## Cartoon Color Panel


The selection subpanel and radio buttons discussed further above can be used to
apply a color change to an entire model, chain, or region, or to a segment of a
tube or ribbon that corresponds to one or more individual amino acids. The
Cartoon Color panel has three buttons for changing color on the current
selection:

- #### Chooser

  Opens a color chooser box for selecting any color.

- #### Region Type

  Colors the current selection by region type:  
    
  Helix: red  
  β-Strand: green  
  General loop: gray

- #### Amino Acid

  Colors the current selection based on amino acid type. The colors
  listed below are documented in more detail (with RGB values) in the
  AAColorEnum enumeration in the
  ProteinShader API.  
    
  `Black: unknown amino acid  
  Blue: LYS and ARG  
  Bright red: ASP and GLU  
  Cyan: ASN and GLN  
  Dark gray: ALA  
  Green: LEU, VAL, and ILE  
  Light gray: GLY  
  Mid blue: PHE and TYR  
  Orange: SER and THR  
  Pale blue: HIS  
  Peach: PRO  
  Pink: TRP  
  Yellow: CYS and MET`


## Cartoon Visibility Panel


The Cartoon Visibility subpanel provides fine-grained control of visibility in
a cartoon-type display (ribbons and/or tubes), whereas the
Visibility menu
above the canvas provides only a coarse level of control over whether amino
acids, heterogens, or waters are allowed to be displayed.
The selection subpanel and radio buttons discussed further above can be used to
apply a visibility status change to an entire model, chain, or region, or to a
segment of a tube or ribbon that corresponds to one or more amino acids. The
Cartoon Visibility panel has three buttons and a slider for changing the
visibility status on the current selection:

- #### Opaque

  Sets the current selection to visible and non-translucent.

- #### Invisible

  Sets the current selection to invisible.

- #### Translucent

  Sets the current selection to be semi-transparent. The percent
  translucency can be set from 0 to 100 by using the slider at the
  bottom of the panel, or by clicking the Translucent button after
  entering a number in the text box right below the button. A
  translucency of 99 % is nearly invisible, while 1 % is nearly opaque.


## Cartoon Side Chains Panel


Amino acid side chains are added to ribbons and tubes by using the Space
Filling, Balls and Sticks, or Sticks buttons on the Cartoon Side Chains subpanel,
and are removed by using the None button. Depending on what radio buttons and
menus have already been selected, this modification can be applied to individual
amino acids, groups of amino acids, or the entire model.

If Space Filling or Balls and Sticks is selected, α-carbons are only shown
if the "Show α-carbon" check box is selected before clicking on
the button. In a Balls and Sticks style display, the α-carbons will be
visible when ribbons are selected, but will be obscured in a tube-style display
because of the diameter of the tube. If Balls and Sticks or Sticks is selected,
α-carbon to β-carbon bonds are always included, but they will be
mostly obscured in a tube-style display.

Setting the side chains to be visible or invisible during a cartoon-style
display (ribbons or tubes) is controlled only by the Cartoon Side Chains panel.
However, if a side chain is made visible in cartoon mode, it translucency and
color can be set by using the Atom Visibility and Atom Color subpanels,
respectively.


---

|  |  |  |  |
| --- | --- | --- | --- |
| Hosted by |  |  |  |

Webmaster: Joe Weber,
joe.weber AT alumni.duke.edu
  
Web site last updated: December 30th, 2008
  
© 2007–2008 Joseph R. Weber
