# Supplementary material for: ProteinShader: illustrative rendering of macromolecules
Source: BMC Struct Biol. 2009 Mar 30;9:19. doi: 10.1186/1472-6807-9-19 (PMC2672931; doi:10.1186/1472-6807-9-19)
Supplement: Additional file 1 — ProteinShader program without source code. This compressed file contains the complete ProteinShader program including associated libraries, but no source code. A README.txt file gives an overview of the ProteinShader distribution, and the index.html file in the help subdirectory has directions on getting started with the program as well as a set of tutorials. [file 1472-6807-9-19-S1.zip › ProteinShader-beta-0_9_4-binary/help/gui-controls/atom.html]

ProteinShader: Atom Controls


# ProteinShader: Atom Controls


♦ Home


♦ Download


♦ Getting Started

Choose a Link:

Installation

Running

Troubleshooting

PDB Files


♦ Tutorials

Choose a Link:

List of Proteins

Style Options

Saving Images


♦ General Controls

Choose a Link:

Menu Bar

Keyboard Shortcuts

Mouse Motion

Motion Panel

Antialiasing


♦ Cartoon Controls

Choose a Link:

Control Panels

Selection Panel

Radio Buttons

Decorations Panel

Adding Textures

Color Panel

Visibility Panel

Side Chains Panel


♦ Atom Controls

Choose a Link:

Control Panels

Selection Panel

Radio Buttons

Color Panel

Visibility Panel

Scale Panel

Tiling Panel


♦ Developers

Choose a Link:

Compiling

Running

Code Docs

Developer Links


## Topics

Atom Control Panels  

Selection Panel  

Radio Buttons  

Atom Color Panel  

Atom Visibility Panel  

Atom Scale Panel  

Tiling Panel


## Atom Control Panels


In an atom-style display, atoms and bonds are represented by spheres and
cylinders, respectively. After an atom-style display (Space Filling, Balls
and Sticks, or Sticks) is selected from the Style menu above the canvas,
the appearance of the spheres and cylinders can be modified by using the
retractable control panel on the right side of the canvas. The control panel
can be opened and closed by using the Tools menu in the menu bar above the
canvas. The control panel is composed of two parts: a left-side selection
panel that remains fixed and a right-side modifier panel that can be changed
by using the menu at the top right of the control panel (see the Control
Panel figure further below). After explaining the left-side selection panel,
the rest of this page will cover the right-side modifier subpanels that affect
an atom-style display: Atom Color, Atom Visibility, Atom Scale, and Tiling.


## Selection Panel


The selection subpanel can be seen on the left side of the control panel shown
right below. The same selection panel is used for both Atom-style (spheres and
cylinders) and Cartoon-style (tubes and ribbons) displays. There are four
kinds of selections:

- #### Model

  If a protein structure is determined by magnetic resonance imaging
  (NMR), there will typically be several possible models in the Protein
  Data Bank file. The ProteinShader program will read in all of the
  models, and the model to display can be selected from the Model menu
  at the top left of the control panel. Models are normally designated
  by integers, starting at 1. If a protein structure is determined by
  x-ray crystallography, there will usually be only a single model.

- #### Chain

  A model will always have at least one polypeptide chain, and might have
  several. Chains are usually designated by uppercase letters, starting
  at A.

- #### Amino Acids, Heterogens, or Waters

  The menu right below the Chain menu can be used to select Amino Acids,
  Heterogens, or Water. The list below the menu will display the residues
  (the term residue is being used rather broadly to include heterogens and
  water because they are specified in a similar manner to amino acids in a
  PDB file). To select a continuous range of residues, hold down the
  shift key while using the mouse to left click list items. To select a
  discontinous range of residues, use the control key (command key on
  Macintosh OS X) instead of the shift key.

- #### Atom

  Pressing the button labeled Atoms will open up a small dialog box with
  a list of the atoms for the first item currently selected in the list
  of residues (an amino acid, heterogen, or water). One or more atoms
  can be selected from the list.


## Radio Buttons

The top half of the Atom Color, Atom Visibility, and Atom Scale subpanels
contains radio buttons that will affect where modifications are
applied. Each radio button has an associated menu, and the set of radio
buttons with associated menus is the same for these three atom subpanels.

- #### Selected

  If Model is chosen from the menu, modifications will be applied to all
  of the currently displayed model. If Model-AA, Model-HET, or Model-HOH
  is chosen, modifications will be applied to only the amino acids,
  heterogens, or waters, respectively, of the model.
    
    
  If Residues or Atoms is chosen, then modifications will be applied to
  the residues or atoms currently selected in the left half of the
  control panel. The term "residue" is being used somewhat
  broadly here in that it applies to heterogens or waters in addition
  to amino acids.
    
    
  If Chain is chosen from the menu, modifications will be applied to the
  chain currently selected in the left half of the control panel. If
  Chain-AA, Chain-HET, or Chain-HOH is chosen, modifications will be
  applied to only the amino acids, heterogens, or waters, respectively,
  of the chain.

- #### Helices

  If a PDB file has any helix records (α-helix or related), then
  they will be listed in this menu so that they can be selected. The
  unique name of each helix is taken from a HELIX record in the PDB file.

- #### β-Strands

  If a PDB file has any β-Strand records, they will be listed in
  this menu so that they can be selected. Each β-strand is given a
  unique name by using its chain ID and the residue IDs of its first and
  last amino acids. This naming scheme is used to prevent duplicate
  β-strands with different names: authors of PDB records sometimes
  list two different SHEET records for the same stretch of amino acids
  because the β-strand is considered as belonging to two different
  β-sheets.

- #### Loops

  The term loop is being used very broadly here, so any stretch of a
  polypeptide chain that is not a helix or β-strand is considered a
  general loop region. The loops are numbered sequentially starting at 1
  (so loop 1 is the loop nearest the amino-terminus of the first chain,
  and the highest loop number will be for the loop nearest the
  carboxyl-terminus of the last chain).

- #### Global

  If one of the twenty amino acids is chosen in this menu, then any
  modifications will be applied globally to all occurrences of that
  amino acid. If an atom type is chosen (C, H, N, O, or S), then any
  modifications will be applied globally to all occurrences of that
  atom type.

- #### Apply To

  This menu can be used to further restrict any choices made in the radio
  buttons and menus above it. Choosing Entire AA (for entire amino acid
  causes no restrictions, but choosing Backbone would restrict any changes
  to the amino acid backbone atoms N, CA, and C, and choosing Side Chain
  would cause changes to be applied to only amino acid side chains. The
  additional choices in the menu allow changes to be restricted to
  particular atom types: N, CA, C, or O.


## Atom Color Panel


The selection subpanel and radio buttons discussed further above can be used to
apply a color change to a model, chain, region, amino acid, or atom. The Atom
Color panel has three buttons for changing color on the current selection:

- #### Chooser

  Opens a color chooser box for selecting any color.

- #### Atom Type

  Colors the current selection by atom type using the CPK (Corey, Pauling,
  and Koltun) color scheme. The color scheme is documented in more detail
  in the CPKColorEnum and AtomEnum enumerations in the
  ProteinShader API.  
    
  `C: light gray  
  H: white  
  N: light blue  
  O: red  
  S: yellow`

- #### Amino Acid

  Colors the current selection based on amino acid type. The colors
  listed below are documented in more detail (with RGB values) in the
  AAColorEnum enumeration in the
  ProteinShader API.  
    
  `Black: unknown amino acid  
  Blue: LYS and ARG  
  Bright red: ASP and GLU  
  Cyan: ASN and GLN  
  Dark gray: ALA  
  Green: LEU, VAL, and ILE  
  Light gray: GLY  
  Mid blue: PHE and TYR  
  Orange: SER and THR  
  Pale blue: HIS  
  Peach: PRO  
  Pink: TRP  
  Yellow: CYS and MET`


## Atom Visibility Panel


The Atom Visibility subpanel provides fine-grained control of visibility in
an atom-style display (Space Filling, Balls and Sticks, or Sticks), whereas the
Visibility menu
above the canvas provides only a coarse level of control over whether amino
acids, heterogens, or waters are allowed to be displayed.
The selection subpanel and radio buttons discussed further above can be used to
apply a visibility status change to a model, chain, region, amino acid, or atom.
The Atom Visibility panel has three buttons and a slider for changing the
visibility status on the current selection:

- #### Opaque

  Sets the current selection to visible and non-translucent.

- #### Invisible

  Sets the current selection to invisible.

- #### Translucent

  Sets the current selection to be semi-transparent. The percent
  translucency can be set from 0 to 100 by using the slider at the
  bottom of the panel, or by clicking the Translucent button after
  entering a number in the text box right below the button. A
  translucency of 99 % is nearly invisible, while 1 % is nearly opaque.


## Atom Scale Panel


The selection subpanel and radio buttons discussed further above can be used to
apply an atom scale change to a model, chain, region, amino acid, or atom.
The scale changes apply to the radius of the spheres used in a Space Filling
or Balls and Sticks style display.

The default radius for spheres in a Space Filling-style display is the Van Der
Waals radius, while the default radius for spheres in a Balls and Sticks display
is the covalent radius multiplied by a BALL\_SCALE factor of 0.2. The Van Der
Waals radius and covalent radius for each atom type are given in the
AtomEnum enumeration of the
ProteinShader API, where the BALL\_SCALE
factor is also specified.


## Tiling Panel


Most graphics cards ultimately draw only points, lines, and flat polygons, so
three-dimensional curved surfaces are approximated by drawing a large number
of flat polygons that are tiled together to form a continuous surface, and
lighting effects are used to help smooth out their appearance. The larger the
number of polygons, the closer the image comes to a true curved surface, but
the slower the scene will be rendered.

In an atom-style display, the ProteinShader program uses camera distance to
calculate the level of detail that each sphere and cylinder is drawn at.
When the camera is zoomed in to look at a small region of a protein, a high
level of detail is used to provide better image quality. When the camera is
zoomed out to look at the whole protein, a lower level of detail is used so
that the image can be rotated more smoothly. This automatic calculation of
the level of detail can be shut off by deselecting the Auto Tiling checkbox
near the top of the Tiling panel. Once automatic tiling is shut off, the
controls below the checkbox can be used to apply a fixed level of detail
to spheres and cylinders.

As a way of expressing the level of detail that a sphere is drawn at, the
concept of a tiling number is used. A tiling number of n means that if the
sphere was drawn as a wireframe, the latitude lines would divide it into n
stacks from pole to pole and the longitude lines would divide it into n slices
around the equator. Therefore, the total number of flat panels that comprise
the surface of the sphere would be n-squared. The concept of tiling number
also applies to cylinders, except that the tiling number only refers to the
number of slices about the equator because a cylinder does not curve along its
length.

By using the radio buttons and menus of the Tiling panel, a fixed tiling number
can be set for the spheres used in a Space Filling or a Balls and Sticks display.
Similarly, a tiling number can be set for cylinders in a Balls and Sticks or
Sticks display. Tiling number can also be set for the small spheres that
are used as end caps for cylinders.


---

|  |  |  |  |
| --- | --- | --- | --- |
| Hosted by |  |  |  |

Webmaster: Joe Weber,
joe.weber AT alumni.duke.edu
  
Web site last updated: December 30th, 2008
  
© 2007–2008 Joseph R. Weber
