# Supplementary material for: ProteinShader: illustrative rendering of macromolecules
Source: BMC Struct Biol. 2009 Mar 30;9:19. doi: 10.1186/1472-6807-9-19 (PMC2672931; doi:10.1186/1472-6807-9-19)
Supplement: Additional file 1 — ProteinShader program without source code. This compressed file contains the complete ProteinShader program including associated libraries, but no source code. A README.txt file gives an overview of the ProteinShader distribution, and the index.html file in the help subdirectory has directions on getting started with the program as well as a set of tutorials. [file 1472-6807-9-19-S1.zip › ProteinShader-beta-0_9_4-binary/help/gui-controls/general.html]

ProteinShader: General Controls


# ProteinShader: General Controls


|  |
| --- |
|  |


♦ Home


♦ Download


♦ Getting Started

Choose a Link:

Installation

Running

Troubleshooting

PDB Files


♦ Tutorials

Choose a Link:

List of Proteins

Style Options

Saving Images


♦ General Controls

Choose a Link:

Menu Bar

Keyboard Shortcuts

Mouse Motion

Motion Panel

Antialiasing


♦ Cartoon Controls

Choose a Link:

Control Panels

Selection Panel

Radio Buttons

Decorations Panel

Adding Textures

Color Panel

Visibility Panel

Side Chains Panel


♦ Atom Controls

Choose a Link:

Control Panels

Selection Panel

Radio Buttons

Color Panel

Visibility Panel

Scale Panel

Tiling Panel


♦ Developers

Choose a Link:

Compiling

Running

Code Docs

Developer Links


## Topics

Menu Bar Above Canvas  

Keyboard Shortcuts  

Mouse Motion Control  

Motion Panel  

Antialiasing  

Antialiasing Panel

## Menu Bar Above Canvas


|

 Menu | Purpose |
| --- | --- |
|

 File | In addition to Open and Quit, there is an Export Image submenu that allows the image on the canvas to be saved as a PNG or JPEG file. The dialog box for Open will go to the `data` directory, where PDB formatted files should be placed. The dialog box for Export Image will save the image to the `images` directory by default. The PNG (Portable Network Graphics, www.libpng.org/pub/png) format provides compression with no data loss, while the JPEG (Joint Photographic Experts Group, www.jpeg.org) format allows some data loss (with, hopefully, little or no noticable loss of image quality) so that the image can be compressed to a (usually) smaller file size than would be acheived using PNG. The Export Image submenu also includes an "Invisible Canvas PNG..." option that saves the background as transparent. The ability to save a GIF (Graphics Interchange Format, en.wikipedia.org/wiki/GIF) image has also been added. |
|

 Style | The Cartoon submenu allows a protein to be displayed as Tubes, Ribbons, Ribbons and Tubes, or Frenet Frames. α-Helices and β-strands are shown with a larger tube radius or greater ribbon width than general loop regions. Ribbons and Tubes is the default style that a new structure open up with: α-helices are shown as tubes while β-strands and loops are ribbons. Frenet Frames is a debugging mode that shows the underlying spline and local coordinate frames that are used for drawing tubes and ribbons.    The Atom submenu offers Space Filling (spheres), Balls and Sticks (spheres and cylinders), or Sticks (cylinders). |
|

 Visibility | Determines which residue types are allowed to be visible. The menu choices are Amino Acids Allowed, Heterogens Allowed, and Waters Allowed. Only Amino Acids Allowed is automatically selected when a new protein structure is loaded. If a menu item is grayed out (non-selectable), then the structure does not have any of that residue type.    This menu is intended as a convenience for quickly changing the visibility of residue types. For more fine-grained control, use the Atom Visibility or Cartoon Visibility subpanels. |
|

 Orientation | Choosing Original resets the protein to the same orientation and camera distance as when it was first loaded. Choosing Front, Back, left, Right, Top, and Bottom affect the protein's orientation, but not the camera distance. |
|

 Background | Sets the background color on the canvas to Black, Light Gray, White, or opens a Chooser dialog box that can be used to select any color. |
|

 Tools | Opens and closes the control panel on the right side of the canvas. |
|

 Help | Opens the desktop's default web browser and loads the index.html file that is located in the `help` directory. |


## Keyboard Shortcuts

The keyboard controls listed below will only work if the drawing canvas has
the focus. Clicking anywhere on the canvas will give it the focus.


| Key | Action |
| --- | --- |
| control-O | Open - opens a file chooser box so that a PDB structure file can be selected. |
| control-Q | Quit - terminates the program. |
| i or I | Zooms camera in by 5 angstroms. |
| o or O | Zooms camera out by 5 angstroms. |
| D | Toggles debugging on or off. When debugging is on, the camera distance for each atom, tube segment, or ribbon segment is printed to standard out (only useful if the program was started from a terminal window). The center of a tube or ribbon segment is the xyz-coordinates of the α-carbon of the amino acid that the tube or ribbon segment represents. If automatic tiling is turned on, then the tiling number (level of detail) for spheres is also printed. |
| M | Toggles motion (constant rotation) between on and off (equivalent to using the Start or Stop button on the Motion Panel). |


## Mouse Motion Control

The mouse can be dragged across the canvas to rotate an image or zoom the
camera in or out. The mouse buttons for motion and camera control are explained
in the table right below. The image can also be rotated at constant speed by
using the Motion Panel described further down the page.

Many newer Macintosh OS X machines may have a two or three button mouse, in
which case the button controls will likely be the same as for Linux and
Windows XP. However, if only a single-button mouse is present, the shift
and command keys will need to be used in combination with the mouse button,
as indicated in the table.


*Mouse button controls for image and camera movement*

| Action | Linux or Windows XP | Mac OS X (single button) |
| Rotate image (x- or y-axis) | left-drag | mouse-drag |
| Rotate image (z-axis) | shift & right-drag | shift & command & mouse-drag |
| Pan camera | right-drag | command & mouse-drag |
| Zoom camera (z-axis) | center-drag or shift & left-drag | shift & mouse-drag |

  

For zooming the camera in or out with a center-drag (button two on a three
button mouse), the drag should be vertical. If the mouse has a scroll wheel,
that should also work for zooming the camera in or out. For rotating the image
on the z-axis of the view, the right-button drag with the shift key held down
should be horizontal.

When the window with the canvas has the focus (because it was clicked on),
the camera can also be zoomed in or out by using keyboard shortcuts. The
"i" key (upper or lowercase) will zoom the camera in by 5 angstroms,
while the "o" key (upper or lowercase) will zoom the camera out by
5 ansgstroms.


## Motion Panel


If the Motion subpanel is not already visible, select Motion from the menu at
the top right of the retractable Control Panel on the right side of the
ProteinShader GUI. When the Start button is clicked, the number of frames per
second (fps) for the animation will be reported in the box at the bottom of the
Motion subpanel, and also in red letters at the top left of the canvas.

The slider or text box for the Y-Axis can be used to specify the number of
degrees per second of rotation about the protein structures's y-axis.
Because the rotation speed is given in degrees per second, the speed will be
about the same on different machines. However, how smooth or jerky the
animation appears to be will depend on the number of frames per second that
can be rendered, and that rendering speed will depend on both how complex the
image is, and on how powerful the graphics card is. The X-Axis slider and text
box set the degrees per second for the x-axis, with the subtle distinction that
the rotation is about the x-axis of the view rather than the x-axis of the
protein structure itself.


## Antialiasing


The term aliasing refers to the jagged edges that are often produced when
rendering geometrically defined objects (see part A of Figure 1 below for
an example). Techniques for smoothing out these jagged edges are called
antialiasing. Aliasing occurs because of the discrete nature of pixels
[1],
and because a pixel is detemined to be inside or outside of an object based
on whether the center of the pixel falls within a mathematically calculated
imaginary boundary line.


|  |  |  |  |
| --- | --- | --- | --- |
| **A** | **B** | **C** | **D** |

**Figure 1.** Application of antialiasing to a pen-and-ink style tubes
display of the retinol-binding protein.
(A) Halftoning image with no antialiasing. The 140 by 210 pixels image was
taken from a monitor with a 72 pixels per inch resolution, where the aliasing
(jagged edge) effect is quite noticeable against a white background.
(B) The same as (A), except that the Antialiasing subpanel shown further below
has been used to partially smooth out the black edges of halftoning images by
adding gray pixels next to the edge lines.
(C) The same as (B), except that additional antialiasing has been performed by
jittering the entire scene 3 times and blending the images.
(D) The same as (C), except that scene is jittered 6 times.
The retinol-binding protein was loaded from file 1AQB.pdb
[2, 3].

  

### Antialiasing Panel

The Antialiasing subpanel of the ProteinShader GUI provides controls for
smoothing out jagged edges in order to produce publication quality images.


- #### Entire Scene

  The checkbox and menu in the top half of the Antialiasing subpanel can
  be used to apply antialiasing by jittering the entire scene from 2 to
  16 times and blending the images in an accumulation buffer (jittering
  means to move the image a fraction of a pixel each time it is rendered).
  The xy-coordinates used for jittering are taken from the OpenGL
  Programming Guide [4].

  Entire-scene antialising is primarily intended for smoothing out jagged
  edges right before
  saving images
  from the canvas as PNG or JPEG files. By default, the whole-scene
  antialiasing checkbox is not selected because rendering times may be
  dramatically slowed. If a scene is jittered n times, then rendering
  will take approximately n times longer, so jittering even 3 or 4 times
  might be unacceptably slow for an animation (constant rotation) unless
  a fairly high-end graphics card is being used. Jittering the entire
  scene may also slow down responsiveness to the GUI controls, so it is
  generally best to leave whole-scene antialiasing off while performing
  manipulations such as changing colors, visibility status, zooming in
  on an image, *etc*.

- #### Black edges of halftoning images

  When ribbons and tubes with halftoning (pen-and-ink style) are drawn
  against a white background, the darkened edges often appear to be
  quite jagged, as shown above in Figure 1A. If the checkbox in the
  bottom half of the Antialiasing panel is selected, the jagged edges
  are partially smoothed out by adding additional gray pixels outside of
  the black edges. This effect is accomplished by rendering a
  translucent black silhouette of each tube or ribbon segment before
  rendering with halftoning. The silhouette is drawn 4 times with a
  slightly offset (a half pixel up, down, left, or right).

  This silhouette-based antialiasing only affects halftoning images,
  not any of the color images. Rendering may be slowed down 2 to 3-fold,
  depending on the graphics card. By default, this type of antialiasing
  is turned on despite the performance cost. Without any antialiasing,
  rotating halftoning images against a white background can produce a
  very annoying kinetic effect at the darkened edges. However, it the
  Background menu above the canvas is used to change the background
  to black, the aliasing problem is less noticeable, so it may be ok to
  deselect the antialiasing option in order to speed up the frames per
  second for an animation.


## References

1.
Hill FS:
**Aliasing; Antialiasing Techniques.**
In: *Computer Graphics using OpenGL*
Second edn. Upper Saddle River, JN: Prentice Hall; 2000: 557-586.

2.
Zanotti G, Panzalorto M, Marcato A, Malpeli G, Folli C, Berni R:
**Structure of pig plasma retinol-binding protein at 1.65 A resolution.**
*Acta Crystallogr, Sect D,* 1998, **54**: 1049-1052.

3.
**Retinol-binding protein PDB entry 1AQB**
[http://www.rcsb.org/pdb/explore/explore.do?structureId=1AQB]

4.
Schreiner D, Woo M, Neider J, Davis T:
**The Accumulation Buffer.**
In: *OpenGL Programming Guide.*
Fifth edn. Upper Saddle River, JN: Addison-Wesley; 2005: 482-495.


---

|  |  |  |  |
| --- | --- | --- | --- |
| Hosted by |  |  |  |

Webmaster: Joe Weber,
joe.weber AT alumni.duke.edu
  
Web site last updated: December 30th, 2008
  
© 2007–2008 Joseph R. Weber
