# Supplementary material for: ProteinShader: illustrative rendering of macromolecules
Source: BMC Struct Biol. 2009 Mar 30;9:19. doi: 10.1186/1472-6807-9-19 (PMC2672931; doi:10.1186/1472-6807-9-19)
Supplement: Additional file 1 — ProteinShader program without source code. This compressed file contains the complete ProteinShader program including associated libraries, but no source code. A README.txt file gives an overview of the ProteinShader distribution, and the index.html file in the help subdirectory has directions on getting started with the program as well as a set of tutorials. [file 1472-6807-9-19-S1.zip › ProteinShader-beta-0_9_4-binary/help/api/org/proteinshader/math/class-use/Vec3d.html]

Uses of Class org.proteinshader.math.Vec3d (ProteinShader API)


|  |  |  |  |  |  |  |  |  |  |  |
| --- | --- | --- | --- | --- | --- | --- | --- | --- | --- | --- |
| |  |  |  |  |  |  |  |  | | --- | --- | --- | --- | --- | --- | --- | --- | | **Overview** | **Package** | **Class** | **Use** | **Tree** | **Deprecated** | **Index** | **Help** | | |  |
| PREV   NEXT | **FRAMES**    **NO FRAMES**     **All Classes** |


---


## **Uses of Class org.proteinshader.math.Vec3d**

| Packages that use Vec3d | |
| --- | --- |
| **org.proteinshader.gui.viewing** | Holds important helper classes that the Renderer uses to control the view: Camera, Rotation, Lighting, *etc*. |
| **org.proteinshader.math** | The key classes in this package are Hermite and Quaternion, which are needed for generating the ribbons and tubes that are used to represent the backbone of a protein in a cartoon-type display. |
| **org.proteinshader.structure** | Holds the classes that store information from a Protein Data Bank file: Structure, Model, Chain, AminoAcid, Heterogen, Water, Atom, Bond, Helix, BetaStrand, Loop, *etc*. |

| Uses of Vec3d in org.proteinshader.gui.viewing | |
| --- | --- |

| Fields in org.proteinshader.gui.viewing declared as Vec3d | |
| --- | --- |
| `static Vec3d` | `Rotation.X_AXIS`             Vector representing an x-axis. |
| `static Vec3d` | `Rotation.Y_AXIS`             Vector representing an y-axis. |
| `static Vec3d` | `Rotation.Z_AXIS`             Vector representing an z-axis. |

| Uses of Vec3d in org.proteinshader.math | |
| --- | --- |

| Methods in org.proteinshader.math that return Vec3d | |
| --- | --- |
| `Vec3d` | `Vec3d.add(Vec3d vec)`             Creates a new vector by adding the vector given as an argument to the calling vector (the existing vectors are NOT modified). |
| `Vec3d` | `Hermite.calculateReverseTangent(double t)`             Gets the tangent from the calculateTangent() method and reverses its direction. |
| `Vec3d` | `Hermite.calculateTangent(double t)`             Uses Hermite interpolation to calculate the tangent of a point on the cubic equation between the start and end points given the constructor. |
| `Vec3d` | `Hermite.calculateTranslation(double t)`             If the xyz-point obtained by calculatePoint() is going to be used to translate an object from the origin to the point, then it is convenient for a vector to be returned instead of a point. |
| `Vec3d` | `Vec3d.clone()`             Returns a clone of the calling vector. |
| `Vec3d` | `Vec3d.cross(Vec3d vec)`             Creates a new vector by taking the cross product of the calling vector and the vector given as an argument. |
| `Vec3d` | `Quaternion.getBinormal()`             Converts the Quaternion into a 3 x 3 rotation matrix and the returns the second column vector (the Binormal). |
| `Vec3d` | `LocalFrame.getBinormal()`             Converts the rotation (a Quaternion) into a 3 x 3 rotation matrix and the returns the second column vector (the Binormal). |
| `Vec3d` | `Quaternion.getNormal()`             Converts the Quaternion into a 3 x 3 rotation matrix and the returns the first column vector (the Normal). |
| `Vec3d` | `LocalFrame.getNormal()`             Converts the rotation (a Quaternion) into a 3 x 3 rotation matrix and the returns the first column vector (the Normal). |
| `Vec3d` | `LocalFrame.getReverseTangent()`             Converts the rotation (a Quaternion) into a 3 x 3 rotation matrix and the returns the third column vector (the Tangent) after reversing its direction. |
| `Vec3d` | `Quaternion.getTangent()`             Converts the Quaternion into a 3 x 3 rotation matrix and the returns the third column vector (the Tangent). |
| `Vec3d` | `LocalFrame.getTangent()`             Converts the rotation (a Quaternion) into a 3 x 3 rotation matrix and the returns the third column vector (the Tangent). |
| `Vec3d` | `LocalFrame.getTranslation()`             Returns a copy of the translation vector held by this LocalFrame. |
| `Vec3d` | `Point3d.minus(Point3d point)`             Returns the direction vector obtained by subtracting the point given as an argument from the calling point. |
| `Vec3d` | `Vec3d.minus(Vec3d vec)`             Creates a new vector by subtracting the vector given as an argument from the calling vector (the existing vectors are NOT modified). |
| `Vec3d` | `Quaternion.multiply(Vec3d v)`             Multiplies a vector by this quaternion and returns the resulting vector. |
| `Vec3d` | `Vec3d.negate()`             Creates a new vector equal in magnitude but opposite in direction to the calling vector (the calling vector is NOT modified). |
| `Vec3d` | `Vec3d.normalize()`             Creates a new vector that has the same direction as the calling vector, but is of unit length (the calling vector is NOT modified). |
| `Vec3d` | `LocalFrame.rotate(Vec3d vector)`             Returns a new vector produced by rotating the vector given as an argument by the quaternion held in this local frame. |
| `Vec3d` | `Vec3d.scale(double magnitude)`             Creates a new vector by scaling the calling vector by the magnitude given as an argument (the calling vector is NOT modified). |

| Methods in org.proteinshader.math with parameters of type Vec3d | |
| --- | --- |
| `Point3d` | `Point3d.add(Vec3d vec)`             Returns the point created by adding the vector given as an argument to the point that is the calling object. |
| `Vec3d` | `Vec3d.add(Vec3d vec)`             Creates a new vector by adding the vector given as an argument to the calling vector (the existing vectors are NOT modified). |
| `void` | `Point3d.addToMe(Vec3d vec)`             Tranlates the calling Point3d by adding the vector xyz-coordinates to it. |
| `void` | `Vec3d.addToMe(Vec3d vec)`             Modifies the calling vector by adding the xyz-values of the vector given as an argument to the xyz-values of the calling vector. |
| `void` | `Quaternion.adjustMyTangent(Vec3d tangent)`             Modifies this quaternion by rotating it such that if it is converted into a rotation matrix [N B T], the T vector will match the tangent given as an argument. |
| `Quaternion` | `Quaternion.adjustTangent(Vec3d tangent)`             Returns a copy of this quaternion that has been rotated such that if it is converted into a rotation matrix [N B T], the T vector will match the tangent given as an argument. |
| `void` | `QuaternionDemo.convertToQuaternionAndPrint(Vec3d N, Vec3d B, Vec3d T)`             Converts rotation matrix [N B T] to a quaternion and prints it. |
| `Vec3d` | `Vec3d.cross(Vec3d vec)`             Creates a new vector by taking the cross product of the calling vector and the vector given as an argument. |
| `void` | `Vec3d.crossMe(Vec3d vec)`             Modifies the calling vector by calculating the cross product of the calling vector and the vector given as an argument and storing the result in the calling vector. |
| `double` | `Vec3d.dot(Vec3d vec)`             Returns the dot product of the calling vector and the vector given as an argument. |
| `double` | `Quaternion.generateAxisAndAngle(Vec3d axis)`             Converts the Quaternion to an axis of rotation and an angle. |
| `void` | `Quaternion.generateMatrix(Vec3d N, Vec3d B, Vec3d T)`             Generates a 3 x 3 rotation matrix (actually 3 column vectors) that is equivalent to this quaternion, assuming that the quaternion has already been normalized (such that x^2 + y^2 + z^2 + w^2 = 1). |
| `void` | `LocalFrame.generateMatrix(Vec3d N, Vec3d B, Vec3d T)`             Generates a 3 x 3 rotation matrix (actually 3 column vectors) that is equivalent to the rotation quaternion. |
| `Vec3d` | `Vec3d.minus(Vec3d vec)`             Creates a new vector by subtracting the vector given as an argument from the calling vector (the existing vectors are NOT modified). |
| `void` | `Vec3d.minusFromMe(Vec3d vec)`             The x, y, and z values of the vector given as an argument are subtracted from the x, y, and z values of the calling vector. |
| `Vec3d` | `Quaternion.multiply(Vec3d v)`             Multiplies a vector by this quaternion and returns the resulting vector. |
| `void` | `QuaternionDemo.printColumnVectors(Vec3d N, Vec3d B, Vec3d T)`             Prints the 3 column vectors to standard out. |
| `void` | `SlerpDemo.printColumnVectors(Vec3d N, Vec3d B, Vec3d T)`             Prints the 3 column vectors to standard out. |
| `static void` | `HermiteDemo.printStartAndEndPoints(Point3d p1, Point3d p2, Vec3d tan1, Vec3d tan2)`             Prints the start and end points along with their tangents. |
| `Vec3d` | `LocalFrame.rotate(Vec3d vector)`             Returns a new vector produced by rotating the vector given as an argument by the quaternion held in this local frame. |
| `void` | `LocalFrame.setTranslation(Vec3d translation)`             Holds on the the vector given as an argument so that it can be used as the translation for the local coordinate frame. |
| `void` | `Vec3d.setXYZ(Vec3d vec)`             Sets the xyz-values of the vector to the xyz-values of the vector given as an argument. |
| `void` | `Quaternion.setXYZW(Vec3d axis, double angle)`             Calculates and sets (x, y, z, w) of this quaternion based on the axis and angle (in radians) given as aguments. |
| `void` | `Quaternion.setXYZW(Vec3d N, Vec3d B, Vec3d T)`             Calculates and sets (x, y, z, w) of this quaternion based on the rotation contained in the 3 x 3 matrix [N B T]. |

| Constructors in org.proteinshader.math with parameters of type Vec3d | |
| --- | --- |
| `Hermite(Point3d p1, Point3d p2, Vec3d tan1, Vec3d tan2)`             Constructs a Hermite object by using the input points and vectors to calculate and store the coefficients needed for the cubic equations for x(t), y(t), and z(t). |
| `Hermite(Vec3d p1, Vec3d p2, Vec3d tan1, Vec3d tan2)`             Constructs a Hermite object by using the input vectors to calculate and store the coefficients needed for the cubic equations for x(t), y(t), and z(t). |
| `LocalFrame(Quaternion rotation, Vec3d translation)`             Constructs a LocalFrame that holds on to the Quaternion and Vec3d given as arguments. |
| `LocalFrame(Vec3d N, Vec3d B, Vec3d T, double x, double y, double z)`             Constructs a LocalFrame equivalent to the rotation matrix [N B T] and the translation (x, y, z). |
| `Quaternion(Vec3d axis, double angle)`             Creates a quaternion that is equivalent to the rotation specified by the axis and angle (in radians) given as aguments. |
| `Quaternion(Vec3d N, Vec3d B, Vec3d T)`             Creates a quaternion that is equivalent to the rotation contained in the 3 x 3 matrix [N B T]. |

| Uses of Vec3d in org.proteinshader.structure | |
| --- | --- |

| Methods in org.proteinshader.structure that return Vec3d | |
| --- | --- |
| `Vec3d` | `Segment.getMiddleBinormal()`             Converts the middle rotation (a Quaternion) into a 3 x 3 rotation matrix and then returns the third column vector, the Binormal. |
| `Vec3d` | `Segment.getMiddleNormal()`             Converts the middle rotation (a Quaternion) into a 3 x 3 rotation matrix and then returns the first column vector, the Normal. |
| `Vec3d` | `Segment.getMiddleTangent()`             Converts the middle rotation (a Quaternion) into a 3 x 3 rotation matrix and then returns the third column vector, the Tangent. |
| `Vec3d` | `Segment.getMiddleXYZ()`             Copies the xyz-coordinates for the center of this Segment into a new Vec3d and returns it. |
| `Vec3d` | `AminoAcid.getTranslation()`             Returns a new Ved3d object based on the xyz-coordinates of the alpha-carbon. |
| `Vec3d` | `Drawable.getTranslation()`             Returns the xyz-center of the Drawable as a vector. |
| `Vec3d` | `Drawable.minus(Drawable other)`             Returns a vector created by subtracting the xyz-center of the Drawable given as an argument from the xyz-center of the calling Drawable object. |

---


|  |  |  |  |  |  |  |  |  |  |  |
| --- | --- | --- | --- | --- | --- | --- | --- | --- | --- | --- |
| |  |  |  |  |  |  |  |  | | --- | --- | --- | --- | --- | --- | --- | --- | | **Overview** | **Package** | **Class** | **Use** | **Tree** | **Deprecated** | **Index** | **Help** | | |  |
| PREV   NEXT | **FRAMES**    **NO FRAMES**     **All Classes** |


---

# *Copyright © 2007-2008*
