# Supplementary material for: ProteinShader: illustrative rendering of macromolecules
Source: BMC Struct Biol. 2009 Mar 30;9:19. doi: 10.1186/1472-6807-9-19 (PMC2672931; doi:10.1186/1472-6807-9-19)
Supplement: Additional file 1 — ProteinShader program without source code. This compressed file contains the complete ProteinShader program including associated libraries, but no source code. A README.txt file gives an overview of the ProteinShader distribution, and the index.html file in the help subdirectory has directions on getting started with the program as well as a set of tutorials. [file 1472-6807-9-19-S1.zip › ProteinShader-beta-0_9_4-binary/help/api/org/proteinshader/math/class-use/Quaternion.html]

Uses of Class org.proteinshader.math.Quaternion (ProteinShader API)


|  |  |  |  |  |  |  |  |  |  |  |
| --- | --- | --- | --- | --- | --- | --- | --- | --- | --- | --- |
| |  |  |  |  |  |  |  |  | | --- | --- | --- | --- | --- | --- | --- | --- | | **Overview** | **Package** | **Class** | **Use** | **Tree** | **Deprecated** | **Index** | **Help** | | |  |
| PREV   NEXT | **FRAMES**    **NO FRAMES**     **All Classes** |


---


## **Uses of Class org.proteinshader.math.Quaternion**

| Packages that use Quaternion | |
| --- | --- |
| **org.proteinshader.math** | The key classes in this package are Hermite and Quaternion, which are needed for generating the ribbons and tubes that are used to represent the backbone of a protein in a cartoon-type display. |
| **org.proteinshader.structure** | Holds the classes that store information from a Protein Data Bank file: Structure, Model, Chain, AminoAcid, Heterogen, Water, Atom, Bond, Helix, BetaStrand, Loop, *etc*. |
| **org.proteinshader.structure.visitor** | Holds Visitor classes that know how to traverse the hierarchy of objects held by class Structure. |

| Uses of Quaternion in org.proteinshader.math | |
| --- | --- |

| Methods in org.proteinshader.math that return Quaternion | |
| --- | --- |
| `Quaternion` | `Quaternion.adjustTangent(Vec3d tangent)`             Returns a copy of this quaternion that has been rotated such that if it is converted into a rotation matrix [N B T], the T vector will match the tangent given as an argument. |
| `Quaternion` | `Quaternion.clone()`             Creates a clone of this quaternion and returns it. |
| `Quaternion` | `Quaternion.conjugate()`             Creates a clone of the calling Quaternion and then converts it to the conjugate, q'. |
| `Quaternion` | `Quaternion.invert()`             Creates a clone of the calling Quaternion and inverts it. |
| `Quaternion` | `Quaternion.multiply(Quaternion quat)`             Creates a clone of the calling Quaternion and multiplies it by the Quaternion given as an argument. |
| `Quaternion` | `Quaternion.normalize()`             Creates and returns a new quaternion that is equivalent to the calling quaternion, but is guaranteed to be of unit length. |
| `Quaternion` | `Quaternion.slerp(Quaternion end, double t)`             Uses SLERP (Spherical Linear intERPolation) to calculate a Quaternion (a rotation) at any point t between the calling Quaternion and the argument Quaternion. |

| Methods in org.proteinshader.math with parameters of type Quaternion | |
| --- | --- |
| `double` | `Quaternion.angleBetweenNormals(Quaternion quat)`             Returns the angle between the normals of the calling and argument quaternions, where the normal is the first column vector of the rotation matrix [N B T] that is the equivalent of a quaternion. |
| `void` | `SlerpDemo.interpolateAndPrint(Quaternion quat1, Quaternion quat2, double t)`             Interpolates between the two quaternions given as arguments and then prints the result quaterion along with the [N B T] equivalent to the quaternion. |
| `Quaternion` | `Quaternion.multiply(Quaternion quat)`             Creates a clone of the calling Quaternion and multiplies it by the Quaternion given as an argument. |
| `void` | `Quaternion.multiplyMe(Quaternion quat)`             Multiplies the calling Quaternion by the argument Quaternion and stores the result in the calling Quaternion. |
| `void` | `QuaternionDemo.printInterpolation(Quaternion start, Quaternion end, double[] t)`             For each value in the array t, the slerp() method of class Quaternion will be used to calculate and print an interpolated Quaternion between the start and end Quaternions. |
| `void` | `LocalFrame.setRotation(Quaternion rotation)`             Holds on to the Quaternion given as an argument so that it can be used as the rotation of the local coordinate frame. |
| `void` | `Quaternion.setXYZW(Quaternion quat)`             Sets the xyzw-values of the quaternion to the xyzw-values of the quaternion given as an argument. |
| `Quaternion` | `Quaternion.slerp(Quaternion end, double t)`             Uses SLERP (Spherical Linear intERPolation) to calculate a Quaternion (a rotation) at any point t between the calling Quaternion and the argument Quaternion. |

| Constructors in org.proteinshader.math with parameters of type Quaternion | |
| --- | --- |
| `LocalFrame(Quaternion rotation, Vec3d translation)`             Constructs a LocalFrame that holds on to the Quaternion and Vec3d given as arguments. |

| Uses of Quaternion in org.proteinshader.structure | |
| --- | --- |

| Methods in org.proteinshader.structure that return Quaternion | |
| --- | --- |
| `Quaternion` | `Segment.getEndRotation()`             Returns a clone of the Quaternion for the end rotation of this Segment. |
| `Quaternion` | `Segment.getMiddleRotation()`             Returns a clone of the Quaternion for the middle rotation of this Segment. |
| `Quaternion` | `AminoAcid.getRotation()`             Returns the rotation (the equivalent of a Frenet Frame) that will be needed for creating a Segment object based on this AminoAcid. |
| `Quaternion` | `Segment.getStartRotation()`             Returns a clone of the Quaternion for the start rotation of this Segment. |

| Methods in org.proteinshader.structure with parameters of type Quaternion | |
| --- | --- |
| `void` | `AminoAcid.setRotation(Quaternion rotation)`             Sets the rotation (the equivalent of a Frenet Frame) that will be needed for creating a Segment object based on this AminoAcid. |

| Constructors in org.proteinshader.structure with parameters of type Quaternion | |
| --- | --- |
| `Segment(AminoAcid aminoAcid, Hermite hermite1, Hermite hermite2, Quaternion startRotation, Quaternion middleRotation, Quaternion endRotation, boolean alwaysCapStart, boolean alwaysCapEnd)`             Creates a Segment. |

| Uses of Quaternion in org.proteinshader.structure.visitor | |
| --- | --- |

| Methods in org.proteinshader.structure.visitor that return Quaternion | |
| --- | --- |
| `Quaternion` | `FrenetFrameGeneratorVisitor.calculateRotation(Atom atom1, Atom atom2, Atom atom3)`             Calculates a rotation matrix [N B T] based on the xyz-centers of the three Atoms given as arguments, and then converts the rotation matrix into a Quaternion that is returned. |

---


|  |  |  |  |  |  |  |  |  |  |  |
| --- | --- | --- | --- | --- | --- | --- | --- | --- | --- | --- |
| |  |  |  |  |  |  |  |  | | --- | --- | --- | --- | --- | --- | --- | --- | | **Overview** | **Package** | **Class** | **Use** | **Tree** | **Deprecated** | **Index** | **Help** | | |  |
| PREV   NEXT | **FRAMES**    **NO FRAMES**     **All Classes** |


---

# *Copyright © 2007-2008*
