# Supplementary material for: ProteinShader: illustrative rendering of macromolecules
Source: BMC Struct Biol. 2009 Mar 30;9:19. doi: 10.1186/1472-6807-9-19 (PMC2672931; doi:10.1186/1472-6807-9-19)
Supplement: Additional file 1 — ProteinShader program without source code. This compressed file contains the complete ProteinShader program including associated libraries, but no source code. A README.txt file gives an overview of the ProteinShader distribution, and the index.html file in the help subdirectory has directions on getting started with the program as well as a set of tutorials. [file 1472-6807-9-19-S1.zip › ProteinShader-beta-0_9_4-binary/help/api/org/proteinshader/math/class-use/LocalFrame.html]

Uses of Class org.proteinshader.math.LocalFrame (ProteinShader API)


|  |  |  |  |  |  |  |  |  |  |  |
| --- | --- | --- | --- | --- | --- | --- | --- | --- | --- | --- |
| |  |  |  |  |  |  |  |  | | --- | --- | --- | --- | --- | --- | --- | --- | | **Overview** | **Package** | **Class** | **Use** | **Tree** | **Deprecated** | **Index** | **Help** | | |  |
| PREV   NEXT | **FRAMES**    **NO FRAMES**     **All Classes** |


---


## **Uses of Class org.proteinshader.math.LocalFrame**

| Packages that use LocalFrame | |
| --- | --- |
| **org.proteinshader.graphics** | Holds the drawing classes: Ribbon, Tube, FrenetFrames, Sphere, and Cylinder. |
| **org.proteinshader.math** | The key classes in this package are Hermite and Quaternion, which are needed for generating the ribbons and tubes that are used to represent the backbone of a protein in a cartoon-type display. |
| **org.proteinshader.structure** | Holds the classes that store information from a Protein Data Bank file: Structure, Model, Chain, AminoAcid, Heterogen, Water, Atom, Bond, Helix, BetaStrand, Loop, *etc*. |
| **org.proteinshader.structure.io** | Holds the classes needed for reading Protein Data Bank structure files. |

| Uses of LocalFrame in org.proteinshader.graphics | |
| --- | --- |

| Methods in org.proteinshader.graphics with parameters of type LocalFrame | |
| --- | --- |
| `int` | `ExtrudedShape.createDisplayList(GL gl, LocalFrame[] frames)`             Uses an array of LocalFrame objects to draw a segment of a tube and saves the geometry (a collection of vertices) as an OpenGL display list. |
| `void` | `Ribbon.createDisplayLists(GL gl, LocalFrame[] frames, SegmentListInfo info)`             Creates an OpenGL display list for the thin sides of the ribbon, and also calls on the createDisplayLists() method of superclass ExtrudedShape to save the OpenGL display lists for the main (broad surface) part of the ribbon and for the start and end caps. |
| `void` | `ExtrudedShape.createDisplayLists(GL gl, LocalFrame[] frames, SegmentListInfo info)`             Creates three OpenGL display lists: one created by calling draw(), one created by calling drawStartCap(), and one created by calling drawEndCap(). |
| `int` | `ExtrudedShape.createEndCapDisplayList(GL gl, LocalFrame[] frames)`             Uses the last LocalFrame in the array to position a segment end cap that is saved as an OpenGL display list. |
| `int` | `ExtrudedShape.createStartCapDisplayList(GL gl, LocalFrame[] frames)`             Uses the first LocalFrame in the array to position a segment start cap that is saved as an OpenGL display list. |
| `int` | `Ribbon.createThinSidesDisplayList(GL gl, LocalFrame[] frames)`             Caches an OpenGL display list for the thin sides of a segment of a ribbon. |
| `void` | `Ribbon.draw(GL gl, LocalFrame[] frames)`             Uses an array of LocalFrame objects to draw the broad surfaces of of a ribbon. |
| `abstract  void` | `ExtrudedShape.draw(GL gl, LocalFrame[] frames)`             Uses an array of LocalFrame objects to draw a segment of an extruded shape. |
| `void` | `Tube.draw(GL gl, LocalFrame[] frames)`             Uses an array of LocalFrame objects to draw a segment of a tube. |
| `void` | `FrenetFrames.draw(GL gl, LocalFrame[] frames)`             Uses an array of LocalFrame objects to draw xyz-axes. |
| `void` | `FrenetFrames.draw(GL gl, LocalFrame frame, double scale, double sphereRadius, int sphereTiling, float sphereRed, float sphereGreen, float sphereBlue, float sphereAlpha)`             Draws an xyz-axis based on the rotation and translation in the LocalFrame given as an argument. |
| `void` | `Ribbon.drawEndCap(GL gl, LocalFrame frame)`             Creates a cap for the end of a segment by drawing the waist polygon. |
| `abstract  void` | `ExtrudedShape.drawEndCap(GL gl, LocalFrame frame)`             Creates a cap for the end of a segment by drawing the waist polygon. |
| `void` | `Tube.drawEndCap(GL gl, LocalFrame frame)`             Creates a cap for the end of a segment by drawing the waist polygon. |
| `void` | `FrenetFrames.drawEndCap(GL gl, LocalFrame frame)`             Draws the local frame using red for the sphere at the base. |
| `void` | `Ribbon.drawStartCap(GL gl, LocalFrame frame)`             Creates a cap for the beginning of a segment by drawing the waist polygon. |
| `abstract  void` | `ExtrudedShape.drawStartCap(GL gl, LocalFrame frame)`             Creates a cap for the beginning of a segment by drawing the waist polygon. |
| `void` | `Tube.drawStartCap(GL gl, LocalFrame frame)`             Creates a cap for the beginning of a segment by drawing the waist polygon. |
| `void` | `FrenetFrames.drawStartCap(GL gl, LocalFrame frame)`             Draws the local frame using light blue for the sphere at the base. |
| `void` | `Ribbon.drawThinSideSurfaces(GL gl, LocalFrame[] frames)`             Draws the thin side surfaces for a segment of a ribbon. |

| Uses of LocalFrame in org.proteinshader.math | |
| --- | --- |

| Methods in org.proteinshader.math that return LocalFrame | |
| --- | --- |
| `LocalFrame` | `LocalFrame.clone()`             Returns a clone of the calling LocalFrame. |

| Uses of LocalFrame in org.proteinshader.structure | |
| --- | --- |

| Methods in org.proteinshader.structure that return LocalFrame | |
| --- | --- |
| `LocalFrame` | `Segment.getLocalFrame(double t)`             Creates a LocalFrame along the Segment spline by using Hermite interpolation and SLERP (Spherical Linear intERPolation). |
| `LocalFrame[]` | `Segment.getLocalFrames(int n)`             Uses SLERP (Spherical Linear intERPolation) and Hermite interpolation to generate the requested number of LocalFrames from the start frame to the end frame, inclusive. |

| Uses of LocalFrame in org.proteinshader.structure.io | |
| --- | --- |

| Methods in org.proteinshader.structure.io with parameters of type LocalFrame | |
| --- | --- |
| `double[][]` | `TubeVertices.draw(LocalFrame[] frames)`             Uses an array of LocalFrame objects to draw a segment of a tube. |

---


|  |  |  |  |  |  |  |  |  |  |  |
| --- | --- | --- | --- | --- | --- | --- | --- | --- | --- | --- |
| |  |  |  |  |  |  |  |  | | --- | --- | --- | --- | --- | --- | --- | --- | | **Overview** | **Package** | **Class** | **Use** | **Tree** | **Deprecated** | **Index** | **Help** | | |  |
| PREV   NEXT | **FRAMES**    **NO FRAMES**     **All Classes** |


---

# *Copyright © 2007-2008*
