# Supplementary material for: ProteinShader: illustrative rendering of macromolecules
Source: BMC Struct Biol. 2009 Mar 30;9:19. doi: 10.1186/1472-6807-9-19 (PMC2672931; doi:10.1186/1472-6807-9-19)
Supplement: Additional file 1 — ProteinShader program without source code. This compressed file contains the complete ProteinShader program including associated libraries, but no source code. A README.txt file gives an overview of the ProteinShader distribution, and the index.html file in the help subdirectory has directions on getting started with the program as well as a set of tutorials. [file 1472-6807-9-19-S1.zip › ProteinShader-beta-0_9_4-binary/help/api/org/proteinshader/math/class-use/HermiteDemo.html]

Uses of Class org.proteinshader.math.HermiteDemo (ProteinShader API)


|  |  |  |  |  |  |  |  |  |  |  |
| --- | --- | --- | --- | --- | --- | --- | --- | --- | --- | --- |
| |  |  |  |  |  |  |  |  | | --- | --- | --- | --- | --- | --- | --- | --- | | **Overview** | **Package** | **Class** | **Use** | **Tree** | **Deprecated** | **Index** | **Help** | | |  |
| PREV   NEXT | **FRAMES**    **NO FRAMES**     **All Classes** |


---


## **Uses of Class org.proteinshader.math.HermiteDemo**

No usage of org.proteinshader.math.HermiteDemo

---


|  |  |  |  |  |  |  |  |  |  |  |
| --- | --- | --- | --- | --- | --- | --- | --- | --- | --- | --- |
| |  |  |  |  |  |  |  |  | | --- | --- | --- | --- | --- | --- | --- | --- | | **Overview** | **Package** | **Class** | **Use** | **Tree** | **Deprecated** | **Index** | **Help** | | |  |
| PREV   NEXT | **FRAMES**    **NO FRAMES**     **All Classes** |


---

# *Copyright © 2007-2008*
