# Supplementary material for: ProteinShader: illustrative rendering of macromolecules
Source: BMC Struct Biol. 2009 Mar 30;9:19. doi: 10.1186/1472-6807-9-19 (PMC2672931; doi:10.1186/1472-6807-9-19)
Supplement: Additional file 1 — ProteinShader program without source code. This compressed file contains the complete ProteinShader program including associated libraries, but no source code. A README.txt file gives an overview of the ProteinShader distribution, and the index.html file in the help subdirectory has directions on getting started with the program as well as a set of tutorials. [file 1472-6807-9-19-S1.zip › ProteinShader-beta-0_9_4-binary/help/api/org/proteinshader/math/class-use/Hermite.html]

Uses of Class org.proteinshader.math.Hermite (ProteinShader API)


|  |  |  |  |  |  |  |  |  |  |  |
| --- | --- | --- | --- | --- | --- | --- | --- | --- | --- | --- |
| |  |  |  |  |  |  |  |  | | --- | --- | --- | --- | --- | --- | --- | --- | | **Overview** | **Package** | **Class** | **Use** | **Tree** | **Deprecated** | **Index** | **Help** | | |  |
| PREV   NEXT | **FRAMES**    **NO FRAMES**     **All Classes** |


---


## **Uses of Class org.proteinshader.math.Hermite**

| Packages that use Hermite | |
| --- | --- |
| **org.proteinshader.math** | The key classes in this package are Hermite and Quaternion, which are needed for generating the ribbons and tubes that are used to represent the backbone of a protein in a cartoon-type display. |
| **org.proteinshader.structure** | Holds the classes that store information from a Protein Data Bank file: Structure, Model, Chain, AminoAcid, Heterogen, Water, Atom, Bond, Helix, BetaStrand, Loop, *etc*. |

| Uses of Hermite in org.proteinshader.math | |
| --- | --- |

| Methods in org.proteinshader.math that return Hermite | |
| --- | --- |
| `Hermite` | `Hermite.clone()`             Creates a clone of the calling Hermite object and returns it. |

| Methods in org.proteinshader.math with parameters of type Hermite | |
| --- | --- |
| `static void` | `HermiteDemo.printCurve(Hermite hermite)`             Uses the Hermite object to obtain and print interpolated points from parameter t = 0.0 to t = 1.0. |

| Uses of Hermite in org.proteinshader.structure | |
| --- | --- |

| Methods in org.proteinshader.structure that return Hermite | |
| --- | --- |
| `Hermite` | `Segment.getHermite1()`             Creates a clone of the hermite1 object and returns it. |
| `Hermite` | `Segment.getHermite2()`             Creates a clone of the hermite2 object and returns it. |

| Constructors in org.proteinshader.structure with parameters of type Hermite | |
| --- | --- |
| `Segment(AminoAcid aminoAcid, Hermite hermite1, Hermite hermite2, Quaternion startRotation, Quaternion middleRotation, Quaternion endRotation, boolean alwaysCapStart, boolean alwaysCapEnd)`             Creates a Segment. |

---


|  |  |  |  |  |  |  |  |  |  |  |
| --- | --- | --- | --- | --- | --- | --- | --- | --- | --- | --- |
| |  |  |  |  |  |  |  |  | | --- | --- | --- | --- | --- | --- | --- | --- | | **Overview** | **Package** | **Class** | **Use** | **Tree** | **Deprecated** | **Index** | **Help** | | |  |
| PREV   NEXT | **FRAMES**    **NO FRAMES**     **All Classes** |


---

# *Copyright © 2007-2008*
