# Supplementary material for: ProteinShader: illustrative rendering of macromolecules
Source: BMC Struct Biol. 2009 Mar 30;9:19. doi: 10.1186/1472-6807-9-19 (PMC2672931; doi:10.1186/1472-6807-9-19)
Supplement: Additional file 1 — ProteinShader program without source code. This compressed file contains the complete ProteinShader program including associated libraries, but no source code. A README.txt file gives an overview of the ProteinShader distribution, and the index.html file in the help subdirectory has directions on getting started with the program as well as a set of tutorials. [file 1472-6807-9-19-S1.zip › ProteinShader-beta-0_9_4-binary/help/api/org/proteinshader/math/class-use/Point3d.html]

Uses of Class org.proteinshader.math.Point3d (ProteinShader API)


|  |  |  |  |  |  |  |  |  |  |  |
| --- | --- | --- | --- | --- | --- | --- | --- | --- | --- | --- |
| |  |  |  |  |  |  |  |  | | --- | --- | --- | --- | --- | --- | --- | --- | | **Overview** | **Package** | **Class** | **Use** | **Tree** | **Deprecated** | **Index** | **Help** | | |  |
| PREV   NEXT | **FRAMES**    **NO FRAMES**     **All Classes** |


---


## **Uses of Class org.proteinshader.math.Point3d**

| Packages that use Point3d | |
| --- | --- |
| **org.proteinshader.math** | The key classes in this package are Hermite and Quaternion, which are needed for generating the ribbons and tubes that are used to represent the backbone of a protein in a cartoon-type display. |
| **org.proteinshader.structure** | Holds the classes that store information from a Protein Data Bank file: Structure, Model, Chain, AminoAcid, Heterogen, Water, Atom, Bond, Helix, BetaStrand, Loop, *etc*. |

| Uses of Point3d in org.proteinshader.math | |
| --- | --- |

| Methods in org.proteinshader.math that return Point3d | |
| --- | --- |
| `Point3d` | `Point3d.add(Vec3d vec)`             Returns the point created by adding the vector given as an argument to the point that is the calling object. |
| `Point3d` | `Hermite.calculatePoint(double t)`             Uses Hermite interpolation to calculate a point on the curve between the start and end points given the constructor. |
| `Point3d` | `Point3d.clone()`             Returns a clone of this point. |
| `Point3d` | `Quaternion.multiply(Point3d p)`             Multiplies a point by this quaternion and returns the resulting point. |
| `Point3d` | `LocalFrame.multiply(Point3d vertex)`             Multiplies the vertex by the local frame. |
| `Point3d` | `LocalFrame.rotate(Point3d point)`             Returns a new point produced by rotating the point given as an argument by the quaternion held in this local frame. |

| Methods in org.proteinshader.math with parameters of type Point3d | |
| --- | --- |
| `Vec3d` | `Point3d.minus(Point3d point)`             Returns the direction vector obtained by subtracting the point given as an argument from the calling point. |
| `Point3d` | `Quaternion.multiply(Point3d p)`             Multiplies a point by this quaternion and returns the resulting point. |
| `Point3d` | `LocalFrame.multiply(Point3d vertex)`             Multiplies the vertex by the local frame. |
| `static void` | `HermiteDemo.printStartAndEndPoints(Point3d p1, Point3d p2, Vec3d tan1, Vec3d tan2)`             Prints the start and end points along with their tangents. |
| `Point3d` | `LocalFrame.rotate(Point3d point)`             Returns a new point produced by rotating the point given as an argument by the quaternion held in this local frame. |
| `void` | `LocalFrame.translate(Point3d vertex)`             The vertex given as an argument is modified by adding to it the xyz-coordinates of the translation vector held by this LocalFrame. |

| Constructors in org.proteinshader.math with parameters of type Point3d | |
| --- | --- |
| `Hermite(Point3d p1, Point3d p2, Vec3d tan1, Vec3d tan2)`             Constructs a Hermite object by using the input points and vectors to calculate and store the coefficients needed for the cubic equations for x(t), y(t), and z(t). |

| Uses of Point3d in org.proteinshader.structure | |
| --- | --- |

| Methods in org.proteinshader.structure that return Point3d | |
| --- | --- |
| `Point3d` | `AminoAcid.getCenter()`             Returns a new Point3d object based on the xyz-coordinates of the alpha-carbon. |
| `Point3d` | `Drawable.getPoint()`             Returns the xyz-center of the Drawable as a point. |

---


|  |  |  |  |  |  |  |  |  |  |  |
| --- | --- | --- | --- | --- | --- | --- | --- | --- | --- | --- |
| |  |  |  |  |  |  |  |  | | --- | --- | --- | --- | --- | --- | --- | --- | | **Overview** | **Package** | **Class** | **Use** | **Tree** | **Deprecated** | **Index** | **Help** | | |  |
| PREV   NEXT | **FRAMES**    **NO FRAMES**     **All Classes** |


---

# *Copyright © 2007-2008*
