# Supplementary material for: ProteinShader: illustrative rendering of macromolecules
Source: BMC Struct Biol. 2009 Mar 30;9:19. doi: 10.1186/1472-6807-9-19 (PMC2672931; doi:10.1186/1472-6807-9-19)
Supplement: Additional file 1 — ProteinShader program without source code. This compressed file contains the complete ProteinShader program including associated libraries, but no source code. A README.txt file gives an overview of the ProteinShader distribution, and the index.html file in the help subdirectory has directions on getting started with the program as well as a set of tutorials. [file 1472-6807-9-19-S1.zip › ProteinShader-beta-0_9_4-binary/help/api/org/proteinshader/math/VectorAndPointDemo.html]

VectorAndPointDemo (ProteinShader API)


|  |  |  |  |  |  |  |  |  |  |  |
| --- | --- | --- | --- | --- | --- | --- | --- | --- | --- | --- |
| |  |  |  |  |  |  |  |  | | --- | --- | --- | --- | --- | --- | --- | --- | | **Overview** | **Package** | **Class** | **Use** | **Tree** | **Deprecated** | **Index** | **Help** | | |  |
| **PREV CLASS**   NEXT CLASS | **FRAMES**    **NO FRAMES**     **All Classes** |
| SUMMARY: NESTED | FIELD | CONSTR | METHOD | DETAIL: FIELD | CONSTR | METHOD |


---


## org.proteinshader.math Class VectorAndPointDemo

```
java.lang.Object
  org.proteinshader.math.VectorAndPointDemo
```

---

``` public class VectorAndPointDemo extends Object ```

Performs some simple tests on the Vec3d and Point3d classes.

---

| **Constructor Summary** | |
| --- | --- |
| `VectorAndPointDemo()` |


| **Method Summary** | |
| --- | --- |
| `static void` | `main(String[] args)`             Performs some simple tests (constructors, setXYZ, cross product, dot product, and normalization) with Vec3d objects, and a few tests with Point3d objects. |

| **Methods inherited from class java.lang.Object** |
| --- |
| `clone, equals, finalize, getClass, hashCode, notify, notifyAll, toString, wait, wait, wait` |

| **Constructor Detail** |
| --- |

### VectorAndPointDemo

```
public VectorAndPointDemo()
```


| **Method Detail** |
| --- |

### main

```
public static void main(String[] args)
```

:   Performs some simple tests (constructors, setXYZ, cross product,
    dot product, and normalization) with Vec3d objects, and a few
    tests with Point3d objects.


---


|  |  |  |  |  |  |  |  |  |  |  |
| --- | --- | --- | --- | --- | --- | --- | --- | --- | --- | --- |
| |  |  |  |  |  |  |  |  | | --- | --- | --- | --- | --- | --- | --- | --- | | **Overview** | **Package** | **Class** | **Use** | **Tree** | **Deprecated** | **Index** | **Help** | | |  |
| **PREV CLASS**   NEXT CLASS | **FRAMES**    **NO FRAMES**     **All Classes** |
| SUMMARY: NESTED | FIELD | CONSTR | METHOD | DETAIL: FIELD | CONSTR | METHOD |


---

# *Copyright © 2007-2008*
