# Supplementary material for: ProteinShader: illustrative rendering of macromolecules
Source: BMC Struct Biol. 2009 Mar 30;9:19. doi: 10.1186/1472-6807-9-19 (PMC2672931; doi:10.1186/1472-6807-9-19)
Supplement: Additional file 1 — ProteinShader program without source code. This compressed file contains the complete ProteinShader program including associated libraries, but no source code. A README.txt file gives an overview of the ProteinShader distribution, and the index.html file in the help subdirectory has directions on getting started with the program as well as a set of tutorials. [file 1472-6807-9-19-S1.zip › ProteinShader-beta-0_9_4-binary/help/api/org/proteinshader/math/LocalFrameDemo.html]

LocalFrameDemo (ProteinShader API)


|  |  |  |  |  |  |  |  |  |  |  |
| --- | --- | --- | --- | --- | --- | --- | --- | --- | --- | --- |
| |  |  |  |  |  |  |  |  | | --- | --- | --- | --- | --- | --- | --- | --- | | **Overview** | **Package** | **Class** | **Use** | **Tree** | **Deprecated** | **Index** | **Help** | | |  |
| **PREV CLASS**   **NEXT CLASS** | **FRAMES**    **NO FRAMES**     **All Classes** |
| SUMMARY: NESTED | FIELD | CONSTR | METHOD | DETAIL: FIELD | CONSTR | METHOD |


---


## org.proteinshader.math Class LocalFrameDemo

```
java.lang.Object
  org.proteinshader.math.LocalFrameDemo
```

---

``` public class LocalFrameDemo extends Object ```

Performs some simple tests on the LocalFrame class.

---

| **Field Summary** | |
| --- | --- |
| `static int` | `BANNER_WIDTH`             The banner width is the number of asterisks to print on the line line above or below a banner title. |


| **Constructor Summary** | |
| --- | --- |
| `LocalFrameDemo()`             Creates a LocalFrameDemo object with a few Quaternion objects that can be used for testing purposes. |


| **Method Summary** | |
| --- | --- |
| `String` | `createRepeats(char ch, int n)`             Returns a String with the character given as an argument repeated n number of times. |
| `static void` | `main(String[] args)`             Tests the constructors, setter methods, and multiply(Point3d) methods of the LocalFrame class. |
| `void` | `printBanner(String title, String message)`             Prints a banner with a row of asterisks above and below the title, and then a message right below the banner. |
| `void` | `test2ArgConstructor()`             Uses a rotation quaternion and a translation vector to create a LocalFrame and then prints the LocalFrame by using its toString() method. |
| `void` | `test6ArgConstructor()`             Uses a matrix of 3 column vectors, [N B T], and 3 doubles (x, y, z) to create a LocalFrame and then prints the LocalFrame. |
| `void` | `test7ArgConstructor()`             Uses xyzw-values for a quaternion and xyz-values for a translation vector to create a LocalFrame and then prints the LocalFrame. |
| `void` | `testMultiplyPointMethod()`             Tests the multiply(Point3d) method of LocalFrame. |
| `void` | `testSetRotationMethods()`             Creates a LocalFrame with the zero-arg constructor and then tests the setRotation() methods. |
| `void` | `testSetTranslationMethods()`             Creates a LocalFrame with the zero-arg constructor and then tests the setTranslation() methods. |
| `void` | `testZeroArgConstructor()`             Uses the zero-argument constructor to create and then print a LocalFrame. |

| **Methods inherited from class java.lang.Object** |
| --- |
| `clone, equals, finalize, getClass, hashCode, notify, notifyAll, toString, wait, wait, wait` |

| **Field Detail** |
| --- |

### BANNER\_WIDTH

```
public static final int BANNER_WIDTH
```

:   The banner width is the number of asterisks
    to print on the line line above or below a
    banner title. The value is set to 70.

    **See Also:**: Constant Field Values


| **Constructor Detail** |
| --- |

### LocalFrameDemo

```
public LocalFrameDemo()
```

:   Creates a LocalFrameDemo object with a few Quaternion objects that
    can be used for testing purposes.


| **Method Detail** |
| --- |

### test2ArgConstructor

```
public void test2ArgConstructor()
```

:   Uses a rotation quaternion and a translation vector to create a
    LocalFrame and then prints the LocalFrame by using its toString()
    method.

---


### test6ArgConstructor

```
public void test6ArgConstructor()
```

:   Uses a matrix of 3 column vectors, [N B T], and 3 doubles
    (x, y, z) to create a LocalFrame and then prints the LocalFrame.

---


### test7ArgConstructor

```
public void test7ArgConstructor()
```

:   Uses xyzw-values for a quaternion and xyz-values for a translation
    vector to create a LocalFrame and then prints the LocalFrame.

---


### testZeroArgConstructor

```
public void testZeroArgConstructor()
```

:   Uses the zero-argument constructor to create and then print a
    LocalFrame.

---


### testSetRotationMethods

```
public void testSetRotationMethods()
```

:   Creates a LocalFrame with the zero-arg constructor and then tests
    the setRotation() methods.

---


### testSetTranslationMethods

```
public void testSetTranslationMethods()
```

:   Creates a LocalFrame with the zero-arg constructor and then tests
    the setTranslation() methods.

---


### testMultiplyPointMethod

```
public void testMultiplyPointMethod()
```

:   Tests the multiply(Point3d) method of LocalFrame. Simple
    rotations (90 x-roll and -90 z-roll) are used along with points
    on the x-, y-, or z-axis in order to make the results easy to
    judge for correctness (by making a simple pencil-and-paper sketch
    of the xyz-coordinate system).

---


### printBanner

```
public void printBanner(String title,
                        String message)
```

:   Prints a banner with a row of asterisks above and below the title,
    and then a message right below the banner.

    :   **Parameters:**: `title` - the title to place in the banner.: `message` - a message to print right below the banner.

---


### createRepeats

```
public String createRepeats(char ch,
                            int n)
```

:   Returns a String with the character given as an argument repeated
    n number of times.

    :   **Parameters:**: `ch` - the char to repeat.: `n` - the number of times to repeat ch. **Returns:**: A String with n repeats of ch.

---


### main

```
public static void main(String[] args)
```

:   Tests the constructors, setter methods, and multiply(Point3d)
    methods of the LocalFrame class.


---


|  |  |  |  |  |  |  |  |  |  |  |
| --- | --- | --- | --- | --- | --- | --- | --- | --- | --- | --- |
| |  |  |  |  |  |  |  |  | | --- | --- | --- | --- | --- | --- | --- | --- | | **Overview** | **Package** | **Class** | **Use** | **Tree** | **Deprecated** | **Index** | **Help** | | |  |
| **PREV CLASS**   **NEXT CLASS** | **FRAMES**    **NO FRAMES**     **All Classes** |
| SUMMARY: NESTED | FIELD | CONSTR | METHOD | DETAIL: FIELD | CONSTR | METHOD |


---

# *Copyright © 2007-2008*
