# Supplementary material for: ProteinShader: illustrative rendering of macromolecules
Source: BMC Struct Biol. 2009 Mar 30;9:19. doi: 10.1186/1472-6807-9-19 (PMC2672931; doi:10.1186/1472-6807-9-19)
Supplement: Additional file 1 — ProteinShader program without source code. This compressed file contains the complete ProteinShader program including associated libraries, but no source code. A README.txt file gives an overview of the ProteinShader distribution, and the index.html file in the help subdirectory has directions on getting started with the program as well as a set of tutorials. [file 1472-6807-9-19-S1.zip › ProteinShader-beta-0_9_4-binary/help/api/org/proteinshader/math/package-summary.html]

org.proteinshader.math (ProteinShader API)


|  |  |  |  |  |  |  |  |  |  |  |
| --- | --- | --- | --- | --- | --- | --- | --- | --- | --- | --- |
| |  |  |  |  |  |  |  |  | | --- | --- | --- | --- | --- | --- | --- | --- | | **Overview** | **Package** | Class | **Use** | **Tree** | **Deprecated** | **Index** | **Help** | | |  |
| **PREV PACKAGE**   **NEXT PACKAGE** | **FRAMES**    **NO FRAMES**     **All Classes** |


---

## Package org.proteinshader.math

The key classes in this package are Hermite and Quaternion, which are needed
for generating the ribbons and tubes that are used to represent the backbone
of a protein in a cartoon-type display.

**See:**
  
          **Description**

| **Class Summary** | |
| --- | --- |
| **Hermite** | Calculates a cubic equation between two control points so that points on the curve in between can be interpolated. |
| **HermiteDemo** | Performs some simple tests on the Hermite class. |
| **LocalFrame** | Stores a local coordinate frame as a rotation (a Quaternion) and a translation (a Point3d). |
| **LocalFrameDemo** | Performs some simple tests on the LocalFrame class. |
| **Point3d** | This class is used to create a point with 3 elements of type double. |
| **Quaternion** | This class is used to create a quaternion, a four-dimensional complex number that is typically used to represent a rotation in three-dimensional space. |
| **QuaternionDemo** | Performs several tests on the Quaternion class, including a test of the SLERP (Spherical Linear intERPolation) algorithm. |
| **SlerpDemo** | Tests the SLERP algorithm. |
| **Vec3d** | This class is used to create a vector with 3 elements of type double. |
| **VectorAndPointDemo** | Performs some simple tests on the Vec3d and Point3d classes. |

## Package org.proteinshader.math Description

The key classes in this package are Hermite and Quaternion, which are needed
for generating the ribbons and tubes that are used to represent the backbone
of a protein in a cartoon-type display.
