# Supplementary material for: ProteinShader: illustrative rendering of macromolecules
Source: BMC Struct Biol. 2009 Mar 30;9:19. doi: 10.1186/1472-6807-9-19 (PMC2672931; doi:10.1186/1472-6807-9-19)
Supplement: Additional file 1 — ProteinShader program without source code. This compressed file contains the complete ProteinShader program including associated libraries, but no source code. A README.txt file gives an overview of the ProteinShader distribution, and the index.html file in the help subdirectory has directions on getting started with the program as well as a set of tutorials. [file 1472-6807-9-19-S1.zip › ProteinShader-beta-0_9_4-binary/help/api/org/proteinshader/math/HermiteDemo.html]

HermiteDemo (ProteinShader API)


|  |  |  |  |  |  |  |  |  |  |  |
| --- | --- | --- | --- | --- | --- | --- | --- | --- | --- | --- |
| |  |  |  |  |  |  |  |  | | --- | --- | --- | --- | --- | --- | --- | --- | | **Overview** | **Package** | **Class** | **Use** | **Tree** | **Deprecated** | **Index** | **Help** | | |  |
| **PREV CLASS**   **NEXT CLASS** | **FRAMES**    **NO FRAMES**     **All Classes** |
| SUMMARY: NESTED | FIELD | CONSTR | METHOD | DETAIL: FIELD | CONSTR | METHOD |


---


## org.proteinshader.math Class HermiteDemo

```
java.lang.Object
  org.proteinshader.math.HermiteDemo
```

---

``` public class HermiteDemo extends Object ```

Performs some simple tests on the Hermite class.

---

| **Field Summary** | |
| --- | --- |
| `static int` | `SEGMENTS`             The number of segments to divide the curve into is 10, so the total number of points printed will be 11, including the points for t = 0.0 and t = 1.0. |


| **Constructor Summary** | |
| --- | --- |
| `HermiteDemo()` |


| **Method Summary** | |
| --- | --- |
| `static void` | `main(String[] args)`             Creates a start and end point (each with a tangent vector) to plug into a Hermite object. |
| `static void` | `printCurve(Hermite hermite)`             Uses the Hermite object to obtain and print interpolated points from parameter t = 0.0 to t = 1.0. |
| `static void` | `printStartAndEndPoints(Point3d p1, Point3d p2, Vec3d tan1, Vec3d tan2)`             Prints the start and end points along with their tangents. |

| **Methods inherited from class java.lang.Object** |
| --- |
| `clone, equals, finalize, getClass, hashCode, notify, notifyAll, toString, wait, wait, wait` |

| **Field Detail** |
| --- |

### SEGMENTS

```
public static final int SEGMENTS
```

:   The number of segments to divide the curve into is 10,
    so the total number of points printed will be 11,
    including the points for t = 0.0 and t = 1.0.

    **See Also:**: Constant Field Values


| **Constructor Detail** |
| --- |

### HermiteDemo

```
public HermiteDemo()
```


| **Method Detail** |
| --- |

### main

```
public static void main(String[] args)
```

:   Creates a start and end point (each with a tangent vector)
    to plug into a Hermite object. A helper method is then used to
    increment parameter t from 0.0 to 1.0 while printing the
    points (and tangents) in between the start and end points.
    When graphed, these point should form a curve.

---


### printStartAndEndPoints

```
public static void printStartAndEndPoints(Point3d p1,
                                          Point3d p2,
                                          Vec3d tan1,
                                          Vec3d tan2)
```

:   Prints the start and end points along with their tangents.

    :   **Parameters:**: `p1` - the start point: `p2` - the end point: `tan1` - the tangent of the start point.: `tan2` - the tangent of the end point.

---


### printCurve

```
public static void printCurve(Hermite hermite)
```

:   Uses the Hermite object to obtain and print interpolated points
    from parameter t = 0.0 to t = 1.0.


---


|  |  |  |  |  |  |  |  |  |  |  |
| --- | --- | --- | --- | --- | --- | --- | --- | --- | --- | --- |
| |  |  |  |  |  |  |  |  | | --- | --- | --- | --- | --- | --- | --- | --- | | **Overview** | **Package** | **Class** | **Use** | **Tree** | **Deprecated** | **Index** | **Help** | | |  |
| **PREV CLASS**   **NEXT CLASS** | **FRAMES**    **NO FRAMES**     **All Classes** |
| SUMMARY: NESTED | FIELD | CONSTR | METHOD | DETAIL: FIELD | CONSTR | METHOD |


---

# *Copyright © 2007-2008*
