# Supplementary material for: ProteinShader: illustrative rendering of macromolecules
Source: BMC Struct Biol. 2009 Mar 30;9:19. doi: 10.1186/1472-6807-9-19 (PMC2672931; doi:10.1186/1472-6807-9-19)
Supplement: Additional file 1 — ProteinShader program without source code. This compressed file contains the complete ProteinShader program including associated libraries, but no source code. A README.txt file gives an overview of the ProteinShader distribution, and the index.html file in the help subdirectory has directions on getting started with the program as well as a set of tutorials. [file 1472-6807-9-19-S1.zip › ProteinShader-beta-0_9_4-binary/help/api/org/proteinshader/math/package-use.html]

Uses of Package org.proteinshader.math (ProteinShader API)


|  |  |  |  |  |  |  |  |  |  |  |
| --- | --- | --- | --- | --- | --- | --- | --- | --- | --- | --- |
| |  |  |  |  |  |  |  |  | | --- | --- | --- | --- | --- | --- | --- | --- | | **Overview** | **Package** | Class | **Use** | **Tree** | **Deprecated** | **Index** | **Help** | | |  |
| PREV   NEXT | **FRAMES**    **NO FRAMES**     **All Classes** |


---


## **Uses of Package org.proteinshader.math**

| Packages that use org.proteinshader.math | |
| --- | --- |
| **org.proteinshader.graphics** | Holds the drawing classes: Ribbon, Tube, FrenetFrames, Sphere, and Cylinder. |
| **org.proteinshader.gui.viewing** | Holds important helper classes that the Renderer uses to control the view: Camera, Rotation, Lighting, *etc*. |
| **org.proteinshader.math** | The key classes in this package are Hermite and Quaternion, which are needed for generating the ribbons and tubes that are used to represent the backbone of a protein in a cartoon-type display. |
| **org.proteinshader.structure** | Holds the classes that store information from a Protein Data Bank file: Structure, Model, Chain, AminoAcid, Heterogen, Water, Atom, Bond, Helix, BetaStrand, Loop, *etc*. |
| **org.proteinshader.structure.io** | Holds the classes needed for reading Protein Data Bank structure files. |
| **org.proteinshader.structure.visitor** | Holds Visitor classes that know how to traverse the hierarchy of objects held by class Structure. |

| Classes in org.proteinshader.math used by org.proteinshader.graphics | |
| --- | --- |
| ****LocalFrame****             Stores a local coordinate frame as a rotation (a Quaternion) and a translation (a Point3d). |

| Classes in org.proteinshader.math used by org.proteinshader.gui.viewing | |
| --- | --- |
| ****Vec3d****             This class is used to create a vector with 3 elements of type double. |

| Classes in org.proteinshader.math used by org.proteinshader.math | |
| --- | --- |
| ****Hermite****             Calculates a cubic equation between two control points so that points on the curve in between can be interpolated. |
| ****LocalFrame****             Stores a local coordinate frame as a rotation (a Quaternion) and a translation (a Point3d). |
| ****Point3d****             This class is used to create a point with 3 elements of type double. |
| ****Quaternion****             This class is used to create a quaternion, a four-dimensional complex number that is typically used to represent a rotation in three-dimensional space. |
| ****Vec3d****             This class is used to create a vector with 3 elements of type double. |

| Classes in org.proteinshader.math used by org.proteinshader.structure | |
| --- | --- |
| ****Hermite****             Calculates a cubic equation between two control points so that points on the curve in between can be interpolated. |
| ****LocalFrame****             Stores a local coordinate frame as a rotation (a Quaternion) and a translation (a Point3d). |
| ****Point3d****             This class is used to create a point with 3 elements of type double. |
| ****Quaternion****             This class is used to create a quaternion, a four-dimensional complex number that is typically used to represent a rotation in three-dimensional space. |
| ****Vec3d****             This class is used to create a vector with 3 elements of type double. |

| Classes in org.proteinshader.math used by org.proteinshader.structure.io | |
| --- | --- |
| ****LocalFrame****             Stores a local coordinate frame as a rotation (a Quaternion) and a translation (a Point3d). |

| Classes in org.proteinshader.math used by org.proteinshader.structure.visitor | |
| --- | --- |
| ****Quaternion****             This class is used to create a quaternion, a four-dimensional complex number that is typically used to represent a rotation in three-dimensional space. |

---


|  |  |  |  |  |  |  |  |  |  |  |
| --- | --- | --- | --- | --- | --- | --- | --- | --- | --- | --- |
| |  |  |  |  |  |  |  |  | | --- | --- | --- | --- | --- | --- | --- | --- | | **Overview** | **Package** | Class | **Use** | **Tree** | **Deprecated** | **Index** | **Help** | | |  |
| PREV   NEXT | **FRAMES**    **NO FRAMES**     **All Classes** |


---

# *Copyright © 2007-2008*
