# Supplementary material for: ProteinShader: illustrative rendering of macromolecules
Source: BMC Struct Biol. 2009 Mar 30;9:19. doi: 10.1186/1472-6807-9-19 (PMC2672931; doi:10.1186/1472-6807-9-19)
Supplement: Additional file 1 — ProteinShader program without source code. This compressed file contains the complete ProteinShader program including associated libraries, but no source code. A README.txt file gives an overview of the ProteinShader distribution, and the index.html file in the help subdirectory has directions on getting started with the program as well as a set of tutorials. [file 1472-6807-9-19-S1.zip › ProteinShader-beta-0_9_4-binary/help/api/org/proteinshader/graphics/utils/SegmentRenderer.html]

SegmentRenderer (ProteinShader API)


|  |  |  |  |  |  |  |  |  |  |  |
| --- | --- | --- | --- | --- | --- | --- | --- | --- | --- | --- |
| |  |  |  |  |  |  |  |  | | --- | --- | --- | --- | --- | --- | --- | --- | | **Overview** | **Package** | **Class** | **Use** | **Tree** | **Deprecated** | **Index** | **Help** | | |  |
| PREV CLASS   NEXT CLASS | **FRAMES**    **NO FRAMES**     **All Classes** |
| SUMMARY: NESTED | FIELD | CONSTR | METHOD | DETAIL: FIELD | CONSTR | METHOD |


---


## org.proteinshader.graphics.utils Class SegmentRenderer

```
java.lang.Object
  org.proteinshader.graphics.utils.SegmentRenderer
```

---

``` public class SegmentRenderer extends Object ```

This class is intended only as a testing utility: it allows tube or ribbons
segments to be drawn on-the-fly, rather than from OpenGL display lists, soley
for the purpose of measure the performance advantage of caching geometry in
OpenGL display lists.

---

| **Constructor Summary** | |
| --- | --- |
| `SegmentRenderer()`             Constructs a SegmentReferences object. |


| **Method Summary** | |
| --- | --- |
| `void` | `drawEndCap(GL gl)`             Draws the end cap for the last tube or ribbon segment that was drawn. |
| `void` | `drawRibbon(GL gl, Segment s)`             Draws the broad surfaces of a ribbon segment and remembers info needed by any subsequent call to drawThinSidesOfRibbon(), drawStartCap(), or drawEndCap(). |
| `void` | `drawStartCap(GL gl)`             Draws the start cap for the last tube or ribbon segment that was drawn. |
| `void` | `drawThinSidesOfRibbon(GL gl)`             Draws the thin sides for the last ribbon segment that was drawn. |
| `void` | `drawTube(GL gl, Segment s)`             Draws the curved body of a tube segment and remembers info needed by any subsequent call to drawStartCap() or drawEndCap(). |
| `void` | `setAllStackNumbersToDefault()`             Sets all stacks numbers to the defaults defined in classes Tube and Ribbon (for loops, alpha-helices, and beta-strands). |
| `void` | `setAlphaRibbonStacks(int stacks)`             Sets the number of stacks for drawing an alpha-helix as a ribbon. |
| `void` | `setAlphaRibbonStacksToDefault()`             Sets the number of stacks for drawing an alpha-helix as a ribbon to the default value defined in class Ribbon. |
| `void` | `setAlphaTubeStacks(int stacks)`             Sets the number of stacks for drawing an alpha-helix as a tube. |
| `void` | `setAlphaTubeStacksToDefault()`             Sets the number of stacks for drawing an alpha-helix as a tube to the default value defined in class Tube. |
| `void` | `setBetaRibbonStacks(int stacks)`             Sets the number of stacks for drawing a beta-strand as a ribbon. |
| `void` | `setBetaRibbonStacksToDefault()`             Sets the number of stacks for drawing a beta-strand as a ribbon to the default value defined in class Ribbon. |
| `void` | `setBetaTubeStacks(int stacks)`             Sets the number of stacks for drawing a beta-strand as a tube. |
| `void` | `setBetaTubeStacksToDefault()`             Sets the number of stacks for drawing a beta-strand as a tube to the default value defined in class Tube. |
| `void` | `setLoopRibbonStacks(int stacks)`             Sets the number of stacks for drawing a loop as a ribbon. |
| `void` | `setLoopRibbonStacksToDefault()`             Sets the number of stacks for drawing a loop as a ribbon to the default value defined in class Ribbon. |
| `void` | `setLoopTubeStacks(int stacks)`             Sets the number of stacks for drawing a loop as a tube. |
| `void` | `setLoopTubeStacksToDefault()`             Sets the number of stacks for drawing a loop as a tube to the default value defined in class Tube. |

| **Methods inherited from class java.lang.Object** |
| --- |
| `clone, equals, finalize, getClass, hashCode, notify, notifyAll, toString, wait, wait, wait` |

| **Constructor Detail** |
| --- |

### SegmentRenderer

```
public SegmentRenderer()
```

:   Constructs a SegmentReferences object.


| **Method Detail** |
| --- |

### drawTube

```
public void drawTube(GL gl,
                     Segment s)
```

:   Draws the curved body of a tube segment and remembers info needed
    by any subsequent call to drawStartCap() or drawEndCap().

    :   **Parameters:**: `gl` - the current GL object.: `s` - the segment to draw.

---


### drawStartCap

```
public void drawStartCap(GL gl)
```

:   Draws the start cap for the last tube or ribbon segment that was
    drawn.

    :   **Parameters:**: `gl` - the current GL object.

---


### drawEndCap

```
public void drawEndCap(GL gl)
```

:   Draws the end cap for the last tube or ribbon segment that was
    drawn.

    :   **Parameters:**: `gl` - the current GL object.

---


### drawRibbon

```
public void drawRibbon(GL gl,
                       Segment s)
```

:   Draws the broad surfaces of a ribbon segment and remembers info
    needed by any subsequent call to drawThinSidesOfRibbon(),
    drawStartCap(), or drawEndCap().

    :   **Parameters:**: `gl` - the current GL object.: `s` - the segment to draw.

---


### drawThinSidesOfRibbon

```
public void drawThinSidesOfRibbon(GL gl)
```

:   Draws the thin sides for the last ribbon segment that was drawn.

    :   **Parameters:**: `gl` - the current GL object.

---


### setAllStackNumbersToDefault

```
public void setAllStackNumbersToDefault()
```

:   Sets all stacks numbers to the defaults defined in classes Tube
    and Ribbon (for loops, alpha-helices, and beta-strands).

---


### setLoopTubeStacksToDefault

```
public void setLoopTubeStacksToDefault()
```

:   Sets the number of stacks for drawing a loop as a tube to the
    default value defined in class Tube.
    After setting this value, cacheSegmentGeometry() still needs to be
    called with the current GL object for the change to take effect.

---


### setAlphaTubeStacksToDefault

```
public void setAlphaTubeStacksToDefault()
```

:   Sets the number of stacks for drawing an alpha-helix as a tube to
    the default value defined in class Tube.
    After setting this value, cacheSegmentGeometry() still needs to be
    called with the current GL object for the change to take effect.

---


### setBetaTubeStacksToDefault

```
public void setBetaTubeStacksToDefault()
```

:   Sets the number of stacks for drawing a beta-strand as a tube to
    the default value defined in class Tube.
    After setting this value, cacheSegmentGeometry() still needs to be
    called with the current GL object for the change to take effect.

---


### setLoopRibbonStacksToDefault

```
public void setLoopRibbonStacksToDefault()
```

:   Sets the number of stacks for drawing a loop as a ribbon to
    the default value defined in class Ribbon.
    After setting this value, cacheSegmentGeometry() still needs to be
    called with the current GL object for the change to take effect.

---


### setAlphaRibbonStacksToDefault

```
public void setAlphaRibbonStacksToDefault()
```

:   Sets the number of stacks for drawing an alpha-helix as a ribbon
    to the default value defined in class Ribbon.
    After setting this value, cacheSegmentGeometry() still needs to be
    called with the current GL object for the change to take effect.

---


### setBetaRibbonStacksToDefault

```
public void setBetaRibbonStacksToDefault()
```

:   Sets the number of stacks for drawing a beta-strand as a ribbon to
    the default value defined in class Ribbon.
    After setting this value, cacheSegmentGeometry() still needs to be
    called with the current GL object for the change to take effect.

---


### setLoopTubeStacks

```
public void setLoopTubeStacks(int stacks)
```

:   Sets the number of stacks for drawing a loop as a tube.
    If the argument is less than 1, the number of stacks will be set
    to 1.
    After setting this value, cacheSegmentGeometry() still needs to be
    called with the current GL object for the change to take effect.

    :   **Parameters:**: `stacks` - the number of stacks.

---


### setAlphaTubeStacks

```
public void setAlphaTubeStacks(int stacks)
```

:   Sets the number of stacks for drawing an alpha-helix as a tube.
    If the argument is less than 1, the number of stacks will be set
    to 1.
    After setting this value, cacheSegmentGeometry() still needs to be
    called with the current GL object for the change to take effect.

    :   **Parameters:**: `stacks` - the number of stacks.

---


### setBetaTubeStacks

```
public void setBetaTubeStacks(int stacks)
```

:   Sets the number of stacks for drawing a beta-strand as a tube.
    If the argument is less than 1, the number of stacks will be set
    to 1.
    After setting this value, cacheSegmentGeometry() still needs to be
    called with the current GL object for the change to take effect.

    :   **Parameters:**: `stacks` - the number of stacks.

---


### setLoopRibbonStacks

```
public void setLoopRibbonStacks(int stacks)
```

:   Sets the number of stacks for drawing a loop as a ribbon.
    If the argument is less than 1, the number of stacks will be set
    to 1.
    After setting this value, cacheSegmentGeometry() still needs to be
    called with the current GL object for the change to take effect.

    :   **Parameters:**: `stacks` - the number of stacks.

---


### setAlphaRibbonStacks

```
public void setAlphaRibbonStacks(int stacks)
```

:   Sets the number of stacks for drawing an alpha-helix as a ribbon.
    If the argument is less than 1, the number of stacks will be set
    to 1.
    After setting this value, cacheSegmentGeometry() still needs to be
    called with the current GL object for the change to take effect.

    :   **Parameters:**: `stacks` - the number of stacks.

---


### setBetaRibbonStacks

```
public void setBetaRibbonStacks(int stacks)
```

:   Sets the number of stacks for drawing a beta-strand as a ribbon.
    If the argument is less than 1, the number of stacks will be set
    to 1.
    After setting this value, cacheSegmentGeometry() still needs to be
    called with the current GL object for the change to take effect.

    :   **Parameters:**: `stacks` - the number of stacks.


---


|  |  |  |  |  |  |  |  |  |  |  |
| --- | --- | --- | --- | --- | --- | --- | --- | --- | --- | --- |
| |  |  |  |  |  |  |  |  | | --- | --- | --- | --- | --- | --- | --- | --- | | **Overview** | **Package** | **Class** | **Use** | **Tree** | **Deprecated** | **Index** | **Help** | | |  |
| PREV CLASS   NEXT CLASS | **FRAMES**    **NO FRAMES**     **All Classes** |
| SUMMARY: NESTED | FIELD | CONSTR | METHOD | DETAIL: FIELD | CONSTR | METHOD |


---

# *Copyright © 2007-2008*
