# Supplementary material for: ProteinShader: illustrative rendering of macromolecules
Source: BMC Struct Biol. 2009 Mar 30;9:19. doi: 10.1186/1472-6807-9-19 (PMC2672931; doi:10.1186/1472-6807-9-19)
Supplement: Additional file 1 — ProteinShader program without source code. This compressed file contains the complete ProteinShader program including associated libraries, but no source code. A README.txt file gives an overview of the ProteinShader distribution, and the index.html file in the help subdirectory has directions on getting started with the program as well as a set of tutorials. [file 1472-6807-9-19-S1.zip › ProteinShader-beta-0_9_4-binary/help/api/org/proteinshader/graphics/adapter/class-use/StructureToGraphics.html]

Uses of Class org.proteinshader.graphics.adapter.StructureToGraphics (ProteinShader API)


|  |  |  |  |  |  |  |  |  |  |  |
| --- | --- | --- | --- | --- | --- | --- | --- | --- | --- | --- |
| |  |  |  |  |  |  |  |  | | --- | --- | --- | --- | --- | --- | --- | --- | | **Overview** | **Package** | **Class** | **Use** | **Tree** | **Deprecated** | **Index** | **Help** | | |  |
| PREV   NEXT | **FRAMES**    **NO FRAMES**     **All Classes** |


---


## **Uses of Class org.proteinshader.graphics.adapter.StructureToGraphics**

| Packages that use StructureToGraphics | |
| --- | --- |
| **org.proteinshader.gui** | Holds all of the Swing GUI components and their associated listeners, including class Renderer, which is registered as a listener for the GLCanvas object that is used a drawing surface. |
| **org.proteinshader.gui.utils** | Holds minor utility classes needed by the Renderer. |

| Uses of StructureToGraphics in org.proteinshader.gui | |
| --- | --- |

| Methods in org.proteinshader.gui that return StructureToGraphics | |
| --- | --- |
| `StructureToGraphics` | `Renderer.getGraphics()`             Returns a reference to the StructureToGraphics object used by the Renderer. |

| Uses of StructureToGraphics in org.proteinshader.gui.utils | |
| --- | --- |

| Constructors in org.proteinshader.gui.utils with parameters of type StructureToGraphics | |
| --- | --- |
| `FpsLabel(StructureToGraphics graphics)`             Constructs an FpsLabel object. |

---


|  |  |  |  |  |  |  |  |  |  |  |
| --- | --- | --- | --- | --- | --- | --- | --- | --- | --- | --- |
| |  |  |  |  |  |  |  |  | | --- | --- | --- | --- | --- | --- | --- | --- | | **Overview** | **Package** | **Class** | **Use** | **Tree** | **Deprecated** | **Index** | **Help** | | |  |
| PREV   NEXT | **FRAMES**    **NO FRAMES**     **All Classes** |


---

# *Copyright © 2007-2008*
