# Supplementary material for: ProteinShader: illustrative rendering of macromolecules
Source: BMC Struct Biol. 2009 Mar 30;9:19. doi: 10.1186/1472-6807-9-19 (PMC2672931; doi:10.1186/1472-6807-9-19)
Supplement: Additional file 1 — ProteinShader program without source code. This compressed file contains the complete ProteinShader program including associated libraries, but no source code. A README.txt file gives an overview of the ProteinShader distribution, and the index.html file in the help subdirectory has directions on getting started with the program as well as a set of tutorials. [file 1472-6807-9-19-S1.zip › ProteinShader-beta-0_9_4-binary/help/api/org/proteinshader/graphics/adapter/package-summary.html]

org.proteinshader.graphics.adapter (ProteinShader API)


|  |  |  |  |  |  |  |  |  |  |  |
| --- | --- | --- | --- | --- | --- | --- | --- | --- | --- | --- |
| |  |  |  |  |  |  |  |  | | --- | --- | --- | --- | --- | --- | --- | --- | | **Overview** | **Package** | Class | **Use** | **Tree** | **Deprecated** | **Index** | **Help** | | |  |
| **PREV PACKAGE**   **NEXT PACKAGE** | **FRAMES**    **NO FRAMES**     **All Classes** |


---

## Package org.proteinshader.graphics.adapter

Holds the StructureToGraphics class, which is used to manage the use of the
drawing classes and OpenGL display lists.

**See:**
  
          **Description**

| **Class Summary** | |
| --- | --- |
| **StructureToGraphics** | Knows how to get numbers (such as zyz-coordinates and colors) from a Drawable object (Atom, Bond, or Segment) and plug them into the right graphics class for rendering a Shape (Sphere, Cylinder, or Segment). |

## Package org.proteinshader.graphics.adapter Description

Holds the StructureToGraphics class, which is used to manage the use of the
drawing classes and OpenGL display lists.
