# Supplementary material for: ProteinShader: illustrative rendering of macromolecules
Source: BMC Struct Biol. 2009 Mar 30;9:19. doi: 10.1186/1472-6807-9-19 (PMC2672931; doi:10.1186/1472-6807-9-19)
Supplement: Additional file 1 — ProteinShader program without source code. This compressed file contains the complete ProteinShader program including associated libraries, but no source code. A README.txt file gives an overview of the ProteinShader distribution, and the index.html file in the help subdirectory has directions on getting started with the program as well as a set of tutorials. [file 1472-6807-9-19-S1.zip › ProteinShader-beta-0_9_4-binary/help/api/org/proteinshader/graphics/class-use/Shape.html]

Uses of Class org.proteinshader.graphics.Shape (ProteinShader API)


|  |  |  |  |  |  |  |  |  |  |  |
| --- | --- | --- | --- | --- | --- | --- | --- | --- | --- | --- |
| |  |  |  |  |  |  |  |  | | --- | --- | --- | --- | --- | --- | --- | --- | | **Overview** | **Package** | **Class** | **Use** | **Tree** | **Deprecated** | **Index** | **Help** | | |  |
| PREV   NEXT | **FRAMES**    **NO FRAMES**     **All Classes** |


---


## **Uses of Class org.proteinshader.graphics.Shape**

| Packages that use Shape | |
| --- | --- |
| **org.proteinshader.graphics** | Holds the drawing classes: Ribbon, Tube, FrenetFrames, Sphere, and Cylinder. |

| Uses of Shape in org.proteinshader.graphics | |
| --- | --- |

| Subclasses of Shape in org.proteinshader.graphics | |
| --- | --- |
| `class` | `Cylinder`             Knows how to draw a simple cylinder useful for representing the bonds between atoms. |
| `class` | `ExtrudedShape`             Contains common methods needed by classes that draw segments of a three-dimensional tube by sweeping a waist polygon along a spline. |
| `class` | `FrenetFrames`             Provides a visual representation of the LocalFrame objects of a Segment by drawing an x-axis, a y-axis, and z-axis. |
| `class` | `Ribbon`             Draws a segment of a three-dimensional ribbon by using a rectangular-shaped waist polygon. |
| `class` | `Sphere`             Knows how to draw a sphere with texture coordinates, a surface normal, and a tangent vector for each vertex. |
| `class` | `Tube`             Draws a segment of a three-dimensional tube by using a waist polygon that approximates a circle as the number of vertices in the waist polygon becomes large. |

---


|  |  |  |  |  |  |  |  |  |  |  |
| --- | --- | --- | --- | --- | --- | --- | --- | --- | --- | --- |
| |  |  |  |  |  |  |  |  | | --- | --- | --- | --- | --- | --- | --- | --- | | **Overview** | **Package** | **Class** | **Use** | **Tree** | **Deprecated** | **Index** | **Help** | | |  |
| PREV   NEXT | **FRAMES**    **NO FRAMES**     **All Classes** |


---

# *Copyright © 2007-2008*
