# Supplementary material for: ProteinShader: illustrative rendering of macromolecules
Source: BMC Struct Biol. 2009 Mar 30;9:19. doi: 10.1186/1472-6807-9-19 (PMC2672931; doi:10.1186/1472-6807-9-19)
Supplement: Additional file 1 — ProteinShader program without source code. This compressed file contains the complete ProteinShader program including associated libraries, but no source code. A README.txt file gives an overview of the ProteinShader distribution, and the index.html file in the help subdirectory has directions on getting started with the program as well as a set of tutorials. [file 1472-6807-9-19-S1.zip › ProteinShader-beta-0_9_4-binary/help/api/org/proteinshader/graphics/class-use/Cylinder.html]

Uses of Class org.proteinshader.graphics.Cylinder (ProteinShader API)


|  |  |  |  |  |  |  |  |  |  |  |
| --- | --- | --- | --- | --- | --- | --- | --- | --- | --- | --- |
| |  |  |  |  |  |  |  |  | | --- | --- | --- | --- | --- | --- | --- | --- | | **Overview** | **Package** | **Class** | **Use** | **Tree** | **Deprecated** | **Index** | **Help** | | |  |
| PREV   NEXT | **FRAMES**    **NO FRAMES**     **All Classes** |


---


## **Uses of Class org.proteinshader.graphics.Cylinder**

| Packages that use Cylinder | |
| --- | --- |
| **org.proteinshader.graphics.displaylists** | Holds the classes needed to manage OpenGL display lists, which are used to cache reusable geometry for spheres, cylinders, ribbon segments, and tube segments. |

| Uses of Cylinder in org.proteinshader.graphics.displaylists | |
| --- | --- |

| Methods in org.proteinshader.graphics.displaylists with parameters of type Cylinder | |
| --- | --- |
| `void` | `CylinderReferences.cacheCylinderDisplayList(GL gl, Cylinder cylinder, CylinderListInfo info)`             Caches a new OpenGL display list for a BALLS\_AND\_STICKS cylinder or a STICKS cylinder with the requested number of slices and stacks. |
| `void` | `CylinderReferences.cacheDefaultCylinders(GL gl, Cylinder cylinder)`             Caches a collection of cylinders using OpenGL display lists. |

---


|  |  |  |  |  |  |  |  |  |  |  |
| --- | --- | --- | --- | --- | --- | --- | --- | --- | --- | --- |
| |  |  |  |  |  |  |  |  | | --- | --- | --- | --- | --- | --- | --- | --- | | **Overview** | **Package** | **Class** | **Use** | **Tree** | **Deprecated** | **Index** | **Help** | | |  |
| PREV   NEXT | **FRAMES**    **NO FRAMES**     **All Classes** |


---

# *Copyright © 2007-2008*
