# Supplementary material for: ProteinShader: illustrative rendering of macromolecules
Source: BMC Struct Biol. 2009 Mar 30;9:19. doi: 10.1186/1472-6807-9-19 (PMC2672931; doi:10.1186/1472-6807-9-19)
Supplement: Additional file 1 — ProteinShader program without source code. This compressed file contains the complete ProteinShader program including associated libraries, but no source code. A README.txt file gives an overview of the ProteinShader distribution, and the index.html file in the help subdirectory has directions on getting started with the program as well as a set of tutorials. [file 1472-6807-9-19-S1.zip › ProteinShader-beta-0_9_4-binary/help/api/org/proteinshader/graphics/displaylists/class-use/SphereListInfo.html]

Uses of Class org.proteinshader.graphics.displaylists.SphereListInfo (ProteinShader API)


|  |  |  |  |  |  |  |  |  |  |  |
| --- | --- | --- | --- | --- | --- | --- | --- | --- | --- | --- |
| |  |  |  |  |  |  |  |  | | --- | --- | --- | --- | --- | --- | --- | --- | | **Overview** | **Package** | **Class** | **Use** | **Tree** | **Deprecated** | **Index** | **Help** | | |  |
| PREV   NEXT | **FRAMES**    **NO FRAMES**     **All Classes** |


---


## **Uses of Class org.proteinshader.graphics.displaylists.SphereListInfo**

| Packages that use SphereListInfo | |
| --- | --- |
| **org.proteinshader.graphics** | Holds the drawing classes: Ribbon, Tube, FrenetFrames, Sphere, and Cylinder. |
| **org.proteinshader.graphics.adapter** | Holds the StructureToGraphics class, which is used to manage the use of the drawing classes and OpenGL display lists. |
| **org.proteinshader.graphics.displaylists** | Holds the classes needed to manage OpenGL display lists, which are used to cache reusable geometry for spheres, cylinders, ribbon segments, and tube segments. |
| **org.proteinshader.gui** | Holds all of the Swing GUI components and their associated listeners, including class Renderer, which is registered as a listener for the GLCanvas object that is used a drawing surface. |

| Uses of SphereListInfo in org.proteinshader.graphics | |
| --- | --- |

| Methods in org.proteinshader.graphics that return SphereListInfo | |
| --- | --- |
| `SphereListInfo` | `Sphere.createDisplayList(GL gl, SphereListInfo info)`             Creates an OpenGL display list for drawing a sphere with the radius, number of slices, and number of stacks specified in the SphereListInfo object given as an argument. |

| Methods in org.proteinshader.graphics with parameters of type SphereListInfo | |
| --- | --- |
| `SphereListInfo` | `Sphere.createDisplayList(GL gl, SphereListInfo info)`             Creates an OpenGL display list for drawing a sphere with the radius, number of slices, and number of stacks specified in the SphereListInfo object given as an argument. |

| Uses of SphereListInfo in org.proteinshader.graphics.adapter | |
| --- | --- |

| Methods in org.proteinshader.graphics.adapter that return SphereListInfo | |
| --- | --- |
| `SphereListInfo` | `StructureToGraphics.getSphereInfo(StyleEnum style)`             Returns the SphereListInfo object that holds the information on an OpenGL display list for a sphere that is currently used for drawing Atoms. |

| Uses of SphereListInfo in org.proteinshader.graphics.displaylists | |
| --- | --- |

| Methods in org.proteinshader.graphics.displaylists that return SphereListInfo | |
| --- | --- |
| `SphereListInfo` | `SphereReferences.getBallsAndSticksSphereInfo()`             Returns the SphereListInfo object that holds the information on an OpenGL display list for a sphere to be used in a BALLS\_AND\_STICKS style display. |
| `SphereListInfo` | `SphereReferences.getSpaceFillingSphereInfo()`             Returns the SphereListInfo object that holds the information on an OpenGL display list for a sphere to be used for SPACE\_FILLING style. |

| Methods in org.proteinshader.graphics.displaylists with parameters of type SphereListInfo | |
| --- | --- |
| `void` | `SphereReferences.cacheSphereDisplayList(GL gl, Sphere sphere, SphereListInfo info)`             Caches a new OpenGL display list for a SPACE\_FILLING sphere or a BALLS\_AND\_STICKS sphere with the requested tiling (number of slices and stacks). |

| Uses of SphereListInfo in org.proteinshader.gui | |
| --- | --- |

| Methods in org.proteinshader.gui that return SphereListInfo | |
| --- | --- |
| `SphereListInfo` | `MediatorImpl.getSphereInfo(StyleEnum style)`             Returns the SphereListInfo object that holds the information on an OpenGL display list for a sphere that is currently used for drawing Atoms. |
| `SphereListInfo` | `Mediator.getSphereInfo(StyleEnum style)`             Returns the SphereListInfo object that holds the information on an OpenGL display list for a sphere that is currently used for drawing Atoms. |

---


|  |  |  |  |  |  |  |  |  |  |  |
| --- | --- | --- | --- | --- | --- | --- | --- | --- | --- | --- |
| |  |  |  |  |  |  |  |  | | --- | --- | --- | --- | --- | --- | --- | --- | | **Overview** | **Package** | **Class** | **Use** | **Tree** | **Deprecated** | **Index** | **Help** | | |  |
| PREV   NEXT | **FRAMES**    **NO FRAMES**     **All Classes** |


---

# *Copyright © 2007-2008*
