# Supplementary material for: ProteinShader: illustrative rendering of macromolecules
Source: BMC Struct Biol. 2009 Mar 30;9:19. doi: 10.1186/1472-6807-9-19 (PMC2672931; doi:10.1186/1472-6807-9-19)
Supplement: Additional file 1 — ProteinShader program without source code. This compressed file contains the complete ProteinShader program including associated libraries, but no source code. A README.txt file gives an overview of the ProteinShader distribution, and the index.html file in the help subdirectory has directions on getting started with the program as well as a set of tutorials. [file 1472-6807-9-19-S1.zip › ProteinShader-beta-0_9_4-binary/help/api/org/proteinshader/graphics/displaylists/class-use/GeometricListInfo.html]

Uses of Class org.proteinshader.graphics.displaylists.GeometricListInfo (ProteinShader API)


|  |  |  |  |  |  |  |  |  |  |  |
| --- | --- | --- | --- | --- | --- | --- | --- | --- | --- | --- |
| |  |  |  |  |  |  |  |  | | --- | --- | --- | --- | --- | --- | --- | --- | | **Overview** | **Package** | **Class** | **Use** | **Tree** | **Deprecated** | **Index** | **Help** | | |  |
| PREV   NEXT | **FRAMES**    **NO FRAMES**     **All Classes** |


---


## **Uses of Class org.proteinshader.graphics.displaylists.GeometricListInfo**

| Packages that use GeometricListInfo | |
| --- | --- |
| **org.proteinshader.graphics.adapter** | Holds the StructureToGraphics class, which is used to manage the use of the drawing classes and OpenGL display lists. |
| **org.proteinshader.graphics.displaylists** | Holds the classes needed to manage OpenGL display lists, which are used to cache reusable geometry for spheres, cylinders, ribbon segments, and tube segments. |
| **org.proteinshader.gui** | Holds all of the Swing GUI components and their associated listeners, including class Renderer, which is registered as a listener for the GLCanvas object that is used a drawing surface. |

| Uses of GeometricListInfo in org.proteinshader.graphics.adapter | |
| --- | --- |

| Methods in org.proteinshader.graphics.adapter with parameters of type GeometricListInfo | |
| --- | --- |
| `void` | `StructureToGraphics.cacheGeometricObject(GL gl, GeometricListInfo info)`             Caches a new OpenGL display list for a geometric object. |

| Uses of GeometricListInfo in org.proteinshader.graphics.displaylists | |
| --- | --- |

| Subclasses of GeometricListInfo in org.proteinshader.graphics.displaylists | |
| --- | --- |
| `class` | `CylinderListInfo`             Stores information on an OpenGL display list for a cylinder. |
| `class` | `SegmentListInfo`             Stores information on an OpenGL display list for a Segment. |
| `class` | `SphereListInfo`             Stores information on an OpenGL display list for a sphere. |

| Uses of GeometricListInfo in org.proteinshader.gui | |
| --- | --- |

| Methods in org.proteinshader.gui with parameters of type GeometricListInfo | |
| --- | --- |
| `void` | `MediatorImpl.cacheGeometricObject(GeometricListInfo info)`             Caches a new geomtric object (sphere or cylinder) in the form of an OpenGL display list and redraws the canvas. |
| `void` | `Renderer.cacheGeometricObject(GeometricListInfo info)`             Stores a request to cache a new geomtric object so that the request can be processed the next time display() is called. |
| `void` | `Mediator.cacheGeometricObject(GeometricListInfo info)`             Caches a new geomtric object (sphere or cylinder) in the form of an OpenGL display list and redraws the canvas. |

---


|  |  |  |  |  |  |  |  |  |  |  |
| --- | --- | --- | --- | --- | --- | --- | --- | --- | --- | --- |
| |  |  |  |  |  |  |  |  | | --- | --- | --- | --- | --- | --- | --- | --- | | **Overview** | **Package** | **Class** | **Use** | **Tree** | **Deprecated** | **Index** | **Help** | | |  |
| PREV   NEXT | **FRAMES**    **NO FRAMES**     **All Classes** |


---

# *Copyright © 2007-2008*
