# Supplementary material for: ProteinShader: illustrative rendering of macromolecules
Source: BMC Struct Biol. 2009 Mar 30;9:19. doi: 10.1186/1472-6807-9-19 (PMC2672931; doi:10.1186/1472-6807-9-19)
Supplement: Additional file 1 — ProteinShader program without source code. This compressed file contains the complete ProteinShader program including associated libraries, but no source code. A README.txt file gives an overview of the ProteinShader distribution, and the index.html file in the help subdirectory has directions on getting started with the program as well as a set of tutorials. [file 1472-6807-9-19-S1.zip › ProteinShader-beta-0_9_4-binary/help/api/org/proteinshader/graphics/displaylists/class-use/SegmentListInfo.html]

Uses of Class org.proteinshader.graphics.displaylists.SegmentListInfo (ProteinShader API)


|  |  |  |  |  |  |  |  |  |  |  |
| --- | --- | --- | --- | --- | --- | --- | --- | --- | --- | --- |
| |  |  |  |  |  |  |  |  | | --- | --- | --- | --- | --- | --- | --- | --- | | **Overview** | **Package** | **Class** | **Use** | **Tree** | **Deprecated** | **Index** | **Help** | | |  |
| PREV   NEXT | **FRAMES**    **NO FRAMES**     **All Classes** |


---


## **Uses of Class org.proteinshader.graphics.displaylists.SegmentListInfo**

| Packages that use SegmentListInfo | |
| --- | --- |
| **org.proteinshader.graphics** | Holds the drawing classes: Ribbon, Tube, FrenetFrames, Sphere, and Cylinder. |
| **org.proteinshader.graphics.displaylists** | Holds the classes needed to manage OpenGL display lists, which are used to cache reusable geometry for spheres, cylinders, ribbon segments, and tube segments. |

| Uses of SegmentListInfo in org.proteinshader.graphics | |
| --- | --- |

| Methods in org.proteinshader.graphics with parameters of type SegmentListInfo | |
| --- | --- |
| `void` | `Ribbon.createDisplayLists(GL gl, LocalFrame[] frames, SegmentListInfo info)`             Creates an OpenGL display list for the thin sides of the ribbon, and also calls on the createDisplayLists() method of superclass ExtrudedShape to save the OpenGL display lists for the main (broad surface) part of the ribbon and for the start and end caps. |
| `void` | `ExtrudedShape.createDisplayLists(GL gl, LocalFrame[] frames, SegmentListInfo info)`             Creates three OpenGL display lists: one created by calling draw(), one created by calling drawStartCap(), and one created by calling drawEndCap(). |
| `void` | `Silhouette.drawRibbon(GL gl, Segment s, SegmentListInfo info, boolean extraLines)`             Draws a translucent black silhoutte of a ribbon segment using jitter. |
| `void` | `Silhouette.drawTube(GL gl, Segment s, SegmentListInfo info, boolean extraLines)`             Draws a translucent black silhoutte of a tube segment using jitter. |

| Uses of SegmentListInfo in org.proteinshader.graphics.displaylists | |
| --- | --- |

| Methods in org.proteinshader.graphics.displaylists that return SegmentListInfo | |
| --- | --- |
| `SegmentListInfo` | `SegmentReferences.getSegmentListInfo(Segment segment)`             Returns the SegmentListInfo object that stores information on the OpenGL display lists that have been cached for the Segment given as an argument. |

---


|  |  |  |  |  |  |  |  |  |  |  |
| --- | --- | --- | --- | --- | --- | --- | --- | --- | --- | --- |
| |  |  |  |  |  |  |  |  | | --- | --- | --- | --- | --- | --- | --- | --- | | **Overview** | **Package** | **Class** | **Use** | **Tree** | **Deprecated** | **Index** | **Help** | | |  |
| PREV   NEXT | **FRAMES**    **NO FRAMES**     **All Classes** |


---

# *Copyright © 2007-2008*
