# Supplementary material for: ProteinShader: illustrative rendering of macromolecules
Source: BMC Struct Biol. 2009 Mar 30;9:19. doi: 10.1186/1472-6807-9-19 (PMC2672931; doi:10.1186/1472-6807-9-19)
Supplement: Additional file 1 — ProteinShader program without source code. This compressed file contains the complete ProteinShader program including associated libraries, but no source code. A README.txt file gives an overview of the ProteinShader distribution, and the index.html file in the help subdirectory has directions on getting started with the program as well as a set of tutorials. [file 1472-6807-9-19-S1.zip › ProteinShader-beta-0_9_4-binary/help/api/org/proteinshader/graphics/displaylists/class-use/CylinderListInfo.html]

Uses of Class org.proteinshader.graphics.displaylists.CylinderListInfo (ProteinShader API)


|  |  |  |  |  |  |  |  |  |  |  |
| --- | --- | --- | --- | --- | --- | --- | --- | --- | --- | --- |
| |  |  |  |  |  |  |  |  | | --- | --- | --- | --- | --- | --- | --- | --- | | **Overview** | **Package** | **Class** | **Use** | **Tree** | **Deprecated** | **Index** | **Help** | | |  |
| PREV   NEXT | **FRAMES**    **NO FRAMES**     **All Classes** |


---


## **Uses of Class org.proteinshader.graphics.displaylists.CylinderListInfo**

| Packages that use CylinderListInfo | |
| --- | --- |
| **org.proteinshader.graphics** | Holds the drawing classes: Ribbon, Tube, FrenetFrames, Sphere, and Cylinder. |
| **org.proteinshader.graphics.adapter** | Holds the StructureToGraphics class, which is used to manage the use of the drawing classes and OpenGL display lists. |
| **org.proteinshader.graphics.displaylists** | Holds the classes needed to manage OpenGL display lists, which are used to cache reusable geometry for spheres, cylinders, ribbon segments, and tube segments. |
| **org.proteinshader.gui** | Holds all of the Swing GUI components and their associated listeners, including class Renderer, which is registered as a listener for the GLCanvas object that is used a drawing surface. |

| Uses of CylinderListInfo in org.proteinshader.graphics | |
| --- | --- |

| Methods in org.proteinshader.graphics that return CylinderListInfo | |
| --- | --- |
| `CylinderListInfo` | `Cylinder.createDisplayList(GL gl, CylinderListInfo info)`             Creates an OpenGL display list for drawing a cylinder with the radii, slices, stacks, and end caps specified in the CylinderListInfo object given as an argument. |

| Methods in org.proteinshader.graphics with parameters of type CylinderListInfo | |
| --- | --- |
| `CylinderListInfo` | `Cylinder.createDisplayList(GL gl, CylinderListInfo info)`             Creates an OpenGL display list for drawing a cylinder with the radii, slices, stacks, and end caps specified in the CylinderListInfo object given as an argument. |

| Uses of CylinderListInfo in org.proteinshader.graphics.adapter | |
| --- | --- |

| Methods in org.proteinshader.graphics.adapter that return CylinderListInfo | |
| --- | --- |
| `CylinderListInfo` | `StructureToGraphics.getCylinderInfo(StyleEnum style)`             Returns the CylinderListInfo object that holds the information on an OpenGL display list for a cylinder that is currently used for drawing Bonds. |

| Uses of CylinderListInfo in org.proteinshader.graphics.displaylists | |
| --- | --- |

| Methods in org.proteinshader.graphics.displaylists that return CylinderListInfo | |
| --- | --- |
| `CylinderListInfo` | `CylinderReferences.getBallsAndSticksCylinderInfo()`             Returns the CylinderListInfo object that holds the information on an OpenGL display list for a cylinder to be used for a BALLS\_AND\_STICKS style display. |
| `CylinderListInfo` | `CylinderReferences.getSticksCylinderInfo()`             Returns the CylinderListInfo object that holds the information on an OpenGL display list for a cylinder to be used for a STICKS style display. |

| Methods in org.proteinshader.graphics.displaylists with parameters of type CylinderListInfo | |
| --- | --- |
| `void` | `CylinderReferences.cacheCylinderDisplayList(GL gl, Cylinder cylinder, CylinderListInfo info)`             Caches a new OpenGL display list for a BALLS\_AND\_STICKS cylinder or a STICKS cylinder with the requested number of slices and stacks. |

| Uses of CylinderListInfo in org.proteinshader.gui | |
| --- | --- |

| Methods in org.proteinshader.gui that return CylinderListInfo | |
| --- | --- |
| `CylinderListInfo` | `MediatorImpl.getCylinderInfo(StyleEnum style)`             Returns the CylinderListInfo object that holds the information on an OpenGL display list for a cylinder that is currently used for drawing Bonds. |
| `CylinderListInfo` | `Mediator.getCylinderInfo(StyleEnum style)`             Returns the CylinderListInfo object that holds the information on an OpenGL display list for a cylinder that is currently used for drawing Bonds. |

---


|  |  |  |  |  |  |  |  |  |  |  |
| --- | --- | --- | --- | --- | --- | --- | --- | --- | --- | --- |
| |  |  |  |  |  |  |  |  | | --- | --- | --- | --- | --- | --- | --- | --- | | **Overview** | **Package** | **Class** | **Use** | **Tree** | **Deprecated** | **Index** | **Help** | | |  |
| PREV   NEXT | **FRAMES**    **NO FRAMES**     **All Classes** |


---

# *Copyright © 2007-2008*
