# Supplementary material for: ProteinShader: illustrative rendering of macromolecules
Source: BMC Struct Biol. 2009 Mar 30;9:19. doi: 10.1186/1472-6807-9-19 (PMC2672931; doi:10.1186/1472-6807-9-19)
Supplement: Additional file 1 — ProteinShader program without source code. This compressed file contains the complete ProteinShader program including associated libraries, but no source code. A README.txt file gives an overview of the ProteinShader distribution, and the index.html file in the help subdirectory has directions on getting started with the program as well as a set of tutorials. [file 1472-6807-9-19-S1.zip › ProteinShader-beta-0_9_4-binary/help/api/org/proteinshader/graphics/displaylists/CylinderReferences.html]

CylinderReferences (ProteinShader API)


|  |  |  |  |  |  |  |  |  |  |  |
| --- | --- | --- | --- | --- | --- | --- | --- | --- | --- | --- |
| |  |  |  |  |  |  |  |  | | --- | --- | --- | --- | --- | --- | --- | --- | | **Overview** | **Package** | **Class** | **Use** | **Tree** | **Deprecated** | **Index** | **Help** | | |  |
| **PREV CLASS**   **NEXT CLASS** | **FRAMES**    **NO FRAMES**     **All Classes** |
| SUMMARY: NESTED | FIELD | CONSTR | METHOD | DETAIL: FIELD | CONSTR | METHOD |


---


## org.proteinshader.graphics.displaylists Class CylinderReferences

```
java.lang.Object
  org.proteinshader.graphics.displaylists.CylinderReferences
```

---

``` public class CylinderReferences extends Object ```

Stores information on multiple OpenGL display lists that can be used
for rendering cylinders with different degrees of detail.

---

| **Field Summary** | |
| --- | --- |
| `static double` | `COEFFICIENT`             The empirically-determined coefficient for the power equation is 29.059. |
| `static int` | `DEFAULT_CAP_TILING`             The default tiling (number of slices and stacks) for a capping sphere is 5. |
| `static double` | `DEFAULT_HEIGHT`             The default cylinder height is 1.0. |
| `static double` | `DEFAULT_RADIUS`             Cylinders intended for a Sticks type of display will use a default cylinder radius is 0.15. |
| `static int` | `DEFAULT_SLICES`             The default number of slices for a cylinder is 10. |
| `static int` | `DEFAULT_STACKS`             The default number of stacks for a cylinder is 1. |
| `static double` | `EXPONENT`             The empirically-determined exponent for the power equation is -0.5267. |
| `static int` | `MAX_SLICES`             The maximum number of slices for cylinders cached by this class is 14, while the minimum will be the MIN\_SLICES declared in class Cylinder, which is 3. |


| **Constructor Summary** | |
| --- | --- |
| `CylinderReferences()`             Constructs a CylinderReferences object. |


| **Method Summary** | |
| --- | --- |
| `void` | `cacheCylinderDisplayList(GL gl, Cylinder cylinder, CylinderListInfo info)`             Caches a new OpenGL display list for a BALLS\_AND\_STICKS cylinder or a STICKS cylinder with the requested number of slices and stacks. |
| `void` | `cacheDefaultCylinders(GL gl, Cylinder cylinder)`             Caches a collection of cylinders using OpenGL display lists. |
| `CylinderListInfo` | `getBallsAndSticksCylinderInfo()`             Returns the CylinderListInfo object that holds the information on an OpenGL display list for a cylinder to be used for a BALLS\_AND\_STICKS style display. |
| `int` | `getBallsAndSticksRef()`             Returns the name (an integer) of an OpenGL display list for a cylinder intended to be used for Balls-and-Sticks style displays. |
| `int` | `getBallsAndSticksRef(double cameraDistance)`             Returns the name (an integer) of an OpenGL display list for a cylinder intended to be used for Balls-and-Sticks displays. |
| `CylinderListInfo` | `getSticksCylinderInfo()`             Returns the CylinderListInfo object that holds the information on an OpenGL display list for a cylinder to be used for a STICKS style display. |
| `int` | `getSticksRef()`             Returns the name (an integer) of an OpenGL display list for a cylinder intended to be used for Sticks style displays. |
| `int` | `getSticksRef(double cameraDistance)`             Returns the name (an integer) of an OpenGL display list for a cylinder intended to be used for Sticks style displays. |
| `void` | `printAutoTilingNumbers(boolean b)`             If automatic tiling is in use, giving this method an argument of true will cause the tiling numbers to be printed to standard out for testing and debugging purposes. |

| **Methods inherited from class java.lang.Object** |
| --- |
| `clone, equals, finalize, getClass, hashCode, notify, notifyAll, toString, wait, wait, wait` |

| **Field Detail** |
| --- |

### MAX\_SLICES

```
public static final int MAX_SLICES
```

:   The maximum number of slices for cylinders cached by this
    class is 14, while the minimum will be the MIN\_SLICES
    declared in class Cylinder, which is 3.

    **See Also:**: Constant Field Values

---


### DEFAULT\_HEIGHT

```
public static final double DEFAULT_HEIGHT
```

:   The default cylinder height is 1.0.

    **See Also:**: Constant Field Values

---


### DEFAULT\_RADIUS

```
public static final double DEFAULT_RADIUS
```

:   Cylinders intended for a Sticks type of display will use
    a default cylinder radius is 0.15. Cylinders intended for
    a Balls-and-Sticks type display will use one-half of the
    default radius.

    **See Also:**: Constant Field Values

---


### DEFAULT\_SLICES

```
public static final int DEFAULT_SLICES
```

:   The default number of slices for a cylinder is 10.

    **See Also:**: Constant Field Values

---


### DEFAULT\_STACKS

```
public static final int DEFAULT_STACKS
```

:   The default number of stacks for a cylinder is 1.

    **See Also:**: Constant Field Values

---


### DEFAULT\_CAP\_TILING

```
public static final int DEFAULT_CAP_TILING
```

:   The default tiling (number of slices and stacks) for
    a capping sphere is 5.

    **See Also:**: Constant Field Values

---


### COEFFICIENT

```
public static final double COEFFICIENT
```

:   The empirically-determined coefficient for the power
    equation is 29.059.

    **See Also:**: Constant Field Values

---


### EXPONENT

```
public static final double EXPONENT
```

:   The empirically-determined exponent for the power
    equation is -0.5267.

    **See Also:**: Constant Field Values


| **Constructor Detail** |
| --- |

### CylinderReferences

```
public CylinderReferences()
```

:   Constructs a CylinderReferences object.


| **Method Detail** |
| --- |

### printAutoTilingNumbers

```
public void printAutoTilingNumbers(boolean b)
```

:   If automatic tiling is in use, giving this method an argument of
    true will cause the tiling numbers to be printed to standard out
    for testing and debugging purposes.

    :   **Parameters:**: `b` - boolean value for printing tiling numbers.

---


### cacheDefaultCylinders

```
public void cacheDefaultCylinders(GL gl,
                                  Cylinder cylinder)
```

:   Caches a collection of cylinders using OpenGL display lists.

---


### cacheCylinderDisplayList

```
public void cacheCylinderDisplayList(GL gl,
                                     Cylinder cylinder,
                                     CylinderListInfo info)
```

:   Caches a new OpenGL display list for a BALLS\_AND\_STICKS cylinder or
    a STICKS cylinder with the requested number of slices and stacks.

---


### getBallsAndSticksCylinderInfo

```
public CylinderListInfo getBallsAndSticksCylinderInfo()
```

:   Returns the CylinderListInfo object that holds the information
    on an OpenGL display list for a cylinder to be used for a
    BALLS\_AND\_STICKS style display.

---


### getSticksCylinderInfo

```
public CylinderListInfo getSticksCylinderInfo()
```

:   Returns the CylinderListInfo object that holds the information on
    an OpenGL display list for a cylinder to be used for a STICKS
    style display.

---


### getBallsAndSticksRef

```
public int getBallsAndSticksRef()
```

:   Returns the name (an integer) of an OpenGL display list for a
    cylinder intended to be used for Balls-and-Sticks style displays. The
    cylinder will have DEFAULT\_SLICES number of slices, unless the
    cacheCylinderDisplayList() method has been used to change the
    number of slices. If this method is called before a display list
    has been cached, zero will be returned.

    :   **Returns:**: An integer reference to an OpenGL display list for a cylinder.

---


### getBallsAndSticksRef

```
public int getBallsAndSticksRef(double cameraDistance)
```

:   Returns the name (an integer) of an OpenGL display list for a
    cylinder intended to be used for Balls-and-Sticks displays. The
    number of slices for the cylinder will depend on the camera
    distance.

    :   **Parameters:**: `cameraDistance` - the distance between the center of the cylinder and the camera. **Returns:**: An integer reference to an OpenGL display list for a cylinder.

---


### getSticksRef

```
public int getSticksRef()
```

:   Returns the name (an integer) of an OpenGL display list for a
    cylinder intended to be used for Sticks style displays. The cylinder
    will have DEFAULT\_SLICES number of slices, unless the
    cacheCylinderDisplayList() method has been used to change the
    number of slices. If this method is called before a display
    list has been cached, zero will be returned.

    :   **Returns:**: An integer reference to an OpenGL display list for a cylinder.

---


### getSticksRef

```
public int getSticksRef(double cameraDistance)
```

:   Returns the name (an integer) of an OpenGL display list for a
    cylinder intended to be used for Sticks style displays. The number
    of slices for the cylinder will depend on the camera distance.

    :   **Parameters:**: `cameraDistance` - the distance between the center of the cylinder and the camera. **Returns:**: An integer reference to an OpenGL display list for a cylinder.


---


|  |  |  |  |  |  |  |  |  |  |  |
| --- | --- | --- | --- | --- | --- | --- | --- | --- | --- | --- |
| |  |  |  |  |  |  |  |  | | --- | --- | --- | --- | --- | --- | --- | --- | | **Overview** | **Package** | **Class** | **Use** | **Tree** | **Deprecated** | **Index** | **Help** | | |  |
| **PREV CLASS**   **NEXT CLASS** | **FRAMES**    **NO FRAMES**     **All Classes** |
| SUMMARY: NESTED | FIELD | CONSTR | METHOD | DETAIL: FIELD | CONSTR | METHOD |


---

# *Copyright © 2007-2008*
