# Supplementary material for: ProteinShader: illustrative rendering of macromolecules
Source: BMC Struct Biol. 2009 Mar 30;9:19. doi: 10.1186/1472-6807-9-19 (PMC2672931; doi:10.1186/1472-6807-9-19)
Supplement: Additional file 1 — ProteinShader program without source code. This compressed file contains the complete ProteinShader program including associated libraries, but no source code. A README.txt file gives an overview of the ProteinShader distribution, and the index.html file in the help subdirectory has directions on getting started with the program as well as a set of tutorials. [file 1472-6807-9-19-S1.zip › ProteinShader-beta-0_9_4-binary/help/api/org/proteinshader/graphics/displaylists/SphereListInfo.html]

SphereListInfo (ProteinShader API)


|  |  |  |  |  |  |  |  |  |  |  |
| --- | --- | --- | --- | --- | --- | --- | --- | --- | --- | --- |
| |  |  |  |  |  |  |  |  | | --- | --- | --- | --- | --- | --- | --- | --- | | **Overview** | **Package** | **Class** | **Use** | **Tree** | **Deprecated** | **Index** | **Help** | | |  |
| **PREV CLASS**   **NEXT CLASS** | **FRAMES**    **NO FRAMES**     **All Classes** |
| SUMMARY: NESTED | FIELD | CONSTR | METHOD | DETAIL: FIELD | CONSTR | METHOD |


---


## org.proteinshader.graphics.displaylists Class SphereListInfo

```
java.lang.Object
  org.proteinshader.graphics.displaylists.GeometricListInfo
      org.proteinshader.graphics.displaylists.SphereListInfo
```

---

``` public class SphereListInfo extends GeometricListInfo ```

Stores information on an OpenGL display list for a sphere. Methods
for actually creating the OpenGL display list are in the Sphere class.
This class is only for storing information on how the Sphere was drawn
(slices, stacks, and radius).

---

| **Constructor Summary** | |
| --- | --- |
| `SphereListInfo()`             Constructs a SphereListInfo. |
| `SphereListInfo(int displayListName, StyleEnum style, double radius, int slices, int stacks)`             Constructs a SphereListInfo. |


| **Method Summary** | |
| --- | --- |
| `double` | `getRadius()`             Returns the radius used to draw the sphere in the display list. |
| `int` | `getSlices()`             Returns the number of slices that were used to draw the sphere stored in the display list. |
| `int` | `getStacks()`             Returns the number of stacks that were used to draw the sphere stored in the display list. |
| `void` | `setRadius(double radius)`             Sets the radius used to draw the sphere in the display list. |
| `void` | `setSlices(int slices)`             Sets the number of slices that were used to draw the sphere stored in the display list. |
| `void` | `setStacks(int stacks)`             Sets the number of stacks that were used to draw the sphere in the display list. |

| **Methods inherited from class org.proteinshader.graphics.displaylists.GeometricListInfo** |
| --- |
| `getDisplayListName, getStyle, setDisplayListName, setStyle` |

| **Methods inherited from class java.lang.Object** |
| --- |
| `clone, equals, finalize, getClass, hashCode, notify, notifyAll, toString, wait, wait, wait` |

| **Constructor Detail** |
| --- |

### SphereListInfo

```
public SphereListInfo()
```

:   Constructs a SphereListInfo. Attributes are set to zero or null.

---


### SphereListInfo

```
public SphereListInfo(int displayListName,
                      StyleEnum style,
                      double radius,
                      int slices,
                      int stacks)
```

:   Constructs a SphereListInfo.

    **Parameters:**: `displayListName` - the name (an integer) of an OpenGL display list that stores commands to draw a sphere.: `style` - the style as a StyleEnum.: `radius` - the radius of the sphere.: `slices` - the number of slices in the sphere.: `stacks` - the number of stacks in the sphere.


| **Method Detail** |
| --- |

### getRadius

```
public double getRadius()
```

:   Returns the radius used to draw the sphere in the display list.
    The radius will usually be 1.0 because that makes it easy to scale
    the sphere to any required size by calling glScaled( newRadius,
    newRadius, newRadius) before plugging the display list name into
    glCallList().

    :   **Returns:**: The radius of the sphere saved in the display list.

---


### setRadius

```
public void setRadius(double radius)
```

:   Sets the radius used to draw the sphere in the display list. The
    radius will usually be 1.0 because that makes it easy to scale the
    sphere to any required size by calling glScaled( newRadius,
    newRadius, newRadius) before plugging the display list name into
    glCallList().

    :   **Parameters:**: `radius` - the radius of the sphere saved in the display list.

---


### getSlices

```
public int getSlices()
```

:   Returns the number of slices that were used to draw the sphere
    stored in the display list.

    :   **Returns:**: The number of slices in the sphere.

---


### setSlices

```
public void setSlices(int slices)
```

:   Sets the number of slices that were used to draw the sphere stored
    in the display list.

    :   **Parameters:**: `slices` - the number of slices in the sphere.

---


### getStacks

```
public int getStacks()
```

:   Returns the number of stacks that were used to draw the sphere
    stored in the display list.

    :   **Returns:**: The number of stacks in the sphere.

---


### setStacks

```
public void setStacks(int stacks)
```

:   Sets the number of stacks that were used to draw the sphere in the
    display list.

    :   **Parameters:**: `stacks` - the number of stacks in the sphere.


---


|  |  |  |  |  |  |  |  |  |  |  |
| --- | --- | --- | --- | --- | --- | --- | --- | --- | --- | --- |
| |  |  |  |  |  |  |  |  | | --- | --- | --- | --- | --- | --- | --- | --- | | **Overview** | **Package** | **Class** | **Use** | **Tree** | **Deprecated** | **Index** | **Help** | | |  |
| **PREV CLASS**   **NEXT CLASS** | **FRAMES**    **NO FRAMES**     **All Classes** |
| SUMMARY: NESTED | FIELD | CONSTR | METHOD | DETAIL: FIELD | CONSTR | METHOD |


---

# *Copyright © 2007-2008*
