# Supplementary material for: ProteinShader: illustrative rendering of macromolecules
Source: BMC Struct Biol. 2009 Mar 30;9:19. doi: 10.1186/1472-6807-9-19 (PMC2672931; doi:10.1186/1472-6807-9-19)
Supplement: Additional file 1 — ProteinShader program without source code. This compressed file contains the complete ProteinShader program including associated libraries, but no source code. A README.txt file gives an overview of the ProteinShader distribution, and the index.html file in the help subdirectory has directions on getting started with the program as well as a set of tutorials. [file 1472-6807-9-19-S1.zip › ProteinShader-beta-0_9_4-binary/help/api/org/proteinshader/graphics/displaylists/package-frame.html]

org.proteinshader.graphics.displaylists (ProteinShader API)


org.proteinshader.graphics.displaylists

|  |
| --- |
| Classes    CylinderListInfo   CylinderReferences   GeometricListInfo   SegmentListInfo   SegmentReferences   SphereListInfo   SphereReferences |
