# Supplementary material for: ProteinShader: illustrative rendering of macromolecules
Source: BMC Struct Biol. 2009 Mar 30;9:19. doi: 10.1186/1472-6807-9-19 (PMC2672931; doi:10.1186/1472-6807-9-19)
Supplement: Additional file 1 — ProteinShader program without source code. This compressed file contains the complete ProteinShader program including associated libraries, but no source code. A README.txt file gives an overview of the ProteinShader distribution, and the index.html file in the help subdirectory has directions on getting started with the program as well as a set of tutorials. [file 1472-6807-9-19-S1.zip › ProteinShader-beta-0_9_4-binary/help/api/org/proteinshader/graphics/displaylists/GeometricListInfo.html]

GeometricListInfo (ProteinShader API)


|  |  |  |  |  |  |  |  |  |  |  |
| --- | --- | --- | --- | --- | --- | --- | --- | --- | --- | --- |
| |  |  |  |  |  |  |  |  | | --- | --- | --- | --- | --- | --- | --- | --- | | **Overview** | **Package** | **Class** | **Use** | **Tree** | **Deprecated** | **Index** | **Help** | | |  |
| **PREV CLASS**   **NEXT CLASS** | **FRAMES**    **NO FRAMES**     **All Classes** |
| SUMMARY: NESTED | FIELD | CONSTR | METHOD | DETAIL: FIELD | CONSTR | METHOD |


---


## org.proteinshader.graphics.displaylists Class GeometricListInfo

```
java.lang.Object
  org.proteinshader.graphics.displaylists.GeometricListInfo
```

**Direct Known Subclasses:**: CylinderListInfo, SegmentListInfo, SphereListInfo

---

``` public abstract class GeometricListInfo extends Object ```

The concrete subclasses of this abstract class are used to store
information on an OpenGL display list that hold the commands to draw
a geometric object. A GeometricListInfo object is only for storing
information on an OpenGL display list. The code to actually create an
OpenGL display list in graphics card memory is in Shape subclasses
such as Sphere or Cylinder.
