# Supplementary material for: ProteinShader: illustrative rendering of macromolecules
Source: BMC Struct Biol. 2009 Mar 30;9:19. doi: 10.1186/1472-6807-9-19 (PMC2672931; doi:10.1186/1472-6807-9-19)
Supplement: Additional file 1 — ProteinShader program without source code. This compressed file contains the complete ProteinShader program including associated libraries, but no source code. A README.txt file gives an overview of the ProteinShader distribution, and the index.html file in the help subdirectory has directions on getting started with the program as well as a set of tutorials. [file 1472-6807-9-19-S1.zip › ProteinShader-beta-0_9_4-binary/help/api/org/proteinshader/graphics/displaylists/SphereReferences.html]

SphereReferences (ProteinShader API)


|  |  |  |  |  |  |  |  |  |  |  |
| --- | --- | --- | --- | --- | --- | --- | --- | --- | --- | --- |
| |  |  |  |  |  |  |  |  | | --- | --- | --- | --- | --- | --- | --- | --- | | **Overview** | **Package** | **Class** | **Use** | **Tree** | **Deprecated** | **Index** | **Help** | | |  |
| **PREV CLASS**   NEXT CLASS | **FRAMES**    **NO FRAMES**     **All Classes** |
| SUMMARY: NESTED | FIELD | CONSTR | METHOD | DETAIL: FIELD | CONSTR | METHOD |


---


## org.proteinshader.graphics.displaylists Class SphereReferences

```
java.lang.Object
  org.proteinshader.graphics.displaylists.SphereReferences
```

---

``` public class SphereReferences extends Object ```

Stores information on multiple OpenGL display lists that can be used
for rendering spheres with different degrees of detail.

---

| **Field Summary** | |
| --- | --- |
| `static double` | `COEFFICIENT`             The empirically-determined coefficient for the power equation is 226.1. |
| `static double` | `DEFAULT_RADIUS`             The spheres cached by this class will use a default radius of 1.0. |
| `static int` | `DEFAULT_TILING`             The default tiling is 12, and is used to set the initial number of slices and stacks for a generic sphere for SPACE\_FILLING style displays and a generic sphere for BALLS\_AND\_STICKS style displays. |
| `static double` | `EXPONENT`             The empirically-determined exponent for the power equation is -0.7367. |
| `static int` | `MAX_TILING`             The maximum tiling number (slices and stacks) for spheres cached by this class is 57, while the minimum will be the MIN\_TILING declared in class Sphere, which is 3. |


| **Constructor Summary** | |
| --- | --- |
| `SphereReferences()`             Constructs a SphereReferences object. |


| **Method Summary** | |
| --- | --- |
| `void` | `cacheDefaultSpheres(GL gl, Sphere sphere)`             Caches a collection of spheres using OpenGL display lists. |
| `void` | `cacheSphereDisplayList(GL gl, Sphere sphere, SphereListInfo info)`             Caches a new OpenGL display list for a SPACE\_FILLING sphere or a BALLS\_AND\_STICKS sphere with the requested tiling (number of slices and stacks). |
| `int` | `getBallsAndSticksRef()`             Returns the name (an integer) of an OpenGL display list for a sphere intended to be used for a Balls-and-Sticks style display. |
| `int` | `getBallsAndSticksRef(double cameraDistance)`             Returns the name (an integer) of an OpenGL display list for a sphere intended to be used for a Balls-and-Sticks style display. |
| `SphereListInfo` | `getBallsAndSticksSphereInfo()`             Returns the SphereListInfo object that holds the information on an OpenGL display list for a sphere to be used in a BALLS\_AND\_STICKS style display. |
| `int` | `getSpaceFillingRef()`             Returns the name (an integer) of an OpenGL display list for a sphere intended to be used for Space-Filling style displays. |
| `int` | `getSpaceFillingRef(double cameraDistance)`             Returns the name (an integer) of an OpenGL display list for a sphere intended to be used for a Space-Filling style display. |
| `int` | `getSpaceFillingSlices()`             Returns the number of slices that should be used for a sphere if automatic calculation of tiling number is turned off. |
| `SphereListInfo` | `getSpaceFillingSphereInfo()`             Returns the SphereListInfo object that holds the information on an OpenGL display list for a sphere to be used for SPACE\_FILLING style. |
| `int` | `getSpaceFillingStacks()`             Returns the number of stacks that should be used for a sphere if automatic calculation of tiling number is turned off. |
| `int` | `getSpaceFillingTilingNumber(double cameraDistance)`             Returns the recommended level of detail (tiling number = slices = stacks) for rendering a sphere at a given camera distance in angstroms. |
| `void` | `printAutoTilingNumbers(boolean b)`             If automatic tiling is in use, giving this method an argument of true will cause the tiling numbers to be printed to standard out for testing and debugging purposes. |

| **Methods inherited from class java.lang.Object** |
| --- |
| `clone, equals, finalize, getClass, hashCode, notify, notifyAll, toString, wait, wait, wait` |

| **Field Detail** |
| --- |

### DEFAULT\_RADIUS

```
public static final double DEFAULT_RADIUS
```

:   The spheres cached by this class will use a default radius
    of 1.0.

    **See Also:**: Constant Field Values

---


### MAX\_TILING

```
public static final int MAX_TILING
```

:   The maximum tiling number (slices and stacks) for spheres
    cached by this class is 57, while the minimum will be the
    MIN\_TILING declared in class Sphere, which is 3.

    **See Also:**: Constant Field Values

---


### DEFAULT\_TILING

```
public static final int DEFAULT_TILING
```

:   The default tiling is 12, and is used to set the initial
    number of slices and stacks for a generic sphere for
    SPACE\_FILLING style displays and a generic sphere for
    BALLS\_AND\_STICKS style displays.

    **See Also:**: Constant Field Values

---


### COEFFICIENT

```
public static final double COEFFICIENT
```

:   The empirically-determined coefficient for the power
    equation is 226.1.

    **See Also:**: Constant Field Values

---


### EXPONENT

```
public static final double EXPONENT
```

:   The empirically-determined exponent for the power
    equation is -0.7367.

    **See Also:**: Constant Field Values


| **Constructor Detail** |
| --- |

### SphereReferences

```
public SphereReferences()
```

:   Constructs a SphereReferences object.


| **Method Detail** |
| --- |

### printAutoTilingNumbers

```
public void printAutoTilingNumbers(boolean b)
```

:   If automatic tiling is in use, giving this method an argument of
    true will cause the tiling numbers to be printed to standard out
    for testing and debugging purposes.

    :   **Parameters:**: `b` - boolean value for printing tiling numbers.

---


### cacheDefaultSpheres

```
public void cacheDefaultSpheres(GL gl,
                                Sphere sphere)
```

:   Caches a collection of spheres using OpenGL display lists.

---


### cacheSphereDisplayList

```
public void cacheSphereDisplayList(GL gl,
                                   Sphere sphere,
                                   SphereListInfo info)
```

:   Caches a new OpenGL display list for a SPACE\_FILLING sphere or a
    BALLS\_AND\_STICKS sphere with the requested tiling (number of slices
    and stacks).

---


### getSpaceFillingSphereInfo

```
public SphereListInfo getSpaceFillingSphereInfo()
```

:   Returns the SphereListInfo object that holds the information on an
    OpenGL display list for a sphere to be used for SPACE\_FILLING style.

---


### getSpaceFillingSlices

```
public int getSpaceFillingSlices()
```

:   Returns the number of slices that should be used for a sphere if automatic
    calculation of tiling number is turned off.

    :   **Returns:**: The number of slices for a space filling sphere.

---


### getSpaceFillingStacks

```
public int getSpaceFillingStacks()
```

:   Returns the number of stacks that should be used for a sphere if automatic
    calculation of tiling number is turned off.

    :   **Returns:**: The number of stacks for a space filling sphere.

---


### getSpaceFillingTilingNumber

```
public int getSpaceFillingTilingNumber(double cameraDistance)
```

:   Returns the recommended level of detail (tiling number = slices = stacks)
    for rendering a sphere at a given camera distance in angstroms.

    :   **Parameters:**: `cameraDistance` - the distance between the center of the sphere and the camera. **Returns:**: The tiling number.

---


### getBallsAndSticksSphereInfo

```
public SphereListInfo getBallsAndSticksSphereInfo()
```

:   Returns the SphereListInfo object that holds the information on an
    OpenGL display list for a sphere to be used in a BALLS\_AND\_STICKS style
    display.

---


### getSpaceFillingRef

```
public int getSpaceFillingRef()
```

:   Returns the name (an integer) of an OpenGL display list for a
    sphere intended to be used for Space-Filling style displays. The sphere
    will have DEFAULT\_TILING number of slices and stacks, unless the
    cacheSphereDisplayList() method has been used to change the tiling
    number. If this method is called before a display list has been
    cached, zero will be returned.

    :   **Returns:**: An integer reference to an OpenGL display list for a sphere.

---


### getSpaceFillingRef

```
public int getSpaceFillingRef(double cameraDistance)
```

:   Returns the name (an integer) of an OpenGL display list for a
    sphere intended to be used for a Space-Filling style display. The tiling
    number (number of slices and stacks) of the sphere will depend on
    the camera distance.

    :   **Parameters:**: `cameraDistance` - the distance between the center of the sphere and the camera. **Returns:**: An integer reference to an OpenGL display list for a sphere.

---


### getBallsAndSticksRef

```
public int getBallsAndSticksRef()
```

:   Returns the name (an integer) of an OpenGL display list for a
    sphere intended to be used for a Balls-and-Sticks style display. The
    sphere will have DEFAULT\_TILING number of slices and stacks,
    unless the cacheSphereDisplayList() method has been used to change
    the tiling number. If this method is called before a display list
    has been cached, zero will be returned.

    :   **Returns:**: An integer reference to an OpenGL display list for a sphere.

---


### getBallsAndSticksRef

```
public int getBallsAndSticksRef(double cameraDistance)
```

:   Returns the name (an integer) of an OpenGL display list for a
    sphere intended to be used for a Balls-and-Sticks style display. The
    tiling number (number of slices and stacks) of the sphere will
    depend on the camera distance.

    :   **Parameters:**: `cameraDistance` - the distance between the center of the sphere and the camera. **Returns:**: An integer reference to an OpenGL display list for a sphere.


---


|  |  |  |  |  |  |  |  |  |  |  |
| --- | --- | --- | --- | --- | --- | --- | --- | --- | --- | --- |
| |  |  |  |  |  |  |  |  | | --- | --- | --- | --- | --- | --- | --- | --- | | **Overview** | **Package** | **Class** | **Use** | **Tree** | **Deprecated** | **Index** | **Help** | | |  |
| **PREV CLASS**   NEXT CLASS | **FRAMES**    **NO FRAMES**     **All Classes** |
| SUMMARY: NESTED | FIELD | CONSTR | METHOD | DETAIL: FIELD | CONSTR | METHOD |


---

# *Copyright © 2007-2008*
