# Supplementary material for: ProteinShader: illustrative rendering of macromolecules
Source: BMC Struct Biol. 2009 Mar 30;9:19. doi: 10.1186/1472-6807-9-19 (PMC2672931; doi:10.1186/1472-6807-9-19)
Supplement: Additional file 1 — ProteinShader program without source code. This compressed file contains the complete ProteinShader program including associated libraries, but no source code. A README.txt file gives an overview of the ProteinShader distribution, and the index.html file in the help subdirectory has directions on getting started with the program as well as a set of tutorials. [file 1472-6807-9-19-S1.zip › ProteinShader-beta-0_9_4-binary/help/api/org/proteinshader/graphics/displaylists/SegmentListInfo.html]

SegmentListInfo (ProteinShader API)


|  |  |  |  |  |  |  |  |  |  |  |
| --- | --- | --- | --- | --- | --- | --- | --- | --- | --- | --- |
| |  |  |  |  |  |  |  |  | | --- | --- | --- | --- | --- | --- | --- | --- | | **Overview** | **Package** | **Class** | **Use** | **Tree** | **Deprecated** | **Index** | **Help** | | |  |
| **PREV CLASS**   **NEXT CLASS** | **FRAMES**    **NO FRAMES**     **All Classes** |
| SUMMARY: NESTED | FIELD | CONSTR | METHOD | DETAIL: FIELD | CONSTR | METHOD |


---


## org.proteinshader.graphics.displaylists Class SegmentListInfo

```
java.lang.Object
  org.proteinshader.graphics.displaylists.GeometricListInfo
      org.proteinshader.graphics.displaylists.SegmentListInfo
```

---

``` public class SegmentListInfo extends GeometricListInfo ```

Stores information on an OpenGL display list for a Segment.

---

| **Constructor Summary** | |
| --- | --- |
| `SegmentListInfo()`             Constructs a SegmentListInfo. |
| `SegmentListInfo(int displayListName, StyleEnum style)`             Constructs a SegmentListInfo. |


| **Method Summary** | |
| --- | --- |
| `void` | `clear(GL gl)`             Frees graphics card memory for OpenGL display lists. |
| `int` | `getEndCapName()`             Returns a reference to the OpenGL display list for the end cap. |
| `int` | `getStartCapName()`             Returns a reference to the OpenGL display list for the start cap. |
| `int` | `getThinSidesName()`             Returns the reference to the OpenGL display list for the thin sides of a Ribbon. |
| `void` | `setEndCapName(int endCapName)`             Sets the reference to the OpenGL display list for the end cap. |
| `void` | `setStartCapName(int startCapName)`             Sets the reference to the OpenGL display list for the start cap. |
| `void` | `setThinSidesName(int thinSidesName)`             Sets the reference to the OpenGL display list for the thin sides of a Ribbon. |

| **Methods inherited from class org.proteinshader.graphics.displaylists.GeometricListInfo** |
| --- |
| `getDisplayListName, getStyle, setDisplayListName, setStyle` |

| **Methods inherited from class java.lang.Object** |
| --- |
| `clone, equals, finalize, getClass, hashCode, notify, notifyAll, toString, wait, wait, wait` |

| **Constructor Detail** |
| --- |

### SegmentListInfo

```
public SegmentListInfo()
```

:   Constructs a SegmentListInfo.

---


### SegmentListInfo

```
public SegmentListInfo(int displayListName,
                       StyleEnum style)
```

:   Constructs a SegmentListInfo.

    **Parameters:**: `displayListName` - the name (an integer) of an OpenGL display list that stores commands to draw a Segment.: `style` - the style as a StyleEnum.


| **Method Detail** |
| --- |

### clear

```
public void clear(GL gl)
```

:   Frees graphics card memory for OpenGL display lists.

    :   **Parameters:**: `gl` - the current GL object.

---


### getStartCapName

```
public int getStartCapName()
```

:   Returns a reference to the OpenGL display list for the start cap.

    :   **Returns:**: The name (an integer) of the start cap OpenGL display list.

---


### setStartCapName

```
public void setStartCapName(int startCapName)
```

:   Sets the reference to the OpenGL display list for the start cap.

    :   **Parameters:**: `startCapName` - the name (an integer) of the OpenGL display list for the start cap.

---


### getEndCapName

```
public int getEndCapName()
```

:   Returns a reference to the OpenGL display list for the end cap.

    :   **Returns:**: The name (an integer) of the end cap OpenGL display list.

---


### setEndCapName

```
public void setEndCapName(int endCapName)
```

:   Sets the reference to the OpenGL display list for the end cap.

    :   **Parameters:**: `endCapName` - the name (an integer) of the OpenGL display list for the end cap.

---


### getThinSidesName

```
public int getThinSidesName()
```

:   Returns the reference to the OpenGL display list for the thin
    sides of a Ribbon.

    :   **Returns:**: The name (an integer) of the OpenGL display for the thin sides of a Ribbon.

---


### setThinSidesName

```
public void setThinSidesName(int thinSidesName)
```

:   Sets the reference to the OpenGL display list for the thin sides
    of a Ribbon.

    :   **Parameters:**: `thinSidesName` - the name (an integer) of the OpenGL display list for the thin sides of a ribbon Segment.


---


|  |  |  |  |  |  |  |  |  |  |  |
| --- | --- | --- | --- | --- | --- | --- | --- | --- | --- | --- |
| |  |  |  |  |  |  |  |  | | --- | --- | --- | --- | --- | --- | --- | --- | | **Overview** | **Package** | **Class** | **Use** | **Tree** | **Deprecated** | **Index** | **Help** | | |  |
| **PREV CLASS**   **NEXT CLASS** | **FRAMES**    **NO FRAMES**     **All Classes** |
| SUMMARY: NESTED | FIELD | CONSTR | METHOD | DETAIL: FIELD | CONSTR | METHOD |


---

# *Copyright © 2007-2008*
