# Supplementary material for: ProteinShader: illustrative rendering of macromolecules
Source: BMC Struct Biol. 2009 Mar 30;9:19. doi: 10.1186/1472-6807-9-19 (PMC2672931; doi:10.1186/1472-6807-9-19)
Supplement: Additional file 1 — ProteinShader program without source code. This compressed file contains the complete ProteinShader program including associated libraries, but no source code. A README.txt file gives an overview of the ProteinShader distribution, and the index.html file in the help subdirectory has directions on getting started with the program as well as a set of tutorials. [file 1472-6807-9-19-S1.zip › ProteinShader-beta-0_9_4-binary/help/api/org/proteinshader/graphics/displaylists/CylinderListInfo.html]

CylinderListInfo (ProteinShader API)


|  |  |  |  |  |  |  |  |  |  |  |
| --- | --- | --- | --- | --- | --- | --- | --- | --- | --- | --- |
| |  |  |  |  |  |  |  |  | | --- | --- | --- | --- | --- | --- | --- | --- | | **Overview** | **Package** | **Class** | **Use** | **Tree** | **Deprecated** | **Index** | **Help** | | |  |
| PREV CLASS   **NEXT CLASS** | **FRAMES**    **NO FRAMES**     **All Classes** |
| SUMMARY: NESTED | FIELD | CONSTR | METHOD | DETAIL: FIELD | CONSTR | METHOD |


---


## org.proteinshader.graphics.displaylists Class CylinderListInfo

```
java.lang.Object
  org.proteinshader.graphics.displaylists.GeometricListInfo
      org.proteinshader.graphics.displaylists.CylinderListInfo
```

---

``` public class CylinderListInfo extends GeometricListInfo ```

Stores information on an OpenGL display list for a cylinder. Methods
for actually creating the display list are in the Cylinder class. This
class is only for storing a memory of how the cylinder was drawn
(baseRadius, topRadius, height, slices, stacks, *etc*.).

---

| **Constructor Summary** | |
| --- | --- |
| `CylinderListInfo()`             Constructs a CylinderListInfo. |
| `CylinderListInfo(int displayListName, StyleEnum style, double baseRadius, double topRadius, double height, int slices, int stacks, boolean baseCapped, boolean topCapped, int capSlices, int capStacks)`             Constructs a CylinderListInfo. |


| **Method Summary** | |
| --- | --- |
| `double` | `getBaseRadius()`             Returns the base radius of the cylinder in the display list. |
| `int` | `getCapSlices()`             Returns the number of slices that were used to draw the capping sphere at one or both ends of the cylinder (if a capping sphere was used). |
| `int` | `getCapStacks()`             Returns the number of stacks that were used to draw the capping sphere at one or both ends of the cylinder (if a capping sphere was used). |
| `double` | `getHeight()`             Returns the height of the cylinder stored in the display list. |
| `int` | `getSlices()`             Returns the number of slices that were used to draw the cylinder stored in the display list. |
| `int` | `getStacks()`             Returns the number of stacks that were used to draw the cylinder stored in the display list. |
| `double` | `getTopRadius()`             Returns the top radius of the cylinder in the display list. |
| `boolean` | `isBaseCapped()`             Returns true if a capping sphere was used at the base of the cylinder. |
| `boolean` | `isTopCapped()`             Returns true if a capping sphere was used at the top of the cylinder. |
| `void` | `setBaseCapped(boolean baseCapped)`             Sets a boolean indicating whether the base of the cylinder is capped with a small sphere. |
| `void` | `setBaseRadius(double baseRadius)`             Sets the base radius that was used to draw the cylinder in the display list. |
| `void` | `setCapSlices(int capSlices)`             Sets the number of slices that were used to draw the capping sphere at one or both ends of the cylinder (if a capping sphere was used). |
| `void` | `setCapStacks(int capStacks)`             Sets the number of stacks that were used to draw the capping sphere at one or both ends of the cylinder (if a capping sphere was used). |
| `void` | `setHeight(double height)`             Sets the height that was used to draw the cylinder stored in the display list. |
| `void` | `setSlices(int slices)`             Sets the number of slices that were used to draw the cylinder stored in thedisplay list. |
| `void` | `setStacks(int stacks)`             Sets the number of stacks that were used to draw the cylinder in the display list. |
| `void` | `setTopCapped(boolean topCapped)`             Sets a boolean indicating whether the top of the cylinder is capped with a small sphere. |
| `void` | `setTopRadius(double topRadius)`             Sets the top radius that was used to draw the cylinder in the display list. |

| **Methods inherited from class org.proteinshader.graphics.displaylists.GeometricListInfo** |
| --- |
| `getDisplayListName, getStyle, setDisplayListName, setStyle` |

| **Methods inherited from class java.lang.Object** |
| --- |
| `clone, equals, finalize, getClass, hashCode, notify, notifyAll, toString, wait, wait, wait` |

| **Constructor Detail** |
| --- |

### CylinderListInfo

```
public CylinderListInfo()
```

:   Constructs a CylinderListInfo. The attributes are set to zero,
    null, or false.

---


### CylinderListInfo

```
public CylinderListInfo(int displayListName,
                        StyleEnum style,
                        double baseRadius,
                        double topRadius,
                        double height,
                        int slices,
                        int stacks,
                        boolean baseCapped,
                        boolean topCapped,
                        int capSlices,
                        int capStacks)
```

:   Constructs a CylinderListInfo.

    **Parameters:**: `displayListName` - the name (an int) of an OpenGL display list that stores commands to draw a cylinder.: `style` - the style as a StyleEnum.: `baseRadius` - the radius at the base of the cylinder.: `topRadius` - the radius at the top of the cylinder.: `height` - the height of the cylinder.: `slices` - the number of slices in the cylinder.: `stacks` - the number of stacks in the cylinder.: `baseCapped` - true if the base is capped with a sphere.: `topCapped` - true if the top is capped with a sphere.: `capSlices` - the number of slices in the capping sphere.: `capStacks` - the number of stacks in the capping sphere.


| **Method Detail** |
| --- |

### getBaseRadius

```
public double getBaseRadius()
```

:   Returns the base radius of the cylinder in the display list.

    :   **Returns:**: The base radius of the cylinder.

---


### setBaseRadius

```
public void setBaseRadius(double baseRadius)
```

:   Sets the base radius that was used to draw the cylinder in the display list.

    :   **Parameters:**: `baseRadius` - the base radius of the cylinder.

---


### getTopRadius

```
public double getTopRadius()
```

:   Returns the top radius of the cylinder in the display list.

    :   **Returns:**: The top radius of the cylinder.

---


### setTopRadius

```
public void setTopRadius(double topRadius)
```

:   Sets the top radius that was used to draw the cylinder in the display list.

    :   **Parameters:**: `topRadius` - the top radius of the cylinder.

---


### getHeight

```
public double getHeight()
```

:   Returns the height of the cylinder stored in the display list.

    :   **Returns:**: The height of the cylinder.

---


### setHeight

```
public void setHeight(double height)
```

:   Sets the height that was used to draw the cylinder stored in the display
    list.

    :   **Parameters:**: `height` - the height of the cylinder.

---


### getSlices

```
public int getSlices()
```

:   Returns the number of slices that were used to draw the cylinder
    stored in the display list.

    :   **Returns:**: The number of slices in the cylinder.

---


### setSlices

```
public void setSlices(int slices)
```

:   Sets the number of slices that were used to draw the cylinder
    stored in thedisplay list.

    :   **Parameters:**: `slices` - the number of slices in the cylinder.

---


### getStacks

```
public int getStacks()
```

:   Returns the number of stacks that were used to draw the cylinder
    stored in the display list.

    :   **Returns:**: The number of stacks in the cylinder.

---


### setStacks

```
public void setStacks(int stacks)
```

:   Sets the number of stacks that were used to draw the cylinder in
    the display list.

    :   **Parameters:**: `stacks` - the number of stacks in the cylinder.

---


### isBaseCapped

```
public boolean isBaseCapped()
```

:   Returns true if a capping sphere was used at the base of the
    cylinder. Otherwise, returns false.

    :   **Returns:**: A boolean indicating if the cylinder base has a cap.

---


### setBaseCapped

```
public void setBaseCapped(boolean baseCapped)
```

:   Sets a boolean indicating whether the base of the cylinder is
    capped with a small sphere.

    :   **Parameters:**: `baseCapped` - a boolean indicating if the base is capped.

---


### isTopCapped

```
public boolean isTopCapped()
```

:   Returns true if a capping sphere was used at the top of the
    cylinder. Otherwise, returns false.

    :   **Returns:**: A boolean indicating if the cylinder top has a cap.

---


### setTopCapped

```
public void setTopCapped(boolean topCapped)
```

:   Sets a boolean indicating whether the top of the cylinder is
    capped with a small sphere.

    :   **Parameters:**: `topCapped` - a boolean indicating if the top is capped.

---


### getCapSlices

```
public int getCapSlices()
```

:   Returns the number of slices that were used to draw the capping
    sphere at one or both ends of the cylinder (if a capping sphere
    was used).

    :   **Returns:**: The number of slices in the capping sphere.

---


### setCapSlices

```
public void setCapSlices(int capSlices)
```

:   Sets the number of slices that were used to draw the capping
    sphere at one or both ends of the cylinder (if a capping sphere
    was used).

    :   **Parameters:**: `capSlices` - the number of slices in the capping sphere.

---


### getCapStacks

```
public int getCapStacks()
```

:   Returns the number of stacks that were used to draw the capping
    sphere at one or both ends of the cylinder (if a capping sphere
    was used).

    :   **Returns:**: The number of stacks in the capping sphere.

---


### setCapStacks

```
public void setCapStacks(int capStacks)
```

:   Sets the number of stacks that were used to draw the capping
    sphere at one or both ends of the cylinder (if a capping sphere
    was used).

    :   **Parameters:**: `capStacks` - the number of stacks in the capping sphere.


---


|  |  |  |  |  |  |  |  |  |  |  |
| --- | --- | --- | --- | --- | --- | --- | --- | --- | --- | --- |
| |  |  |  |  |  |  |  |  | | --- | --- | --- | --- | --- | --- | --- | --- | | **Overview** | **Package** | **Class** | **Use** | **Tree** | **Deprecated** | **Index** | **Help** | | |  |
| PREV CLASS   **NEXT CLASS** | **FRAMES**    **NO FRAMES**     **All Classes** |
| SUMMARY: NESTED | FIELD | CONSTR | METHOD | DETAIL: FIELD | CONSTR | METHOD |


---

# *Copyright © 2007-2008*
