# Supplementary material for: ProteinShader: illustrative rendering of macromolecules
Source: BMC Struct Biol. 2009 Mar 30;9:19. doi: 10.1186/1472-6807-9-19 (PMC2672931; doi:10.1186/1472-6807-9-19)
Supplement: Additional file 1 — ProteinShader program without source code. This compressed file contains the complete ProteinShader program including associated libraries, but no source code. A README.txt file gives an overview of the ProteinShader distribution, and the index.html file in the help subdirectory has directions on getting started with the program as well as a set of tutorials. [file 1472-6807-9-19-S1.zip › ProteinShader-beta-0_9_4-binary/help/api/org/proteinshader/graphics/displaylists/package-summary.html]

org.proteinshader.graphics.displaylists (ProteinShader API)


|  |  |  |  |  |  |  |  |  |  |  |
| --- | --- | --- | --- | --- | --- | --- | --- | --- | --- | --- |
| |  |  |  |  |  |  |  |  | | --- | --- | --- | --- | --- | --- | --- | --- | | **Overview** | **Package** | Class | **Use** | **Tree** | **Deprecated** | **Index** | **Help** | | |  |
| **PREV PACKAGE**   **NEXT PACKAGE** | **FRAMES**    **NO FRAMES**     **All Classes** |


---

## Package org.proteinshader.graphics.displaylists

Holds the classes needed to manage OpenGL display lists, which are used
to cache reusable geometry for spheres, cylinders, ribbon segments, and
tube segments.

**See:**
  
          **Description**

| **Class Summary** | |
| --- | --- |
| **CylinderListInfo** | Stores information on an OpenGL display list for a cylinder. |
| **CylinderReferences** | Stores information on multiple OpenGL display lists that can be used for rendering cylinders with different degrees of detail. |
| **GeometricListInfo** | The concrete subclasses of this abstract class are used to store information on an OpenGL display list that hold the commands to draw a geometric object. |
| **SegmentListInfo** | Stores information on an OpenGL display list for a Segment. |
| **SegmentReferences** | Stores information on multiple OpenGL display lists that can be used for rendering Segments with different degrees of detail (this first version only holds one OpenGL display list for each Segment, but a future version will likely hold multiple OpenGL display lists for each Segment (with varying tiling number) so that the level of detail to be used can be calculated based on camera distance). |
| **SphereListInfo** | Stores information on an OpenGL display list for a sphere. |
| **SphereReferences** | Stores information on multiple OpenGL display lists that can be used for rendering spheres with different degrees of detail. |

## Package org.proteinshader.graphics.displaylists Description

Holds the classes needed to manage OpenGL display lists, which are used
to cache reusable geometry for spheres, cylinders, ribbon segments, and
tube segments.
