# Supplementary material for: ProteinShader: illustrative rendering of macromolecules
Source: BMC Struct Biol. 2009 Mar 30;9:19. doi: 10.1186/1472-6807-9-19 (PMC2672931; doi:10.1186/1472-6807-9-19)
Supplement: Additional file 1 — ProteinShader program without source code. This compressed file contains the complete ProteinShader program including associated libraries, but no source code. A README.txt file gives an overview of the ProteinShader distribution, and the index.html file in the help subdirectory has directions on getting started with the program as well as a set of tutorials. [file 1472-6807-9-19-S1.zip › ProteinShader-beta-0_9_4-binary/help/api/org/proteinshader/graphics/displaylists/package-use.html]

Uses of Package org.proteinshader.graphics.displaylists (ProteinShader API)


|  |  |  |  |  |  |  |  |  |  |  |
| --- | --- | --- | --- | --- | --- | --- | --- | --- | --- | --- |
| |  |  |  |  |  |  |  |  | | --- | --- | --- | --- | --- | --- | --- | --- | | **Overview** | **Package** | Class | **Use** | **Tree** | **Deprecated** | **Index** | **Help** | | |  |
| PREV   NEXT | **FRAMES**    **NO FRAMES**     **All Classes** |


---


## **Uses of Package org.proteinshader.graphics.displaylists**

| Packages that use org.proteinshader.graphics.displaylists | |
| --- | --- |
| **org.proteinshader.graphics** | Holds the drawing classes: Ribbon, Tube, FrenetFrames, Sphere, and Cylinder. |
| **org.proteinshader.graphics.adapter** | Holds the StructureToGraphics class, which is used to manage the use of the drawing classes and OpenGL display lists. |
| **org.proteinshader.graphics.displaylists** | Holds the classes needed to manage OpenGL display lists, which are used to cache reusable geometry for spheres, cylinders, ribbon segments, and tube segments. |
| **org.proteinshader.gui** | Holds all of the Swing GUI components and their associated listeners, including class Renderer, which is registered as a listener for the GLCanvas object that is used a drawing surface. |

| Classes in org.proteinshader.graphics.displaylists used by org.proteinshader.graphics | |
| --- | --- |
| ****CylinderListInfo****             Stores information on an OpenGL display list for a cylinder. |
| ****SegmentListInfo****             Stores information on an OpenGL display list for a Segment. |
| ****SphereListInfo****             Stores information on an OpenGL display list for a sphere. |

| Classes in org.proteinshader.graphics.displaylists used by org.proteinshader.graphics.adapter | |
| --- | --- |
| ****CylinderListInfo****             Stores information on an OpenGL display list for a cylinder. |
| ****GeometricListInfo****             The concrete subclasses of this abstract class are used to store information on an OpenGL display list that hold the commands to draw a geometric object. |
| ****SphereListInfo****             Stores information on an OpenGL display list for a sphere. |

| Classes in org.proteinshader.graphics.displaylists used by org.proteinshader.graphics.displaylists | |
| --- | --- |
| ****CylinderListInfo****             Stores information on an OpenGL display list for a cylinder. |
| ****GeometricListInfo****             The concrete subclasses of this abstract class are used to store information on an OpenGL display list that hold the commands to draw a geometric object. |
| ****SegmentListInfo****             Stores information on an OpenGL display list for a Segment. |
| ****SphereListInfo****             Stores information on an OpenGL display list for a sphere. |

| Classes in org.proteinshader.graphics.displaylists used by org.proteinshader.gui | |
| --- | --- |
| ****CylinderListInfo****             Stores information on an OpenGL display list for a cylinder. |
| ****GeometricListInfo****             The concrete subclasses of this abstract class are used to store information on an OpenGL display list that hold the commands to draw a geometric object. |
| ****SphereListInfo****             Stores information on an OpenGL display list for a sphere. |

---


|  |  |  |  |  |  |  |  |  |  |  |
| --- | --- | --- | --- | --- | --- | --- | --- | --- | --- | --- |
| |  |  |  |  |  |  |  |  | | --- | --- | --- | --- | --- | --- | --- | --- | | **Overview** | **Package** | Class | **Use** | **Tree** | **Deprecated** | **Index** | **Help** | | |  |
| PREV   NEXT | **FRAMES**    **NO FRAMES**     **All Classes** |


---

# *Copyright © 2007-2008*
