# Supplementary material for: ProteinShader: illustrative rendering of macromolecules
Source: BMC Struct Biol. 2009 Mar 30;9:19. doi: 10.1186/1472-6807-9-19 (PMC2672931; doi:10.1186/1472-6807-9-19)
Supplement: Additional file 1 — ProteinShader program without source code. This compressed file contains the complete ProteinShader program including associated libraries, but no source code. A README.txt file gives an overview of the ProteinShader distribution, and the index.html file in the help subdirectory has directions on getting started with the program as well as a set of tutorials. [file 1472-6807-9-19-S1.zip › ProteinShader-beta-0_9_4-binary/help/api/org/proteinshader/graphics/textures/class-use/Texture.html]

Uses of Class org.proteinshader.graphics.textures.Texture (ProteinShader API)


|  |  |  |  |  |  |  |  |  |  |  |
| --- | --- | --- | --- | --- | --- | --- | --- | --- | --- | --- |
| |  |  |  |  |  |  |  |  | | --- | --- | --- | --- | --- | --- | --- | --- | | **Overview** | **Package** | **Class** | **Use** | **Tree** | **Deprecated** | **Index** | **Help** | | |  |
| PREV   NEXT | **FRAMES**    **NO FRAMES**     **All Classes** |


---


## **Uses of Class org.proteinshader.graphics.textures.Texture**

| Packages that use Texture | |
| --- | --- |
| **org.proteinshader.graphics.adapter** | Holds the StructureToGraphics class, which is used to manage the use of the drawing classes and OpenGL display lists. |
| **org.proteinshader.graphics.textures** | Holds the classes needed for creating and managing OpenGL texture objects. |
| **org.proteinshader.gui** | Holds all of the Swing GUI components and their associated listeners, including class Renderer, which is registered as a listener for the GLCanvas object that is used a drawing surface. |

| Uses of Texture in org.proteinshader.graphics.adapter | |
| --- | --- |

| Methods in org.proteinshader.graphics.adapter that return types with arguments of type Texture | |
| --- | --- |
| `Vector<Texture>` | `StructureToGraphics.getBendTextures()`             Returns the bend Texture objects that were created with the loadTextures() method and are intended for use with halftoning. |
| `Vector<Texture>` | `StructureToGraphics.getHalftoningTextures()`             Returns the Texture objects that were created with the loadTextures() method and are intended for use with halftoning. |
| `Vector<Texture>` | `StructureToGraphics.getPatternsTextures()`             Returns the Texture objects that were created with the loadTextures() method and are intended for use as patterns to apply to tubes and ribbons drawn in color (rather than halftoning). |

| Uses of Texture in org.proteinshader.graphics.textures | |
| --- | --- |

| Methods in org.proteinshader.graphics.textures that return types with arguments of type Texture | |
| --- | --- |
| `Vector<Texture>` | `TextureFactory.createBendTextures(GL gl)`             Creates Texture objects based on files named in BEND\_CONFIG. |
| `Vector<Texture>` | `TextureFactory.createHalftoningTextures(GL gl)`             Creates Texture objects based on files named in HALFTONING\_CONFIG. |
| `Vector<Texture>` | `TextureFactory.createPatternsTextures(GL gl)`             Creates Texture objects based on files named in PATTERNS\_CONFIG. |
| `Vector<Texture>` | `TextureFactory.createTextures(GL gl, String directory, String configName)`             Creates Texture objects based on files named in the CONFIG\_FILE. |
| `Vector<Texture>` | `TextureManager.getBendTextures()`             Returns a list of Java Texture objects with information on OpenGL texture objects that are currently stored on the graphics card. |
| `Vector<Texture>` | `TextureManager.getHalftoningTextures()`             Returns a list of Java Texture objects with information on OpenGL texture objects that are currently stored on the graphics card. |
| `Vector<Texture>` | `TextureManager.getPatternsTextures()`             Returns a list of Java Texture objects with information on OpenGL texture objects that are currently stored on the graphics card. |

| Methods in org.proteinshader.graphics.textures with parameters of type Texture | |
| --- | --- |
| `int` | `Texture.compareTo(Texture other)`             Compares this Texture object to the Texture object given as an argument by making a lexicographic comparison of their menu names. |

| Uses of Texture in org.proteinshader.gui | |
| --- | --- |

| Methods in org.proteinshader.gui that return types with arguments of type Texture | |
| --- | --- |
| `Vector<Texture>` | `MediatorImpl.getBendTextures()`             Returns the list of Textures intended for highlighting segment bend regions when halftoning is being used. |
| `Vector<Texture>` | `Mediator.getBendTextures()`             Returns the list of Textures intended for highlighting segment bend regions when halftoning is being used. |
| `Vector<Texture>` | `MediatorImpl.getHalftoningTextures()`             Returns the list of Texture objects intended for halftoning. |
| `Vector<Texture>` | `Mediator.getHalftoningTextures()`             Returns the list of Texture objects intended for halftoning. |
| `Vector<Texture>` | `MediatorImpl.getPatternsTextures()`             Returns the list of Texture objects intended for placing patterns on colored surfaces (rather than textures for halftoning). |
| `Vector<Texture>` | `Mediator.getPatternsTextures()`             Returns the list of Texture objects intended for placing patterns on colored surfaces (rather than textures for halftoning). |

---


|  |  |  |  |  |  |  |  |  |  |  |
| --- | --- | --- | --- | --- | --- | --- | --- | --- | --- | --- |
| |  |  |  |  |  |  |  |  | | --- | --- | --- | --- | --- | --- | --- | --- | | **Overview** | **Package** | **Class** | **Use** | **Tree** | **Deprecated** | **Index** | **Help** | | |  |
| PREV   NEXT | **FRAMES**    **NO FRAMES**     **All Classes** |


---

# *Copyright © 2007-2008*
