# Supplementary material for: ProteinShader: illustrative rendering of macromolecules
Source: BMC Struct Biol. 2009 Mar 30;9:19. doi: 10.1186/1472-6807-9-19 (PMC2672931; doi:10.1186/1472-6807-9-19)
Supplement: Additional file 1 — ProteinShader program without source code. This compressed file contains the complete ProteinShader program including associated libraries, but no source code. A README.txt file gives an overview of the ProteinShader distribution, and the index.html file in the help subdirectory has directions on getting started with the program as well as a set of tutorials. [file 1472-6807-9-19-S1.zip › ProteinShader-beta-0_9_4-binary/help/api/org/proteinshader/graphics/textures/class-use/NamePair.html]

Uses of Class org.proteinshader.graphics.textures.NamePair (ProteinShader API)


|  |  |  |  |  |  |  |  |  |  |  |
| --- | --- | --- | --- | --- | --- | --- | --- | --- | --- | --- |
| |  |  |  |  |  |  |  |  | | --- | --- | --- | --- | --- | --- | --- | --- | | **Overview** | **Package** | **Class** | **Use** | **Tree** | **Deprecated** | **Index** | **Help** | | |  |
| PREV   NEXT | **FRAMES**    **NO FRAMES**     **All Classes** |


---


## **Uses of Class org.proteinshader.graphics.textures.NamePair**

| Packages that use NamePair | |
| --- | --- |
| **org.proteinshader.graphics.textures** | Holds the classes needed for creating and managing OpenGL texture objects. |

| Uses of NamePair in org.proteinshader.graphics.textures | |
| --- | --- |

| Methods in org.proteinshader.graphics.textures that return types with arguments of type NamePair | |
| --- | --- |
| `List<NamePair>` | `ConfigReader.readConfigFile(File file)`             Reads a configuration file to obtain a list of menu names with a matching filename for each menu name. |
| `List<NamePair>` | `EqualSignConfigReader.readConfigFile(File file)`             Reads a configuration file to obtain a list of menu names with a matching filename for each menu name. |
| `List<NamePair>` | `ConfigReader.readConfigFile(String filename)`             Reads a configuration file to obtain a list of menu names with a matching filename for each menu name. |
| `List<NamePair>` | `EqualSignConfigReader.readConfigFile(String filename)`             Reads a configuration file to obtain a list of menu names with a matching filename for each menu name. |

---


|  |  |  |  |  |  |  |  |  |  |  |
| --- | --- | --- | --- | --- | --- | --- | --- | --- | --- | --- |
| |  |  |  |  |  |  |  |  | | --- | --- | --- | --- | --- | --- | --- | --- | | **Overview** | **Package** | **Class** | **Use** | **Tree** | **Deprecated** | **Index** | **Help** | | |  |
| PREV   NEXT | **FRAMES**    **NO FRAMES**     **All Classes** |


---

# *Copyright © 2007-2008*
