# Supplementary material for: ProteinShader: illustrative rendering of macromolecules
Source: BMC Struct Biol. 2009 Mar 30;9:19. doi: 10.1186/1472-6807-9-19 (PMC2672931; doi:10.1186/1472-6807-9-19)
Supplement: Additional file 1 — ProteinShader program without source code. This compressed file contains the complete ProteinShader program including associated libraries, but no source code. A README.txt file gives an overview of the ProteinShader distribution, and the index.html file in the help subdirectory has directions on getting started with the program as well as a set of tutorials. [file 1472-6807-9-19-S1.zip › ProteinShader-beta-0_9_4-binary/help/api/org/proteinshader/graphics/textures/ConfigReader.html]

ConfigReader (ProteinShader API)


|  |  |  |  |  |  |  |  |  |  |  |
| --- | --- | --- | --- | --- | --- | --- | --- | --- | --- | --- |
| |  |  |  |  |  |  |  |  | | --- | --- | --- | --- | --- | --- | --- | --- | | **Overview** | **Package** | **Class** | **Use** | **Tree** | **Deprecated** | **Index** | **Help** | | |  |
| PREV CLASS   **NEXT CLASS** | **FRAMES**    **NO FRAMES**     **All Classes** |
| SUMMARY: NESTED | FIELD | CONSTR | METHOD | DETAIL: FIELD | CONSTR | METHOD |


---


## org.proteinshader.graphics.textures Interface ConfigReader

**All Known Implementing Classes:**: EqualSignConfigReader

---

``` public interface ConfigReader ```

Reads a configuration file to obtain a list of menu names
and matching filenames for textures.

---

| **Method Summary** | |
| --- | --- |
| `List<NamePair>` | `readConfigFile(File file)`             Reads a configuration file to obtain a list of menu names with a matching filename for each menu name. |
| `List<NamePair>` | `readConfigFile(String filename)`             Reads a configuration file to obtain a list of menu names with a matching filename for each menu name. |

| **Method Detail** |
| --- |

### readConfigFile

```
List<NamePair> readConfigFile(String filename)
                              throws TextureException
```

:   Reads a configuration file to obtain a list of menu names with a matching
    filename for each menu name.

    :   **Parameters:**: `filename` - the config file to read. **Returns:**: A list of menu name and filename pairs. **Throws:**: `TextureException` - if the config file cannot be read.

---


### readConfigFile

```
List<NamePair> readConfigFile(File file)
                              throws TextureException
```

:   Reads a configuration file to obtain a list of menu names with a matching
    filename for each menu name.

    :   **Parameters:**: `file` - the config file to read. **Returns:**: A list of menu name and filename pairs. **Throws:**: `TextureException` - if the config file cannot be read.


---


|  |  |  |  |  |  |  |  |  |  |  |
| --- | --- | --- | --- | --- | --- | --- | --- | --- | --- | --- |
| |  |  |  |  |  |  |  |  | | --- | --- | --- | --- | --- | --- | --- | --- | | **Overview** | **Package** | **Class** | **Use** | **Tree** | **Deprecated** | **Index** | **Help** | | |  |
| PREV CLASS   **NEXT CLASS** | **FRAMES**    **NO FRAMES**     **All Classes** |
| SUMMARY: NESTED | FIELD | CONSTR | METHOD | DETAIL: FIELD | CONSTR | METHOD |


---

# *Copyright © 2007-2008*
