# Supplementary material for: ProteinShader: illustrative rendering of macromolecules
Source: BMC Struct Biol. 2009 Mar 30;9:19. doi: 10.1186/1472-6807-9-19 (PMC2672931; doi:10.1186/1472-6807-9-19)
Supplement: Additional file 2 — ProteinShader program with source code. This compressed file contains everything in the binary distribution plus the Java source code and a build.xml file for compiling with Ant. [file 1472-6807-9-19-S2.gz › ProteinShader-beta-0_9_4-src/src/org/proteinshader/gui/listeners/menubar/package.html]

Holds the listeners for the items used in the JMenus of the menu bar above
the canvas.
  
  

## Related Documentation

For help and tutorial pages, please see:

- http://proteinshader.sourceforge.net

@author Joseph R. Weber
@version Beta 0.9.4 (December 30, 2008)
