# Supplementary material for: ProteinShader: illustrative rendering of macromolecules
Source: BMC Struct Biol. 2009 Mar 30;9:19. doi: 10.1186/1472-6807-9-19 (PMC2672931; doi:10.1186/1472-6807-9-19)
Supplement: Additional file 2 — ProteinShader program with source code. This compressed file contains everything in the binary distribution plus the Java source code and a build.xml file for compiling with Ant. [file 1472-6807-9-19-S2.gz › ProteinShader-beta-0_9_4-src/src/org/overview.html]

The ProteinShader program is a free open-source platform-independent molecular
visualization tool designed to produce illustrative renderings of proteins
that approximate what an artist might create using pen and ink.
  
  
ProteinShader is written primarily in Java, and uses JOGL (Java Bindings for
OpenGL) to call on the C language functions of OpenGL, a low-level graphics
library that is supported by most modern graphics cards. The texture
mapping and lighting calculations needed by ProteinShader are accomplished
using custom vertex and fragement shaders written in the OpenGL Shading
Language. The source code for the vertex and fragment shaders can be found
in the `shaders` directory of the ProteinShader distribution, while
the Java source code documented in this API can be found in the
`src` directory.

## Related Documentation

For help and tutorial pages, please see:

- http://proteinshader.sourceforge.net

@author Joseph R. Weber
@version Beta 0.9.4 (December 30, 2008)
